# Sustainable Recovery of Dibutyl Terephthalate from Aromatic Polyester Waste via Solvothermal Alcoholysis

**DOI:** 10.1021/acs.inorgchem.6c01014

**Published:** 2026-08-21

**Authors:** Karolina Matuszak, Rafał Petrus, Tadeusz Lis

**Affiliations:** † Faculty of Chemistry, 49567 Wrocław University of Science and Technology, 23 Smoluchowskiego, Wrocław 50-370, Poland; ‡ Faculty of Chemistry, University of Wrocław, 14 F. Joliot-Curie, Wrocław 50-383, Poland

## Abstract

In this work, a series of homometallic and heterometallic aryloxides, including [M′_6_(sal-Me)_6_] (M′^+^ = Li^+^ (**1**), Na^+^ (**2**), K^+^ (**3**)), [M′(sal-Me)]*_n_* (M′^+^ = Rb^+^ (**4**), Cs^+^ (**5**)), [Mg_2_(sal-Et)_4_(EtOH)_2_] (**6**), [Zn_4_(sal-Me)_8_] (**7**), [Ca_3_(sal-Me)_6_(EtOH)_2_] (**8**), [M_2_M′_2_(sal-Me)_6_(X)_*x*_] (where M^2+^ = Mg^2+^ and M′^+^ = Li^+^ (**9**), Na^+^ (**10**), K^+^ (**11**); M^2+^ = Zn^2+^ and M′^+^ = Li^+^ (**13**), Na^+^ (**14**), K^+^ (**15**); or M^2+^ = Ca^2+^ and M′^+^ = Li^+^ (**16**), Na^+^ (**17**), K^+^ (**18**) with X = THF or MeOH and *x* = 0, 2, 4), [MgRb­(μ-H_2_O)­(sal-Me)_3_(H_2_O)_0.5_]_*n*_ (**12**), [Mg_4_Na_2_(sal-Me)_6_(sal)_2_(THF)_4_] (**19**), [CaLi_6_(sal-Me)_8_] (**20**), [Ca_4_M′(μ_5_-OH)­(sal-Et)_8_(EtOH)] (M′^+^ = Na^+^ (**21**), K^+^ (**22**)), [ZnLi_2_(sal-Me)_4_(THF)_2_] (**23**), and [ZnM′_2_(sal-R)_4_]_*n*_ (for M′ = Na and R = Et (**24**), M′ = K and R = Me (**25**)), were investigated as catalysts for the chemical recycling of postconsumer poly­(ethylene terephthalate) (PET) and poly­(ethylene-*co*-isosorbide-*co*-1,4-cyclohexanedimethylene terephthalate) (PEICT) waste. The catalytic performance of metal aryloxides was evaluated in relation to their chemical composition, solid-state structures, and solution behavior, allowing general trends in the catalytic activity of alkali metal and divalent metal aryloxides (Mg^2+^, Ca^2+^, Zn^2+^) and their heterometallic combinations to be established. Key factors governing catalytic activity, reaction mechanisms, and the formation of reaction intermediates are also discussed.

## Graphical abstract

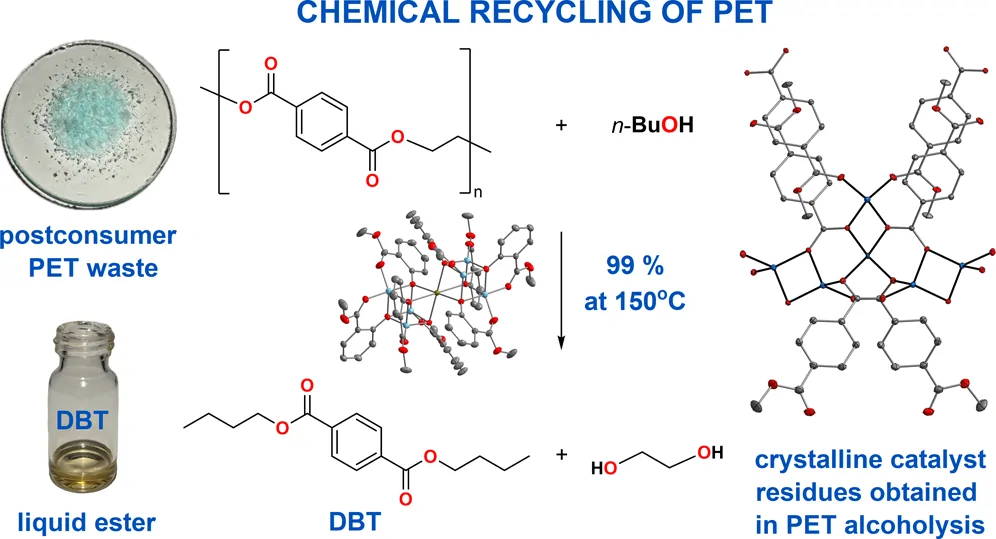

## Introduction

Poly­(ethylene terephthalate) (PET) is the fourth most commonly used plastic, with primary applications in synthetic fiber manufacture (over 60%) and thermoforming, often in combination with engineering resins and glass fibers. It is also one of the most important raw materials for food trays and packaging films, as well as for the production of beverage bottles (ca. 30%), owing to its excellent glass-like transparency, high toughness-to-weight ratio, good gas barrier properties, and corrosion resistance.^1^ Industrially, PET is produced via the reaction of terephthalic acid (TAH_2_) or dimethyl terephthalate (DMT) with ethylene glycol (EG), followed by direct polycondensation of the resulting bis­(2-hydroxyethyl) terephthalate (BHET) and low-molecular-weight oligomers.^2^

The growing accumulation of PET waste has negative impacts on both human health and ecological systems, and various recycling technologies have been developed to mitigate these adverse effects. The most common approach is mechanical recycling, which is economically viable and involves sorting, cleaning, shredding, melting, and re-extruding plastic waste into pellets. This method performs well for specific waste streams, such as bottles or containers, and is suitable for producing durable materials for applications different from the original ones.^3^ However, mechanically recycled PET from trays is often affected by organic impurities and may contain multilayer material. Additional limitations include reduced molecular weight and yellowing caused by thermal degradation. The production of food-contact articles is particularly challenging, especially when recyclers have limited control over the waste stream.^4^ An alternative approach is chemical depolymerization, which involves breaking polymer chains into monomers or intermediates via chemical reactions. The resulting molecules can then be reused to produce polymers of the same grade and quality.^5^ To date, several chemical methods for PET degradation have been reported, including pyrolysis,^6–8^ hydrolysis,^9–13^ aminolysis,^14–18^ hydrogenolysis,^19–22^ and alcoholysis.^2,23–26^ Alcoholysis employs alcohols as solvents and reagents to depolymerize PET into monomers. Among the reported processes, methanolysis and glycolysis clearly dominate, reflecting the central role of DMT and BHET as key intermediates in the synthesis of phthalate polyesters. In general, PET alcoholysis is strongly dependent on pressure, temperature, reaction time, PET particle size and surface area, and the PET/ROH ratio.^27,28^ Uncatalyzed reactions require harsh conditions and long reaction times; for example, efficient depolymerization using supercritical alcohols has been reported for MeOH (300–330 °C, 8.1–25 MPa, 0.5–1.2 h, 45–97%),^29–31^ or EtOH (255–310 °C, 1–5 h, >16.5 MPa, 66–98%).^32,33^ In contrast, BHET synthesis via glycolysis can be achieved under milder conditions (190–200 °C, 8 h, EG/PET = 2.8/1), affording up to 99% yield.^34^ Catalyst loading has a substantial impact on reaction outcomes, and PET alcoholysis is commonly carried out using homogeneous catalysts, including sulfonic acids,^35^ guanidine derivatives,^36–38^ ionic liquids,^39^ or simple metal salts,^40–42^ to overcome the high thermal stability of aromatic ester bonds and promote efficient depolymerization. Metal acetates were among the earliest catalysts used for PET alcoholysis.^22^ For example, {Zn/Pb}­(OAc)_2_ converts 98% PET to DMT at 120 °C under 0.71 MPa within 2 h (PET/MeOH/M = 1/14.3/0.02).^43^ Other reported catalysts include [Ti_4_(μ_3_-OMe)_2_(μ-OPh)_4_(OPh)_10_] (PET/MeOH/Ti = 1/23/0.08, 150 °C, 0.25 h, 100%),^44^ Al­(O^i^Pr)_3_ (PET/MeOH/Al = 1/95/0.001, 200 °C, 2 h, 67%),^45^ LiO*^t^* Bu (PET/MeOH/Li = 1/24/0.1, THF, 65 °C, 5 h, 47%),^46^ and [Zn­(L)_2_] (L^–^ = 2-[(2-aminoethyl)­amino]-4,6-di-*t*-butylphenolato) (PET/MeOH = 1/19, catalyst 8 wt %, MeOH:toluene (4:1), 150 °C, 1.5 h, 72%).^47^ Zn­(OAc)_2_ (PET/EG = 1/3, 196 °C, catalyst 0.2 wt %, 2 h, 100%)^48^ and numerous other metal salts, including ZrCl_4_, CoCl_2_·6H_2_O, ZnSO_4_·7H_2_O, NiCl_2_·6H_2_O, NiSO_4_·7H_2_O, MnCl_2_·4H_2_O, and ZnCl_2_, have also been applied in BHET synthesis (190 °C, 3 h), achieving PET conversions of 62–100% and BHET yields of 45–69%.^49^ The number of coordination compounds investigated for PET glycolysis remains limited. Examples include [TiO­(acac)_2_] (PET/EG/Ti = 1/14/8.6 × 10^–5^, 190 °C, 4 h, 82%),^50^ phenoxy-imine zinc complexes [Zn­(L)_2_] (PET/EG/Zn = 1/28/0.013, 180 °C, 1 h, 95% for L^–^ = 2,4-ditertbutyl-6-({[2-(pyridin-2-yl)­ethyl]­imino}­methyl)­phenolato; PET/EG = 1/27.5, catalyst 8 wt %, 180 °C, 2.5 h, 66% for L^–^ = (2-{[(2-methoxyethyl)­imino]­methyl}­phenolato)),^51,52^ as well as phenoxy-amine zinc complexes (PET/EG = 1/20.6/, catalyst 8 wt %, 180 °C, 1.5 h, 48% for L^–^ = 2,4-dibromo-6-({[3-(dimethylamino)­propyl]­amino}­methyl)­phenolato).^53^ Heterometallic complexes of the type [M_2_K_2_(L)_2_(THF)_2_] (M^2+^ = Mg^2+^ or Zn^2+^; L^3–^ = nitrilotris­[(ethane-2,1-diyl)­azanylylidenemethanylylidene]­triphenolato) were also applied to the depolymerization of postconsumer PET bottles, achieving efficient glycolysis within ≤2 h at 180 °C (PET/EG/cat. = 1/20/0.01).^54^ Several inorganic compounds, such as Ti­(O*^n^* Bu)_4_, CoCl_2_, FeCl_3_, and M­(OAc)_2_ (M^2+^ = Zn^2+^, Mn^2+^, Co^2+^, Cu^2+^), have also been studied for PET degradation using 2-ethyl-1-hexanol (PET/2-EH = 1/4.2, 5 wt % catalyst, 185 °C, 1 h) although these systems generally exhibit lower activity (32–46%) than reactions using shorter-chain alcohols.^23^ Benzyl alcohol, known for its good solubilizing ability toward many synthetic polymers, was also investigated as a reagent for PET alcoholysis in the presence of 5 wt % [Zn­(L)_2_] (for L^–^ = 2,4-di-*tert*-butyl-6-({[3-(dimethylamino)­propyl]­imino}­methyl)­phenolato), affording quantitative conversion at 150 °C (PET/BnOH = 1/15).^55^ Heterogeneous catalysts, mainly based on metal oxides,^56–61^ metal–organic frameworks (MOFs),^62–65^ and zeolites,^66,67^ have also been explored for PET alcoholysis.

Glycolysis of PET to BHET is currently the most widely applied chemical recycling route, as it enables regeneration of virgin-grade material and is being pursued industrially, with several pilot-scale plants in operation. For example, VolCat, a technology developed by IBM, converts PET waste into BHET via solvothermal glycolysis at temperatures above 200 °C using TBD as a catalyst.^37,68^ JEPLAN operates a “bottle-to-bottle” industrial PET chemical recycling process (BRING Technology), producing BHET in 98% yield through a predepolymerization step (220 °C, 1 h) to form oligomers, followed by EG addition and NaOMe catalysis (200 °C, 2 h). Ioniqa employs a magnetic ionic liquid system composed of magnetite/maghemite and [bmim]­[FeCl_4_], enabling degradation of various PET materials (e.g., bottles, carpets, and multilayer trays) at 200 °C (PET/EG = 1/8, 2 wt % catalyst), achieving BHET yields of up to 93%. Since 2019, this technology has operated at a capacity exceeding 10 kilotons per year.^69^ Loop Industries has also developed a PET recycling process based on methanolysis to produce DMT.^70^

Although the aforementioned methods significantly enhance the efficiency of aromatic polyester degradation, the development of low-temperature, low-energy, cost-effective, and highly efficient processes capable of completely converting polymer waste to high-value chemicals remains a major challenge. Moreover, the conventional PET monomer recycling loop does not inherently generate additional economic value. Consequently, integrating PET chemical degradation with upgrading strategies to produce value-added chemicals in a sustainable manner is of considerable interest, offering both economic and environmental benefits.

One such approach involves converting PET into a range of dialkyl terephthalates (DATs), allowing control over alcoholysis pathways and enabling selective production of compounds with tailored physicochemical properties through appropriate reagent selection. Within the DAT family, beyond the widely used intermediates DMT and BHET,^71–73^ particular attention has been given to di­(2-ethylhexyl) terephthalate (DEHT). DEHT is commonly used as a plasticizer in PVC food-contact applications, coatings, sealants, and films due to its good low-temperature flexibility and low volatility.^74,75^ It is also regarded as a safer alternative to *ortho*-phthalate plasticizers because of its reduced toxicity, lower regulatory pressure, and improved migration behavior. DATs have also been reported as ligand precursors for the synthesis of metal carboxylates used as lithium grease components or catalysts.^76,77^

Recovery of terephthalate monomers as liquid diesters, such as dibutyl terephthalate (DBT), offers an economically attractive strategy for recycling unsorted or highly contaminated polymer waste streams. Conventional PET glycolysis typically yields BHET and oligomers, whereas alcoholysis with *n*-BuOH selectively produces DBT and EG. DBT is a pale-yellow, transparent liquid with a high boiling point (342 °C), low volatility, and excellent compatibility with various polymers, making it particularly suitable for applications requiring enhanced flexibility without compromising thermal stability, such as PVC flooring, adhesives, sealants, and printing inks.^78,79^ DBT is generally less reactive and more thermally stable than DMT, making it more resistant to hydrolysis to terephthalic acid and therefore more suitable for applications in high-humidity environments. Additionally, liquid DBT may be used directly as a cosolvent and reagent in polycondensation reactions with diols. Although DBT is industrially produced via esterification of TAH_2_, its synthesis from PET waste represents a more sustainable alternative.

The objective of this work is to develop technologies that enable efficient recycling of PET and other terephthalate polymers from unsorted, contaminated, and mixed plastic waste streams without prior removal of impurities such as dirt, paper labels, or adhesives. Herein, a series of homometallic and heterometallic aryloxide complexes, including [M′_6_(sal-Me)_6_] (M′^+^ = Li^+^ (**1**), Na^+^ (**2**), K^+^ (**3**)), [M′(sal-Me)]*_n_* (M′^+^ = Rb^+^ (**4**), Cs^+^ (**5**)), [Mg_2_(sal-Et)_4_(EtOH)_2_] (**6**), [Zn_4_(sal-Me)_8_] (**7**), [Ca_3_(sal-Me)_6_(EtOH)_2_] (**8**), [M_2_M′_2_(sal-Me)_6_(X)_*x*_] (where M^2+^ = Mg^2+^ and M′^+^ = Li^+^ (**9**), Na^+^ (**10**), K^+^ (**11**); M^2+^ = Zn^2+^ and M′^+^ = Li^+^ (**13**), Na^+^ (**14**), K^+^ (**15**); or M^2+^ = Ca^2+^ and M′^+^ = Li^+^ (**16**), Na^+^ (**17**), K^+^ (**18**) with X = THF or MeOH and *x* = 0, 2, 4), [MgRb­(μ-H_2_O)­(sal-Me)_3_(H_2_O)_0.5_]*_n_* (**12**), [Mg_4_Na_2_(sal-Me)_6_(sal)_2_(THF)_4_] (**19**), [CaLi_6_(sal-Me)_8_] (**20**), [Ca_4_M′(μ_5_-OH)­(sal-Et)_8_(EtOH)] (M′^+^ = Na^+^ (**21**), K^+^ (**22**)), [ZnLi_2_(sal-Me)_4_(THF)_2_] (**23**), and [ZnM′_2_(sal-R)_4_]_*n*_ (for M′ = Na and R = Et (**24**), M′ = K and R = Me (**25**)), were investigated as catalysts for recycling postconsumer PET and PEICT waste.

## Results and Discussion

### Synthesis and Structural Characterization of Homometallic and Heterometallic Alkali Metal-Alkaline Earth or Transition-Metal Aryloxides

Our previous studies established the synthesis and structural or spectroscopic characterization of a series of homometallic aryloxides, including [M′_6_(sal-Me)_6_] (M′^+^ = Li^+^ (**1**), Na^+^ (**2**), K^+^ (**3**)), [Mg_2_(sal-Et)_4_(EtOH)_2_] (**6**), and [Zn_4_(sal-Me)_8_] (**7**).^80,81^ In the present work, this family of compounds was further expanded to include rubidium and cesium aryloxides [M′(sal-Me)]*_n_* (M′^+^ = Rb^+^ (**4**), Cs^+^ (**5**)), as well as the trinuclear calcium complex [Ca_3_(sal-Me)_6_(EtOH)_2_] (**8**), shown in Scheme 1. Compounds **4** and **5** crystallize in the orthorhombic *Pccn* space group, forming one-dimensional coordination polymers [M′(sal-Me)]*_n_*, featuring μ_3_-O_(aryloxo)_ and μ-O_(carbonyl)_ bridging groups. However, ^1^H, ^13^C, and ^1^H-DOSY NMR measurements revealed the formation of penta- or hexanuclear aggregates in THF-*d*_8_ solution (Figures S1–S8, Table S2). The crystal structure of **8** is isostructural with the recently reported [Ca_3_(sal-Me)_6_(MeOH)_2_] (Figures S9–S12).^82^ In this study, the homometallic aryloxides served as key precursors for the synthesis of heterometallic complexes comprising alkali metal ions and divalent metal cations (Mg^2+^, Zn^2+^, and Ca^2+^), as summarized in Scheme 2.

**Scheme 1:**
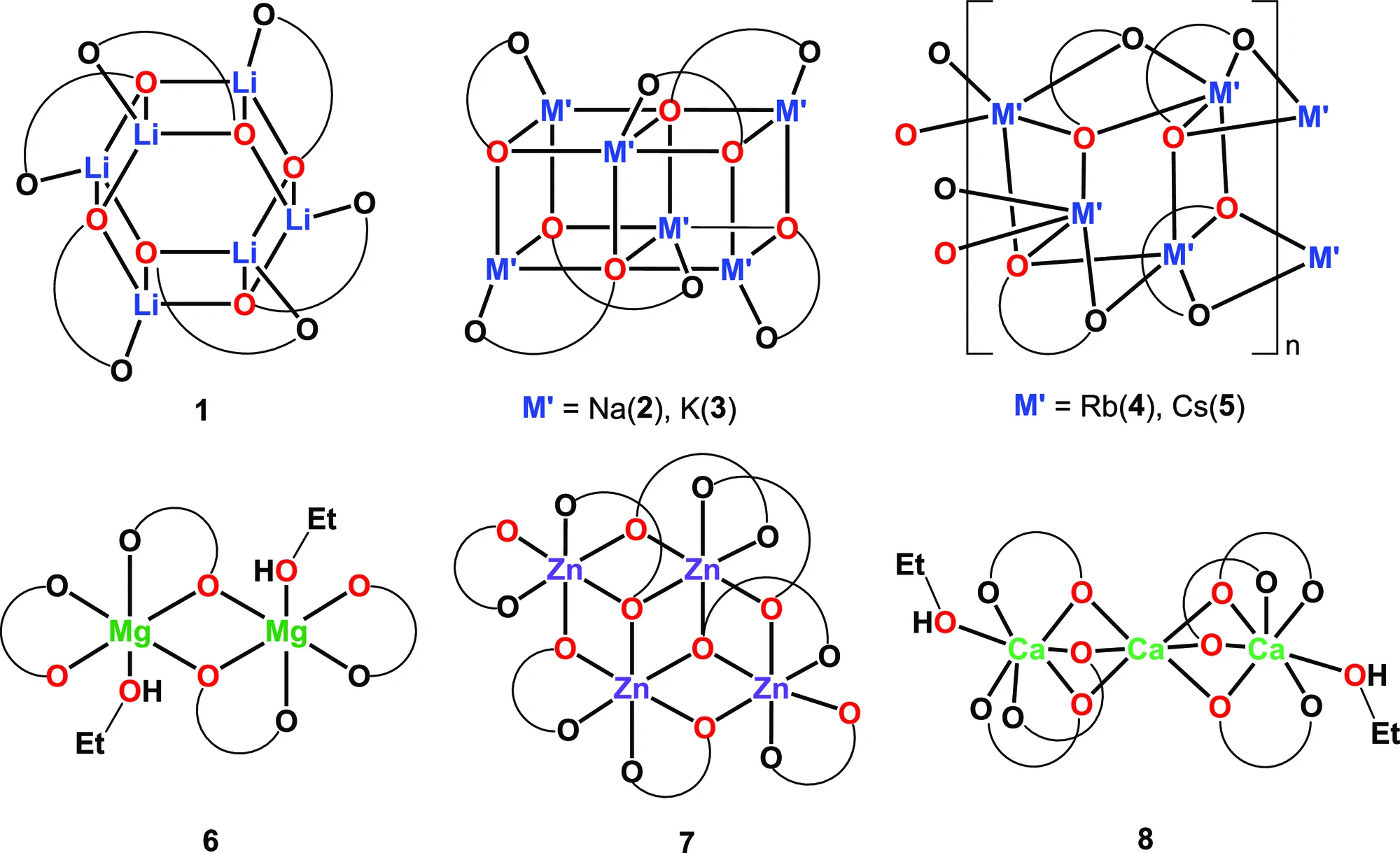
Structures of Investigated Homometallic Aryloxides **1**–**8**

**Scheme 2:**
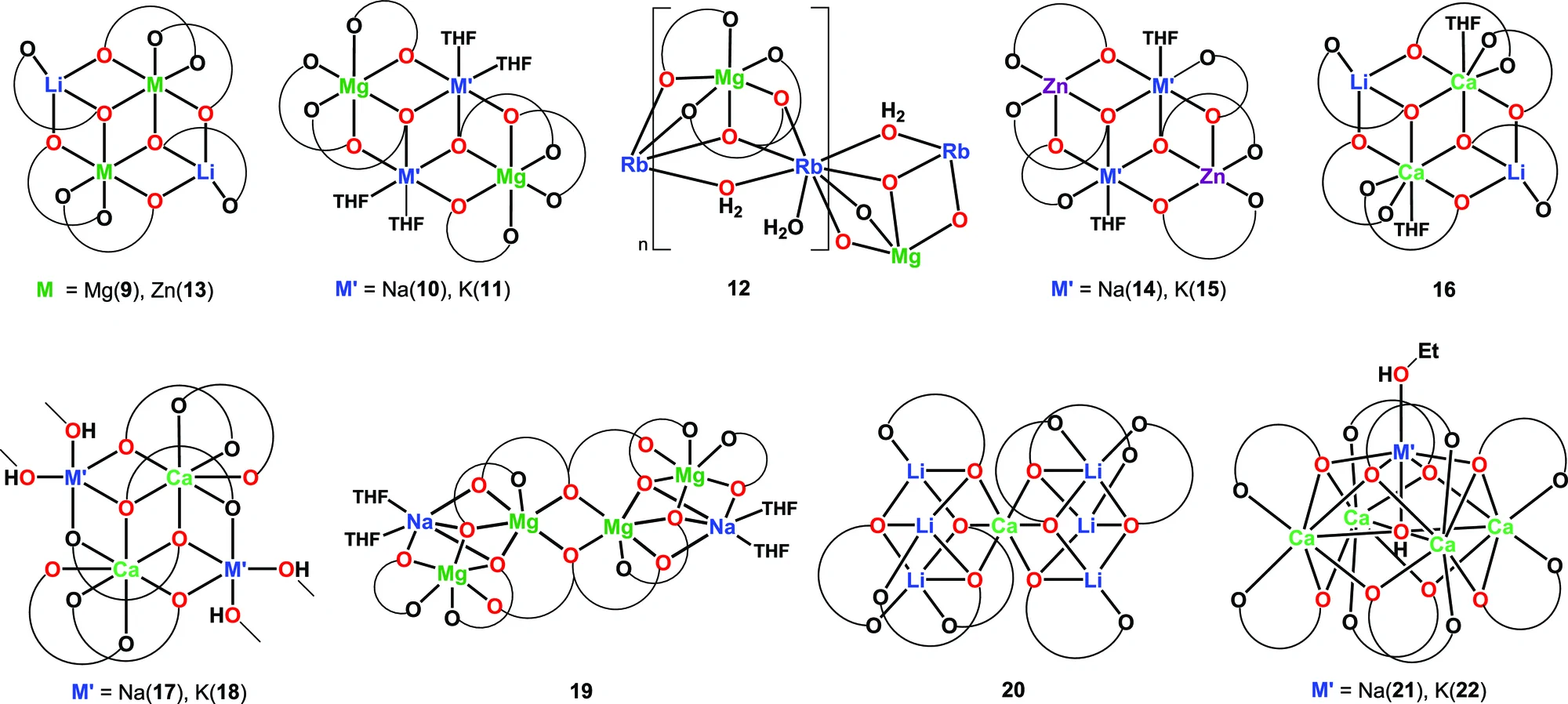
Structures of Investigated Heterometallic Aryloxides **9**–**22**

The predominant class of heterometallic aryloxides consists of centrosymmetric tetranuclear complexes based on a double-opened dicubane core with two missing vertices, [M_2_M′_2_(sal-Me)_6_(X)_*x*_] (M^2+^ = Mg^2+^ and M′^+^ = Li^+^ (**9**), Na^+^ (**10**), K^+^ (**11**); M^2+^ = Zn^2+^ and M′^+^ = Li^+^ (**13**), Na^+^ (**14**), K^+^ (**15**); or M^2+^ = Ca^2+^ and M′^+^ = Li^+^ (**16**), Na^+^ (**17**), K^+^ (**18**); X = THF or MeOH; *x* = 0, 2, 4).^82,83^ In these structures, the metal atoms are held together by two μ_3_-O and four μ-O aryloxo oxygen atoms. In compounds **9**, **13**, and **16**, the vertices of the shared face of the defective dicubane units are occupied by M^2+^ ions, while Li^+^ ions occupy the peripheral positions. A similar arrangement, with internal Ca^2+^ ions and external Na^+^ or K^+^ ions, was observed for compounds **17** and **18**. In contrast, compounds **10** and **11**, as well as **14** and **15**, exhibit an inverted metal distribution, with Mg^2+^ or Zn^2+^ ions located at the periphery and Na^+^ or K^+^ ions occupying the vertices of the common face. Heterometallic aryloxides such as [Mg_4_Na_2_(sal-Me)_6_(sal)_2_(THF)_4_] (**19**) and [Ca_4_M′(μ_5_-OH)­(sal-Et)_8_(EtOH)] (M′^+^ = Na^+^ (**21**), K^+^ (**22**)) represent structurally uncommon species formed either upon exposure of the reaction mixtures to atmospheric moisture or through deliberate addition of water.^82,84^ Compound **21** is isostructural with the recently reported [Ca_4_Na­(μ_5_-OH)­(sal-Et)_8_(MeOH)] (Figure S13).^82^ Within this family of heterometallic complexes, [Mg_2_M′_2_(sal-Me)_6_(THF)_*x*_] (**9–11**) exhibited notable catalytic activity in the chemical recycling of high-consistency silicone rubber waste via alcoholysis to low-molecular-weight alkoxysilane derivatives.^83^ Binary metal catalysts [Zn_2_M′_2_(sal-Me)_6_(THF)_*x*_] (**13–15**) and [CaLi_6_(sal-Me)_8_] (**20**) showed the highest catalytic activity in the methanolysis of nylon-6 at 220 °C.^82^

Continuation of the synthetic efforts toward heterometallic complexes with an M:M′ stoichiometry of 1:1 led to the isolation of the one-dimensional coordination polymers [MgRb­(μ-H_2_O)­(sal-Me)_3_(H_2_O)_0.5_]*_n_* (**12**) and [Zn_2_Na_2_(sal-R)_6_]_*n*_ (**14a**, for R = Me (0.86), Et (0.14)) (Figures 1 and 2). Structurally, compound **12** represents a rare example of a heterometallic magnesium–rubidium complex; to date, only five related compounds combining these two metals have been reported.^85–88^ In compound **12**, octahedrally coordinated Mg^2+^ ions, bound by three sal-Me ligands, are connected to Rb^+^ ions through two aryloxy oxygen atoms and one carbonyl oxygen atom (Table S3).

**Figure 1:**
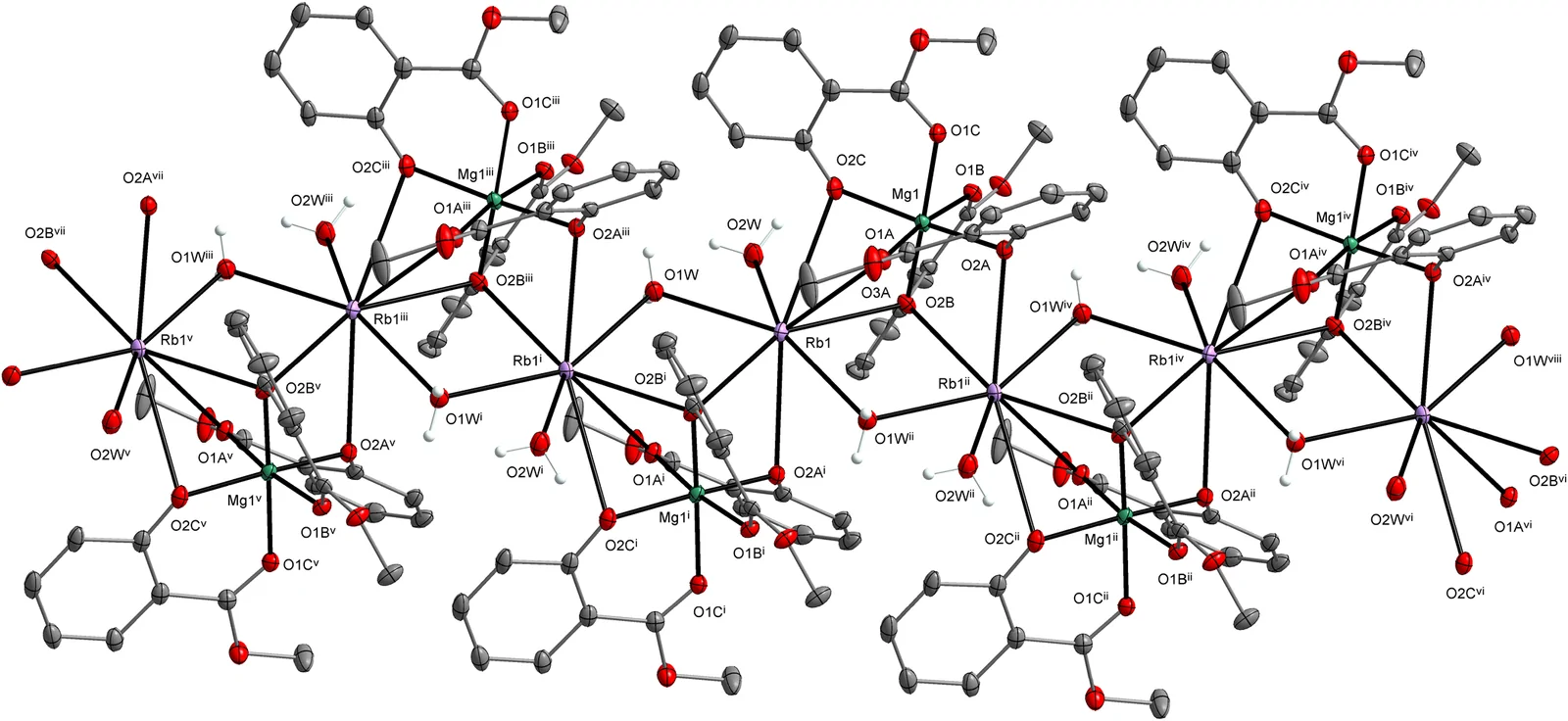
Molecular structure of [MgRb­(μ-H_2_O)­(sal-Me)_3_(H_2_O)_0.5_]*_n_* (**12**). Displacement ellipsoids are drawn at the 25% probability level [symmetry code: (i) 1-*x*, 0.5+*y*, 1-*z*; (ii) 1-*x*, −0.5+*y*, 1-*z*; (iii) *x*, 1+*y*, *z*; (iv) *x*, −1+*y*, *z*; (v) 1-*x*, 1.5+*y*, 1-*z*; (vi) 1-*x*, −1.5+*y*, 1-*z*; (vii) *x*, 2+*y*, *z*; (viii) *x*, −2+*y*, *z*]. Hydrogen atoms are omitted for clarity.

**Figure 2:**
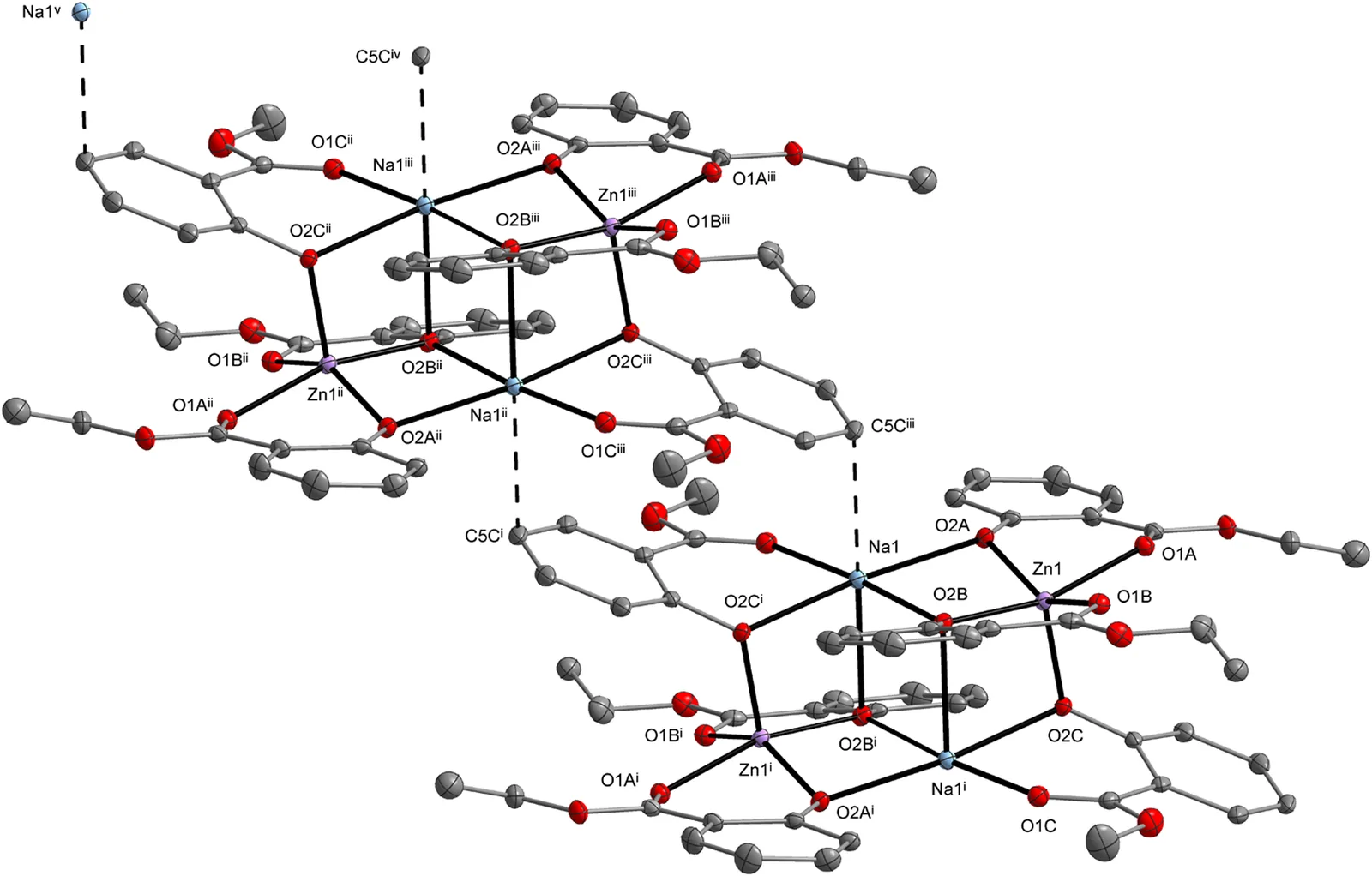
Molecular structure of [Zn_2_Na_2_(sal-R)_6_]*_n_* (**14a**, for R = Me (0.86), Et (0.14)). Displacement ellipsoids are drawn at the 25% probability level [symmetry code: (i) 1-*x*, 2-*y*, 1-*z*; (ii) -*x*, 2-*y*, 1-*z*; (iii) −1+*x*, *y*, *z*; (iv) −2+*x*, *y*, *z*; (v) −1-*x*, 2-*y*, 1-*z*]. Hydrogen atoms are omitted for clarity.

Adjacent Rb^+^ ions, coordinated by a half-occupied terminal H_2_O molecule and a bridging H_2_O molecule, form an infinite chain extending along the [010] 2-fold screw axis. The presence of bridging H_2_O molecules between Rb^+^ ions is characteristic of such systems and has been observed in numerous related structures.^89–91^ Compound **14a** provides an interesting molecular model illustrating the transformation of **14** upon removal of coordinated THF ligands under vacuum, resulting in the formation of a one-dimensional coordination polymer sustained by Na^+^···π interactions, with nonbonding Na1···C5C^iii^ distances of 2.904(2) Å (Figure 2). Compounds **12** and **14a** were characterized by ^1^H, ^13^C NMR, and FTIR-ATR spectroscopy (Figures S14–S19). In addition, ^1^H-DOSY NMR studies revealed that the coordination polymer network of **12** dissociates in THF-*d*_8_ solution to form a heterometallic tetranuclear aggregate analogous to those observed for compounds **10** and **11** (Figure S20 and Table S2). Similarly, the solid-state structure of **14a** is not retained in solution and likely converts into THF-solvated dinuclear species corresponding to half of the tetranuclear complex **14** (Figure S21 and Table S2). These observations led us to further investigate compounds **13**–**15** by ^1^H-DOSY NMR to verify the stability of the tetranuclear structure in solution.

The results showed that compound **13** converts in solution to compound **7** and the trinuclear species [ZnLi_2_(sal-Me)_4_], while compounds **14** and **15** form dinuclear structures, similar to compound **14a** (Figures S22–S24 and Table S2).

When heterometallic complexes were synthesized using Mg*^n^* Bu_2_ or ZnEt_2_ with an M:M′ stoichiometry of 1:2, mixtures of homometallic aryloxides **1**–**3** and heterometallic species **9**–**11** were obtained, along with the trinuclear compound [ZnLi_2_(sal-Me)_4_(THF)_2_] (**23**, 30%) and the one-dimensional coordination polymers [ZnM′_2_(sal-R)_4_]_*n*_ (for M′ = Na and R = Et (**24**, 36%), M′ = K and R = Me (**25**, 91%)), as shown in Scheme 3. FTIR-ATR spectroscopy and powder X-ray diffraction (PXRD) analyses (Figures 3, 4 and S25–S28), compared with reference data for **1**–**3** and **9**–**11**, indicate that variation of reaction stoichiometry does not lead to new heterometallic magnesium–alkali metal aggregates, but instead favors formation of open dicubane heterometallic complexes alongside homometallic aryloxides.

**Figure 3:**
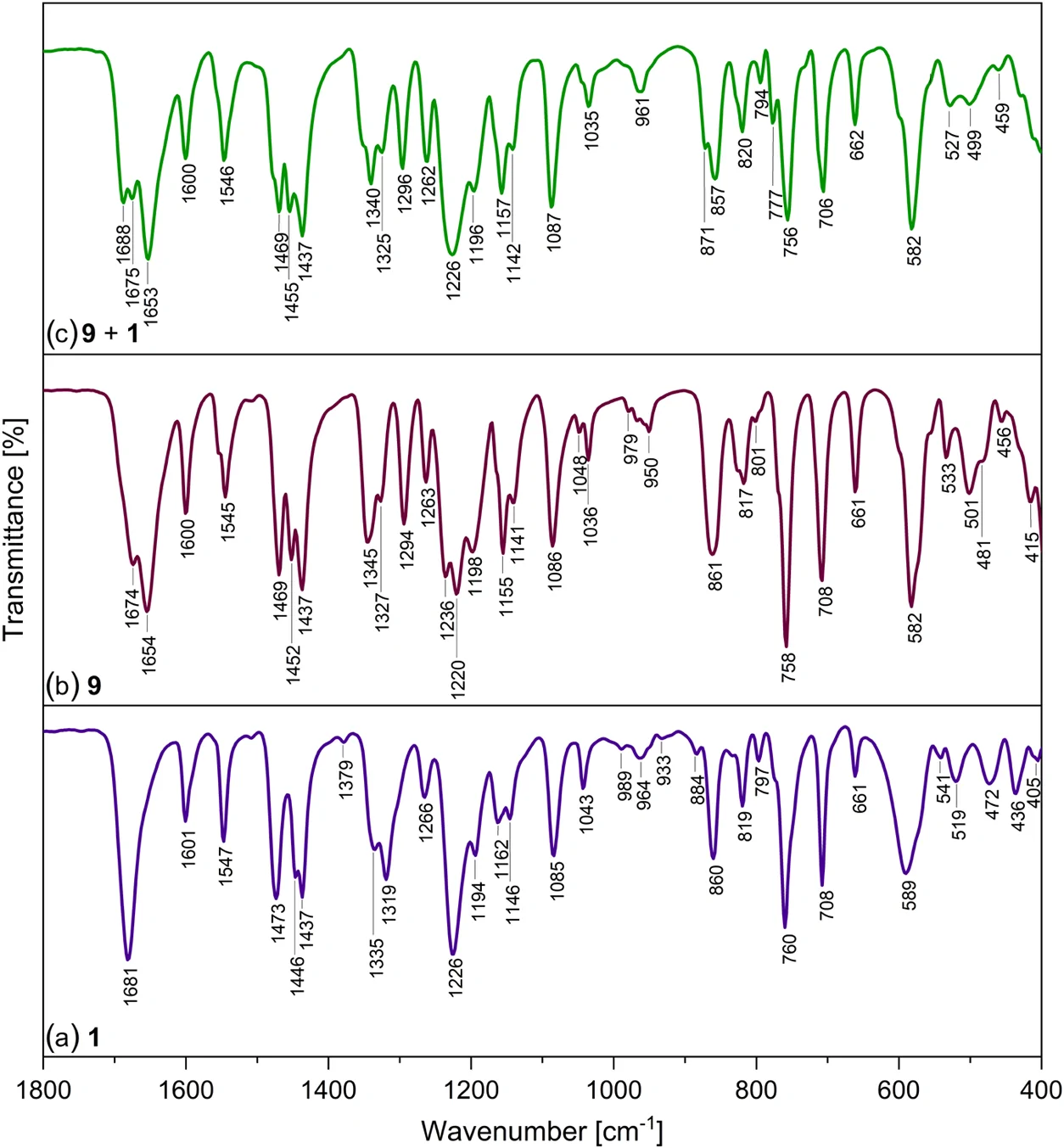
Comparison of FTIR-ATR spectra of reference compounds **1** (a) and **9** (b) with the spectrum of the product obtained from the reaction of Mg*^n^* Bu_2_, Li, and Hsal-Me (1:2:4) (c).

**Figure 4:**
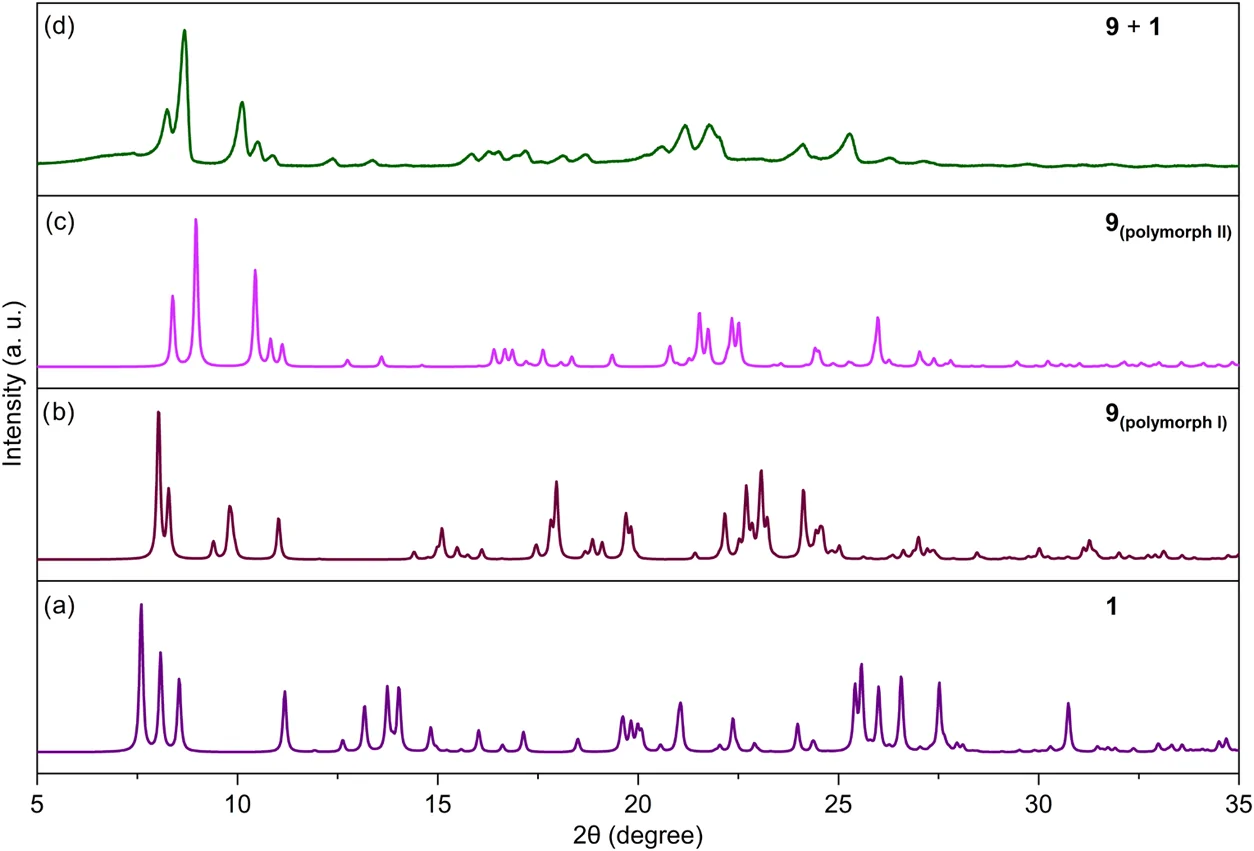
Comparison of PXRD patterns simulated from crystal structures of **1** (a) and **9** (b, c) with the experimental PXRD pattern of the product obtained from the reaction of Mg*^n^* Bu_2_, Li, and Hsal-Me (1:2:4) (d).

**Scheme 3:**
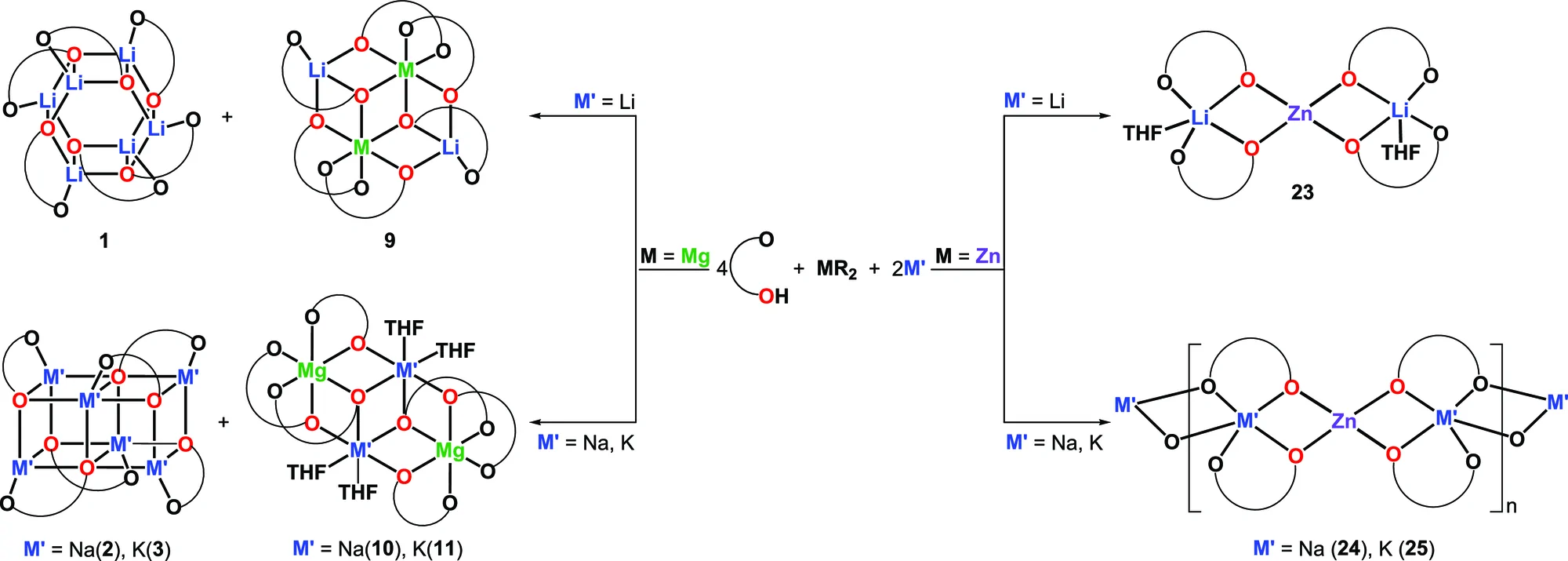
Synthesis of **23**–**25**

During these studies, a new monoclinic polymorph of compound **9** and a triclinic polymorph of compound **11** were also identified (Figures S29 and S30).

The molecular structure of compound **23** (Figure 5) features a tetrahedrally coordinated Zn^2+^ ion linked to two Li^+^ ions via four aryloxide oxygen bridges. Each Li^+^ ion is coordinated by two chelating sal-Me ligands and one THF molecule, adopting a square-pyramidal geometry (Table S3). To the best of our knowledge, this central core motif has only been previously reported for [ZnLi_2_(L)­(THF)_4_] (L^2–^ = 3,3′,5,5′-tetra-*t*-butylbiphenyl-2,2′-diolato).^92^ In turn, the molecular structure of [ZnNa_2_(sal-Et)_4_]_*n*_ (**24**), shown in Figure 6, represents a rare example of a heterometallic zinc–alkali metal phenolato coordination polymer, as analogous systems are more commonly constructed using carboxylate ligands.^93^ In the polymeric chain of **24**, tetrahedrally coordinated Zn^2+^ ions are separated by two square-pyramidal Na^+^ ions bridged through carbonyl oxygen atoms (Table S3). For alkyl salicylate ligands, the presence of carbonyl groups bridging metal ions has previously been observed only in [MgK­(sal-Me)_3_]*_n_* and [ZnNa_2_(sal-Me)_4_]_*n*_.^83,84^

**Figure 5:**
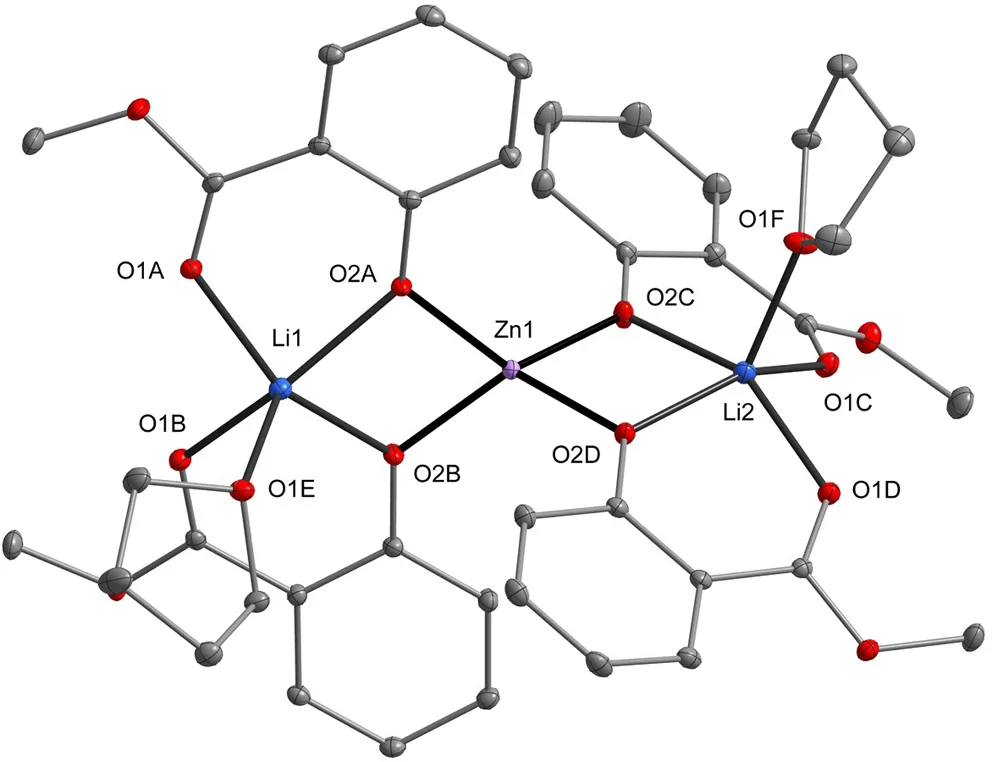
Molecular structure of [ZnLi_2_(sal-Me)_4_(THF)_2_] (**23**). Displacement ellipsoids are drawn at the 25% probability level. Hydrogen atoms are omitted for clarity.

**Figure 6:**
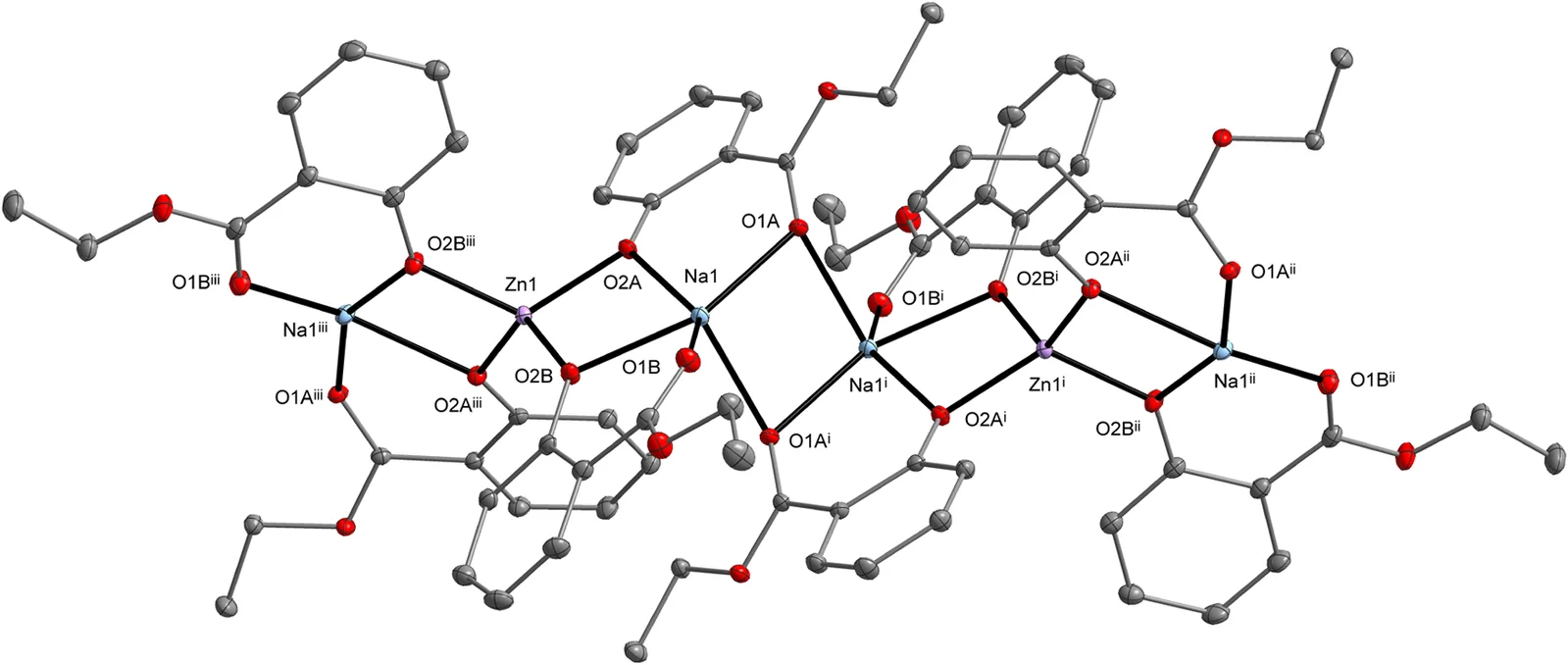
Molecular structure of [ZnNa_2_(sal-Et)_4_]_*n*_ (**24**). Displacement ellipsoids are drawn at the 25% probability level [symmetry code: (i) 1-*x*, 1-*y*, 1-*z*; (ii) *x*, 1-*y*, 0.5+*z*; (iii) 1-*x*, *y*, 0.5-*z*]. Hydrogen atoms are omitted for clarity.

Compounds **23**–**25** were further characterized by ^1^H and ^13^C NMR and FTIR-ATR spectroscopy (Figures S31–S39). ^1^H-DOSY NMR studies in THF-*d*_8_ suggested partial dimerization of compound **23** into hexanuclear species, whereas compounds **24** and **25** undergo dissociation of the coordination polymer framework to form trinuclear aggregates in solution (Figures S40–S42).

## Chemical Recycling of PET and PEICT

Alcoholysis reactions using *n*-BuOH as a reagent and homometallic and heterometallic aryloxides **1**–**25** as catalysts were investigated as a chemical recycling method for postconsumer PET and PEICT. Commercially available PET mineral water bottles and PEICT filter bottles were used as sources of aromatic polyesters. Typical reactions were conducted under solvothermal conditions at 120–240 °C for 2 h in 25 mL PTFE-lined hydrothermal reactors, employing compounds **1**–**25** as catalysts (Tables S4–S9). The reaction products were identified by FTIR-ATR spectroscopy, ESI-MS, and ^1^H and ^13^C NMR spectroscopy. The progress of alcoholysis was monitored over time by ^1^H NMR spectroscopy and quantified based on the amount of isolated, unreacted polymer residue.

Prior to catalytic studies, thermogravimetric analysis-differential scanning calorimetry (TGA-DSC) was used to assess the thermal stability of the polymers. PET and PEICT exhibited excellent thermal stability up to 360 °C, with mass losses not exceeding 4% (Figure 7). The melting temperature of PET, determined by DSC, was 248 °C, consistent with pristine PET. The onset degradation temperatures were 368 °C for PET and 351 °C for PEICT, while maximum degradation temperatures were observed at 423 and 409 °C, respectively. Thermal decomposition of PET resulted in approximately 11% char residue. It should be noted that crystalline domains are less susceptible to chemical degradation than amorphous regions; consequently, semicrystalline PET exhibits higher thermal and chemical stability than amorphous PEICT, which is relevant to the transesterification process.

**Figure 7:**
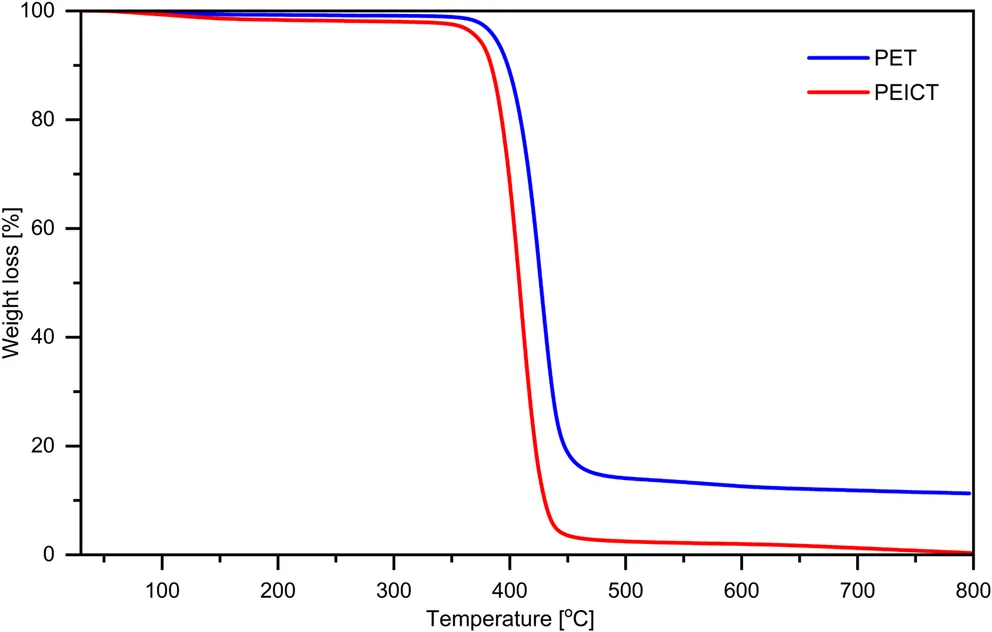
TGA thermograms of PET and PEICT investigated in alcoholysis reactions.

Initially, PET and PEICT alcoholysis were performed in the absence of a catalyst.

Under these conditions, only 2–11% PET (120–200 °C) and 2–9% PEICT (140–220 °C) were consumed (Tables S4 and S7). Subsequently, compound **1** was selected as a model catalyst for the degradation of both polymers. Reactions were conducted using reagent stoichiometry calculated relative to the terephthalic acid repeat unit (TA) in the polyester, with [TA_(PET/PEICT)_]/[*n*-BuOH]/[M] = 1/25/0.05. These reactions resulted in the formation of liquid organic product mixtures (Figure 8), achieving 99% conversion of PET and 73% conversion of PEICT at 160 °C (Tables S4 and S7).

**Figure 8:**
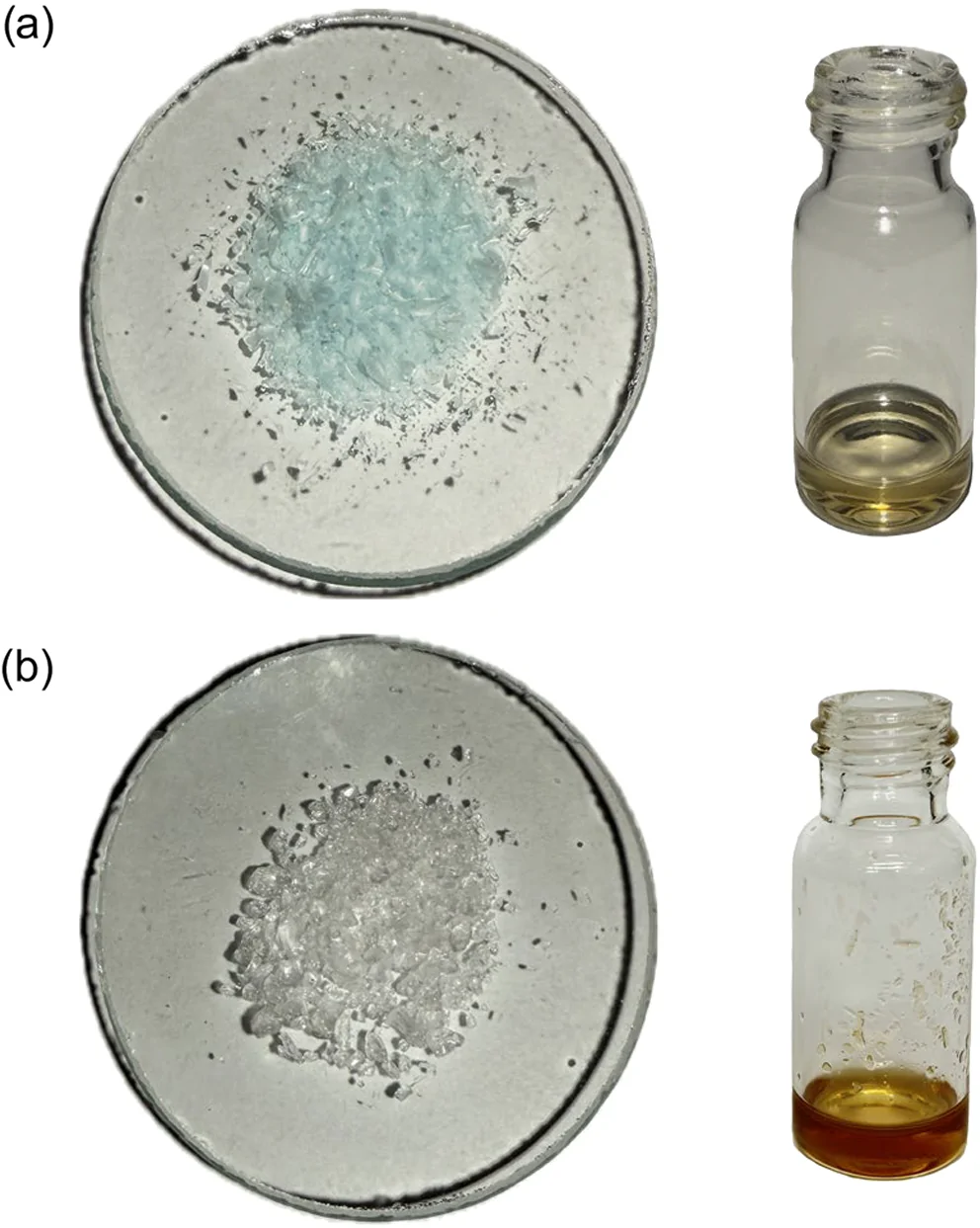
PET (a) and PEICT (b) waste used in the chemical recycling process and the corresponding liquid postreaction mixtures obtained after alcoholysis.

Comparison of FTIR-ATR spectra of PET/PEICT and their corresponding butanolysis products clearly demonstrates cleavage of ester linkages and provides insight into the chemical recycling pathway (for details, see Section 1 and Figures S43–S45). ^1^H, ^13^C, ^1^H–^1^H COSY, and ^1^H–^13^C HSQC NMR spectra of PET butanolysis reaction mixtures revealed DBT and EG as the sole detectable solution-phase products (Figure 9a). In contrast, PEICT butanolysis yielded DBT, EG, isosorbide, and both *cis*- and *trans*-isomers of 1,4-cyclohexanedimethanol (Figures 9b, S46, and S47).

**Figure 9:**
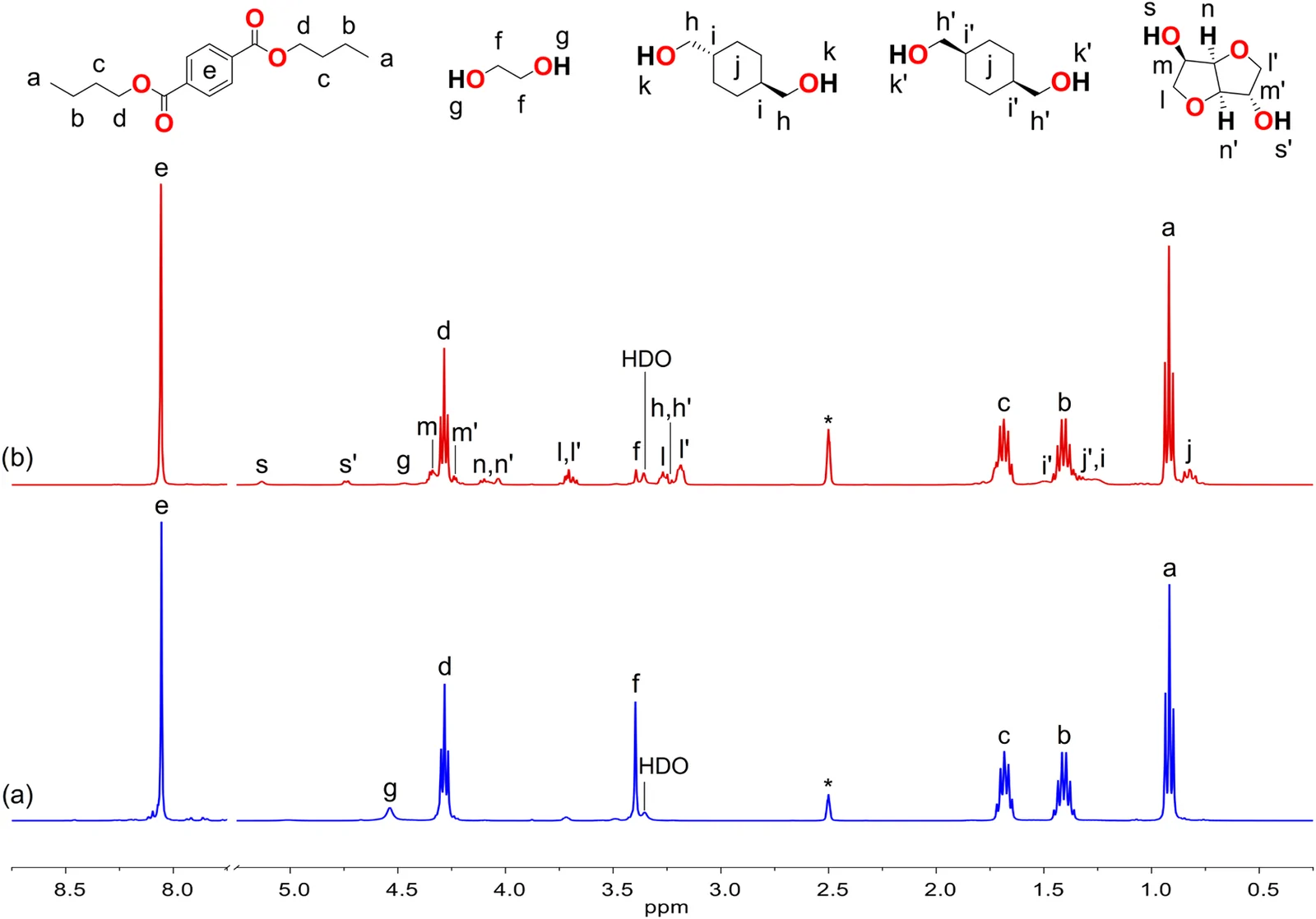
(a) ^1^H NMR spectrum of PET butanolysis reaction mixture using **1** as a catalyst after 2 h at 160 °C in DMSO-*d*_6_ and (b) ^1^H NMR spectrum of PEICT butanolysis reaction mixture using **1** as a catalyst after 2 h at 240 °C in DMSO-*d*_6_.

The ^1^H NMR spectrum of isolated DBT (Figure 10) shows aromatic resonances at 8.06 ppm, methylene signals at 4.29, 1.69, and 1.42 ppm, and terminal methyl resonances at 0.92 ppm. The ^13^C NMR spectrum showed characteristic resonances for C=O groups at 165.03 ppm, aromatic carbons at 133.72 and 129.44 ppm, and alkyl chain carbons at 64.86, 30.17, 18.72, and 13.58 ppm (Figure S48).

**Figure 10:**
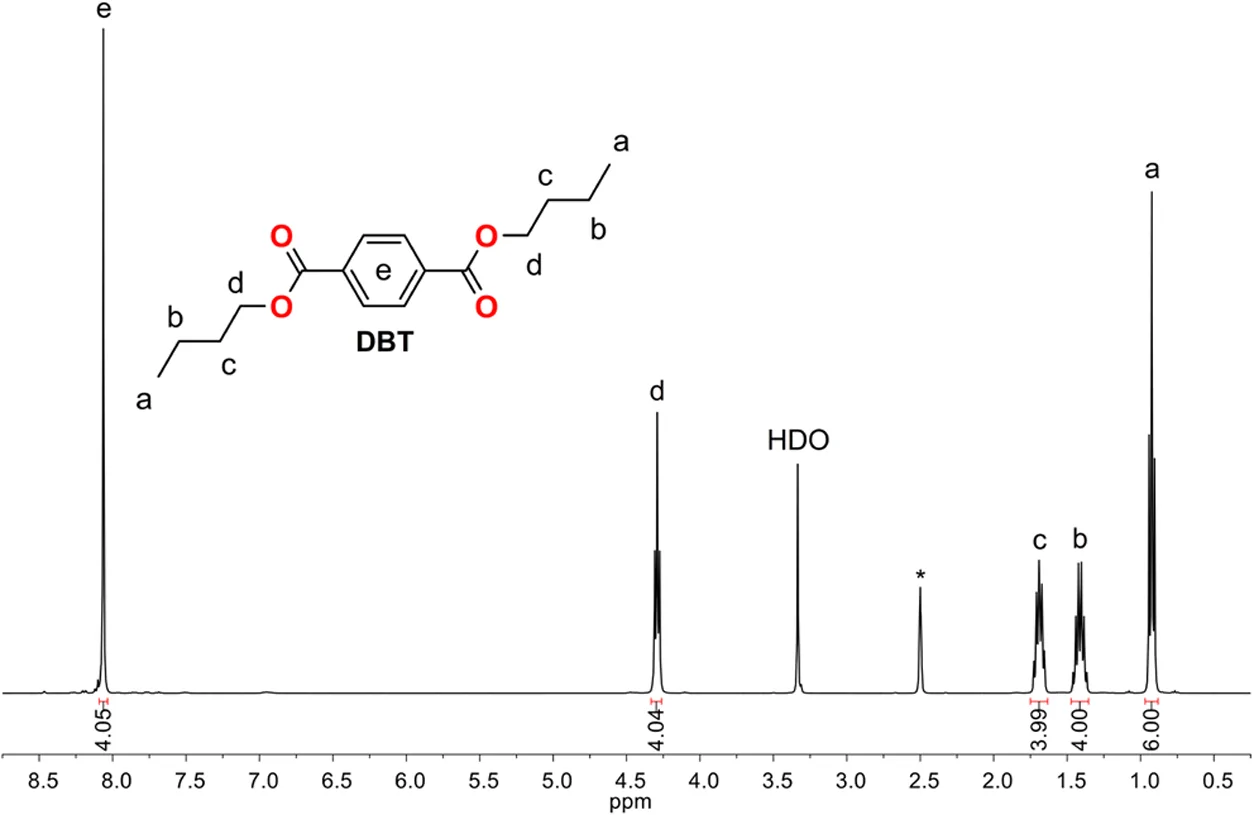
^1^H NMR spectrum of DBT in DMSO-*d*_6_.

ESI-MS analysis of the reaction mixtures provides deeper insight into the chemical processes occurring in solution during polyester transesterification and reveals the structures of intermediate products that are not detectable by ^1^H NMR spectroscopy (Figures 11 and 12). For PET degradation, the presence of 1,4-terephthalic acid (**B**), monobutyl terephthalate (**C**_**1**_), butyl vinyl terephthalate (**C**_**2**_), butyl 2-hydroxyethyl terephthalate (**C**_**3**_), butyl 2-(2-hydroxyethoxy)­ethyl terephthalate (**C**_**4**_), mono­(2-hydroxyethyl) terephthalate (**C**_**5**_), and butyl butyl 2-(4-carboxybenzoyloxy)­ethyl terephthalate (**D**_**1**_) was detected (Figures 11 and S49). For PEICT, intermediates **C**_**1**_–**C**_**4**_ were detected along with butyl (3S,3aR,6R,6aR)-hexahydro-3-hydroxyfuro­[3,2-*b*]­furan-6-yl terephthalate (**C**_**6**_); 2-hydroxyethyl-, 2-(butoxy)­ethyl-, and butyl (4-(hydroxymethyl)­cyclohexyl)­methyl terephthalates (**E**_**1**_–**E**_**3**_); bis­((4-(hydroxymethyl)­cyclohexyl)­methyl) terephthalate (**E**_**4**_); 1,4-cyclohexanediyl bis­(4-butoxycarbonylbenzoate) (**F**_**1**_); 1,4:3,6-dianhydrohexitol bis­(4-((1,4:3,6-dianhydrohexitol-2-yl)­oxycarbonyl)­benzoate) 4-(hydroxymethyl)­cyclohexylmethyl ester (**F**_**2**_); and butyl 4-(1,4-cyclohexanediyl)­bis­(4-oxobenzoyl) monoester (**F**_**3**_) (Figures 12 and S50).

**Figure 11:**
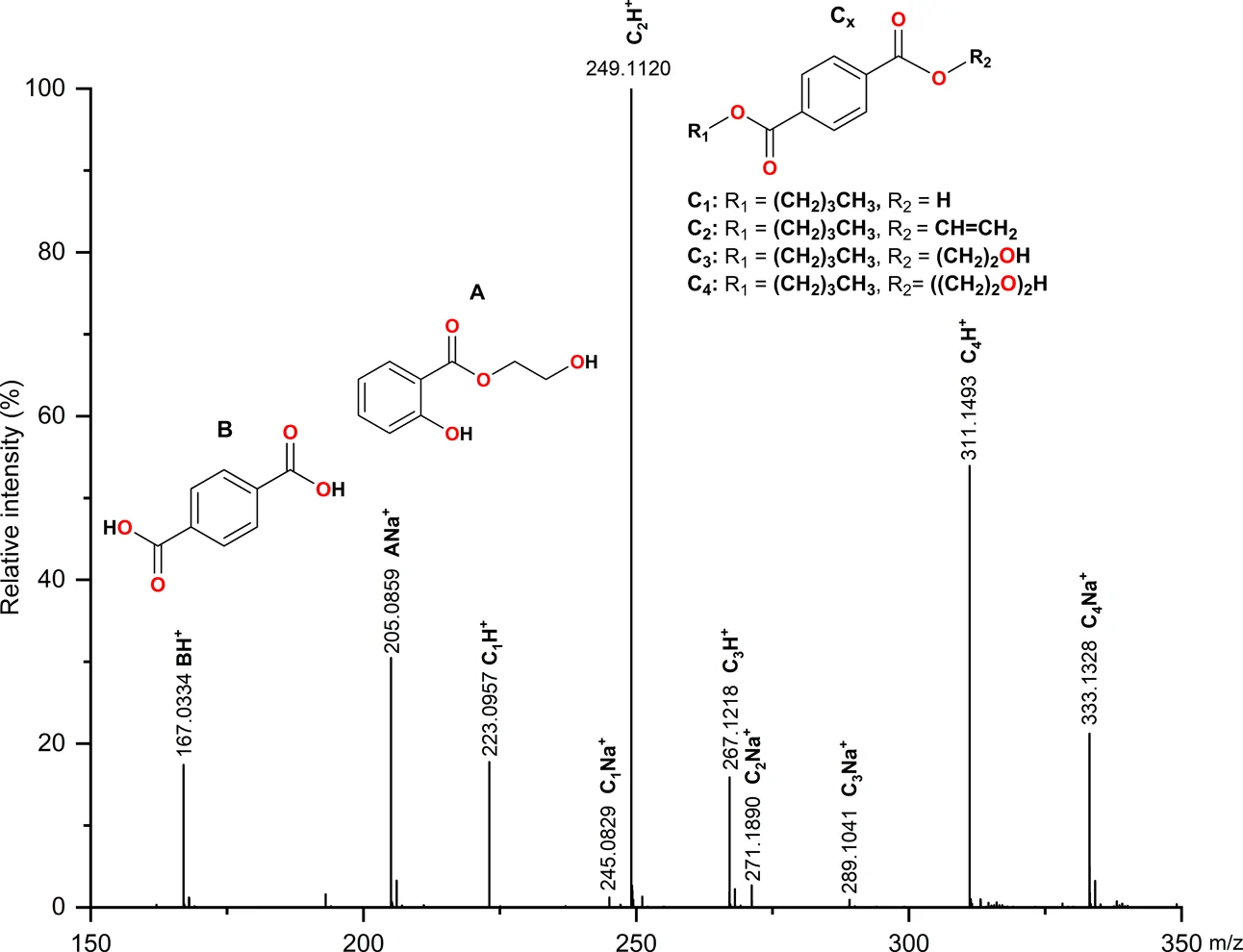
ESI-MS spectrum of intermediate products identified in the reaction mixture after PET butanolysis.

**Figure 12:**
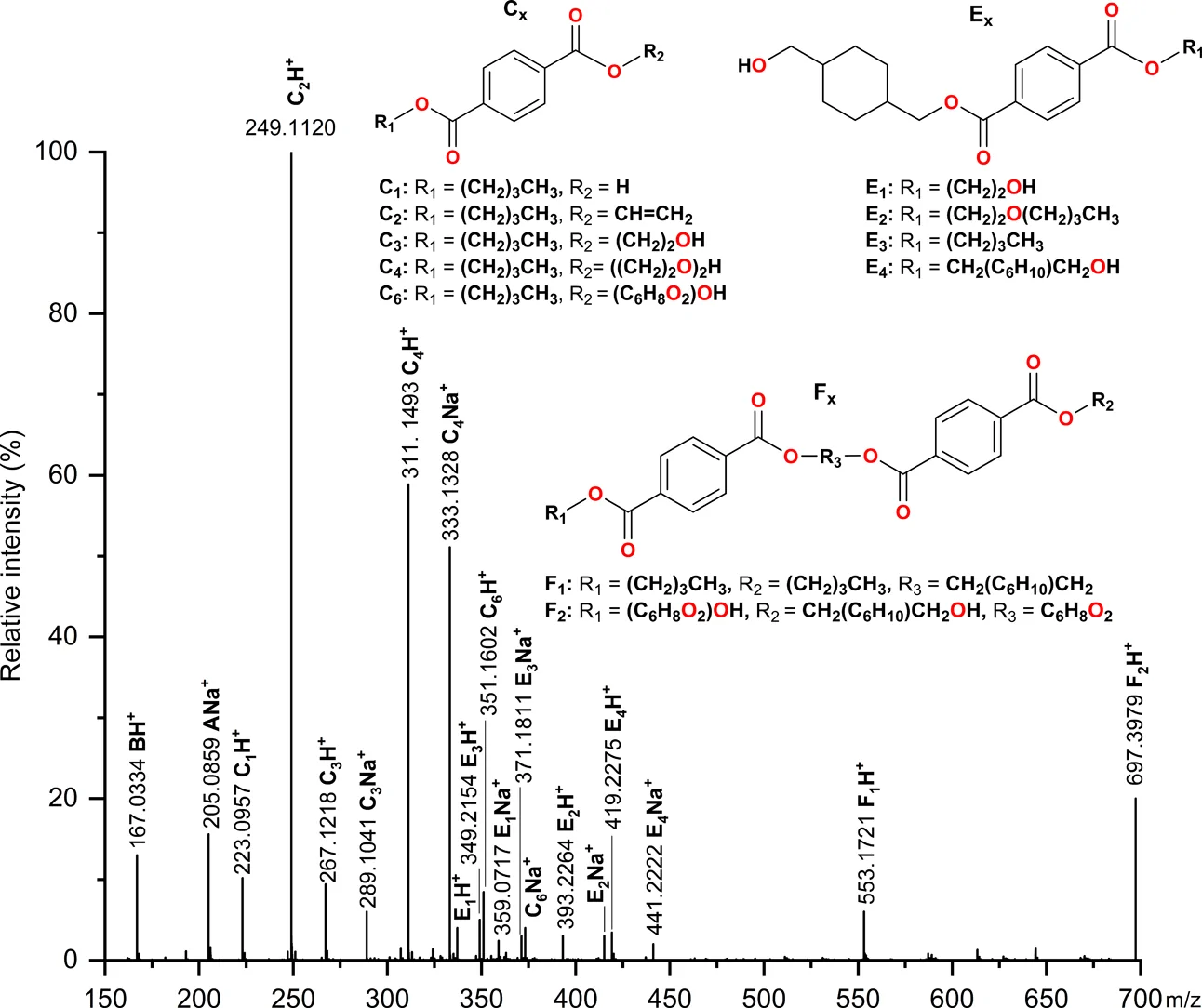
ESI-MS spectrum of intermediate products identified in the reaction mixture after PEICT butanolysis.

Formation of **C**_**1**_ and **C**_**2**_ results from thermal cleavage of PET ester bonds, generating chains terminated by acid and vinyl ester groups,^94^ while the 2-(2-hydroxyethoxy)­ethyl substituent in **C**_**4**_ arises from ethoxylation of ethylene glycol.

During PET alcoholysis catalyzed by compound **1**, catalyst residues precipitated as insoluble crystalline solids after the excess *n*-BuOH was removed by distillation (Figures S51 and S52). Single-crystal X-ray diffraction, NMR, and FTIR-ATR spectroscopy identified this material as the two-dimensional lithium coordination polymer [Li­(BTA)]_*n*_ (**26**, 12%) (Figures S53–S55). In the molecular structure of **26**, the Li^+^ ions are coordinated by 4-(butoxycarbonyl)­benzoate anions (BTA^–^), forming distorted tetrahedral geometries defined by four carboxylate oxygen atoms from four different BTA^–^ ligands (Figure 13, Table S3).

**Figure 13:**
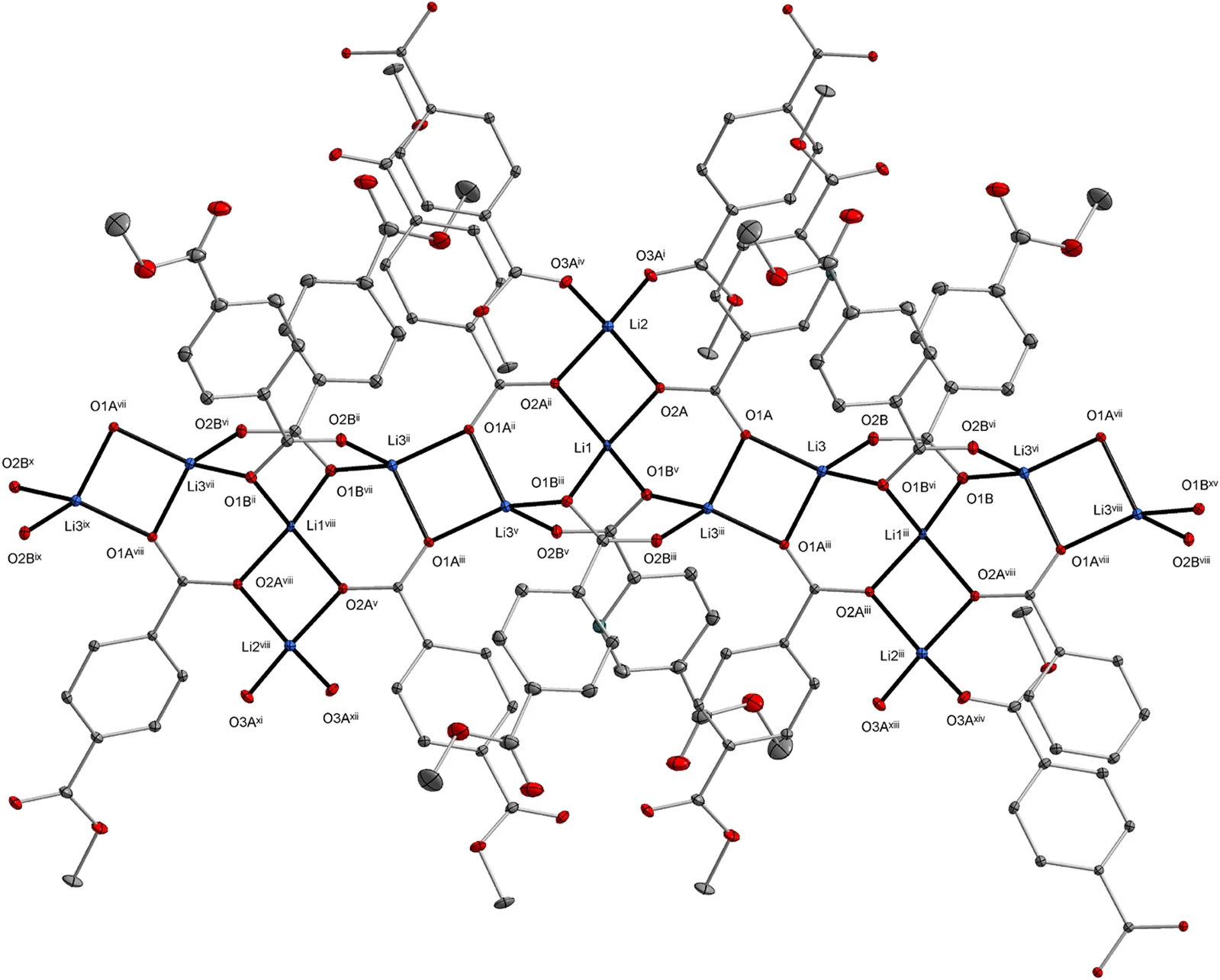
Molecular structure of [Li­(BTA)]_*n*_ (**26**). Displacement ellipsoids are drawn at the 25% probability level [symmetry code: (i) 1-*x*, 1-*y*, 1-*z*; (ii) 0.5-*x*, *y*, 1-*z*; (iii) 1-*x*, 2-*y*, 1-*z*; (iv) −0.5+*x*, 1-*y*, *z*; (v) −0.5+*x*, 2-*y*, *z*; (vi) 1.5-*x*, *y*, 1-*z*; (vii) −1+*x*, *y*, *z*; (viii) 0.5+*x*, 2-*y*, *z*; (ix) -*x*, 2-*y*, 1-*z*; (x) −1.5+*x*, 2-*y*, *z*; (xi) −1+*x*, 1+*y*, *z*; (xii) 0.5-*x*, 1+*y*, 1-*z*; (xiii) *x*, 1+*y*, *z*; (xiv) 1.5-*x*, 1+*y*, 1-*z*; (xv) 2-*x*, 2-*y*, 1-*z*]. Carbon atoms of *n*-butyl groups, as well as hydrogen atoms of alkyl and aryl groups, are omitted for clarity.

The oxygen atoms of the carboxylate and ester groups coordinate to two Li^+^ ions (O1A, O2A, and O1B), to one Li^+^ ion (O2B and O3A), or are not involved in coordination (O3B, O4A, and O4B). Coordination of the ester oxygen atom O3A to Li2 links adjacent one-dimensional zigzag chains, with Li···Li separations of 8.428 Å, resulting in the formation of a two-dimensional network. The BTA^–^ anions exhibit significant deviations from planarity, with the carboxyl groups rotated out of the aromatic ring plane by 27.48° (ligand A) and 13.99° (ligand B). ^1^H-DOSY NMR analysis of **26** in THF-*d*_8_ confirmed solvent-induced disruption of the polymeric coordination network, leading to the formation of nona- or decanuclear aggregates in solution (Figure S56). Notably, only three examples have been reported to date in which terephthalic acid generated by hydrolysis of PET or PBT in the presence of polyoxometalates Ni_6_PW_9_, Zn_4_PMo_12_, or V_6_S acts as a ligand in metal–organic frameworks (Ni-POMOF, Z-POMOF, and VMOP-11).^95^

Further isolation of catalyst **1** residues from PET butanolysis performed at a reactant stoichiometry of [TA­(PET)/*n*-BuOH/Li] = 1/25/0.10 at 160 °C for 2 h, involving distillation of the alcohol followed by precipitation of the inorganic compounds with hexane, revealed the presence of three lithium-containing species in solution. The first species was identified as the hexanuclear compound [Li_6_(sal-Bu)_6_] (MW = 1148 g/mol, *r*_H_ = 8.99 Å), the second as [Li_6_(sal-Bu)_6_(DBT)] (MW = 1353 g/mol, *r*_H_ = 9.49 Å), while the third was assigned to [Li­(BTA)]_7_ (MW = 1562 g/mol, *r*_H_ = 9.96 Å) (Figure S57). Interestingly, the catalyst residues isolated from reactions employing complexes **9**, **11**, or **25** contain heterometallic compounds coordinated by sal-Bu ligands. However, these species exhibit significantly lower solubility in THF-*d*_8_ and a higher degree of aggregation in solution compared to the starting sal-Me derivatives (Figure S58).

The solid-state structure of **26** provides insight into potential metal-binding environments in PET and serves as a molecular model for understanding the formation of reaction intermediates. The structural data confirm that, during PET alcoholysis, compound **26** is formed via the reaction of **1** with monobutyl terephthalate (BTAH). The presence of BTAH is associated with the carboxyl end groups present in PET, as the polymer used in this study was derived from postconsumer waste material. Formation of the lithium carboxylate **26** is particularly evident after evaporation of excess *n*-BuOH from the reaction mixture, followed by exposure of the remaining liquids to atmospheric moisture for 24–48 h. To verify this hypothesis, an independent reaction between TAH_2_ and compound **1** (1:1) was carried out in *n*-BuOH at 180 °C for 3 h. PXRD analysis confirmed the formation of **26** and [Li_2_(TA)]_*n*_,^96^ as well as the presence of excess unreacted organic acid (Figure S59). When DBT was used in place of TAH_2_, no reaction between the ester and **1** was observed, aside from esterification of the Me group on the Bu in the ligand (Figures S60 and S61). The model, ^1^H-DOSY NMR analysis of a mixture of **1**, DBT, and MeOH (0.033:1:4), revealed coordination of the ester to **1**, without degradation of the hexanuclear structure in solution (Figure S62).

The general pathway for the recovery of phthalates from postconsumer PET or PEICT waste in the form of DBT, as established by the above structural, spectroscopic, and analytical studies, is summarized in Scheme 4.

**Scheme 4:**
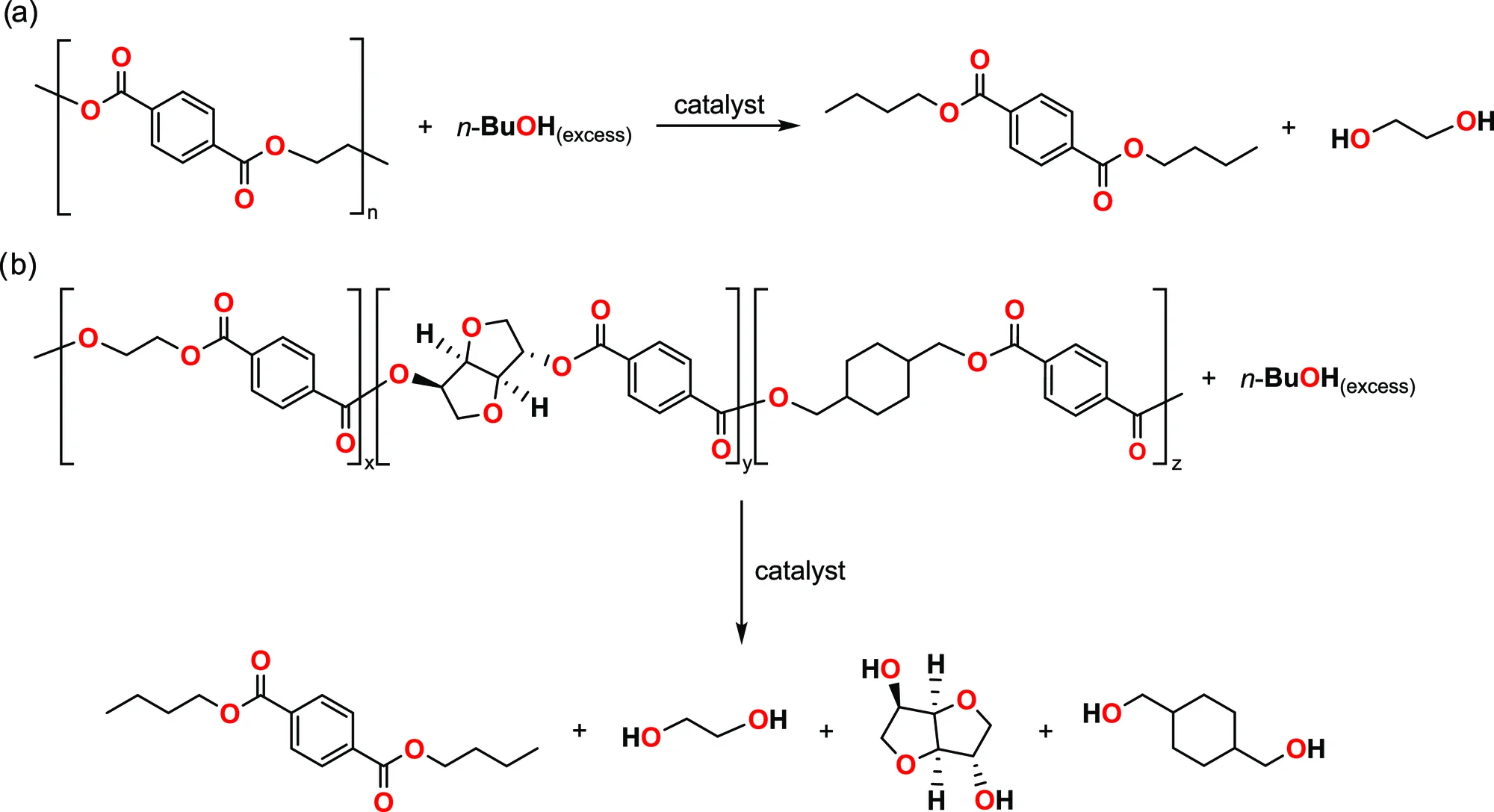
General Route for the Recovery of Phthalates from Postconsumer PET or PEICT Waste in the Form of DBT

Once a suitable system for PET/PEICT alcoholysis had been identified, we investigated homo- and heterometallic aryloxides **1**–**25** as catalysts for efficient depolymerization under mild reaction conditions, with particular emphasis on low reaction temperatures (Tables S4–S9). We began by screening the catalytic activities of homometallic aryloxides. In the PET transesterification reaction using *n*-BuOH at 160 °C, the catalytic activity of alkali metal aryloxides decreased in the order Li^+^ (99%) **≫** Na^+^ (47%) > K^+^ (40%) > Rb^+^ **≈** Cs^+^ (16–19%) (Table S4). These activities were nevertheless significantly higher than those of divalent metal aryloxides at 170 °C, which followed the trend Mg^2+^ (74%) > Ca^2+^ (51%) > Zn^2+^ (38%). When the reaction temperature was increased to 180 °C, the same trend was maintained for divalent metal catalysts, with Mg^2+^ (99%) > Ca^2+^ (75%) > Zn^2+^ (64%); under these conditions, PET conversion exceeded that obtained with heavier alkali metal aryloxides, which followed the order Na^+^ (58%) > K^+^ (57%) > Cs^+^ (41%) > Rb^+^ (25%) (Table S4). The significantly higher efficiency of the hexanuclear lithium compound **1**, which features multiple adjacent active sites, compared with divalent aryloxides of lower nuclearity and fewer cooperative binding sites, suggests an aggregate-based alcoholysis mechanism in which the metal aryloxides polarize and activate the PET carbonyl group toward nucleophilic attack by *n*-BuOH.

Catalyst **1** exhibits a high charge density and contains small-radius Li^+^ ions, which effectively increase the electrophilicity of the coordinated PET carbonyl group, thereby lowering the activation barrier for nucleophilic attack by ROH. Li–O interactions are sufficiently ionic and dynamic to enable rapid formation and release of reaction intermediates, resulting in high catalytic efficiency. In addition, lithium aryloxides are generally more soluble and more labile than their heavier alkali metal analogues, allowing more effective penetration of the polyester surface. For alkali metal aryloxides **2**–**5**, the increasing ionic radius and concomitant decrease in charge density lead to reduced Lewis acidity, diminished polarization and activation of the ester C=O group, and weaker stabilization of the transition state. Heavier alkali metal ions exhibit more diffuse and weaker interactions, making the balance between substrate activation and ligand lability less favorable. Furthermore, metal ions with larger ionic radii may be less accessible due to higher coordination numbers or partial immobilization within the polymer matrix, further reducing catalytic effectiveness.

Divalent metal aryloxides **6**–**8** exhibit higher intrinsic Lewis acidity and would therefore be expected to promote stronger carbonyl activation; however, they are less efficient than **1**. The Mg^2+^, Ca^2+^, and Zn^2+^ ions interact more strongly with ester groups than M′ cations, stabilizing metal–substrate complexes but limiting fast substrate exchange and product release, which ultimately reduces catalytic performance. Among these, Mg^2+^ is a relatively small cation with strong Lewis acidity and comparatively fast ligand-exchange kinetics, which rationalizes its higher activity relative to Ca^2+^. In contrast, Zn^2+^ forms more covalent, thermodynamically stable, and kinetically inert complexes with organic ligands, including polymer backbones, which diminishes catalytic activity. Structurally, compound **6** is dimeric, whereas **7** and **8** form tetra- and trinuclear aggregates with less favorable lability for efficient catalysis.

The catalytic activity of compounds **1**–**8** was compared with that of the corresponding acetates, carbonates, hydroxides, and methoxides (Table S10). Among the tested inorganic compounds, LiOAc and LiOMe were the most active, achieving PET conversions of 23 and 19% at 150 °C, respectively. However, these values were still substantially lower than the 77% conversion obtained with compound **1**.

Motivated by recent reports demonstrating enhanced activity or selectivity via heterometallic cooperativity,^83,84,97,98^ we examined heterometallic aryloxides **9**–**25** and compared their performance with that of homometallic catalysts **1**–**8**. Comparison of reactions carried out at 160 °C showed that the reactivity of nearly all heterometallic complexes (with the exception of **12** and **13**) is significantly higher than that of their homometallic counterparts (Tables S4–S6). The resulting PET conversion values, with only a few exceptions (**10**, **12**, **13**, and **17**), fall in the range of 92–100% (Tables S5 and S6). Among the tested compounds, the highest activity under the mildest reaction conditions (140 °C) was observed for compounds **25** (79%), **15** (76%), **20** (67%), **23** (64%), **16** (55%), and **24** (48%), as shown in Figure 14. Increasing the reaction temperature to 150 °C led to a new reactivity trend, with compound **20** being the most active (99%), followed by **16** (93%), **25** (92%), **23** (90%), **18** (87%), **24** (83%), **9** (81%), and **15** (77%) (Tables S5 and S6).

**Figure 14:**
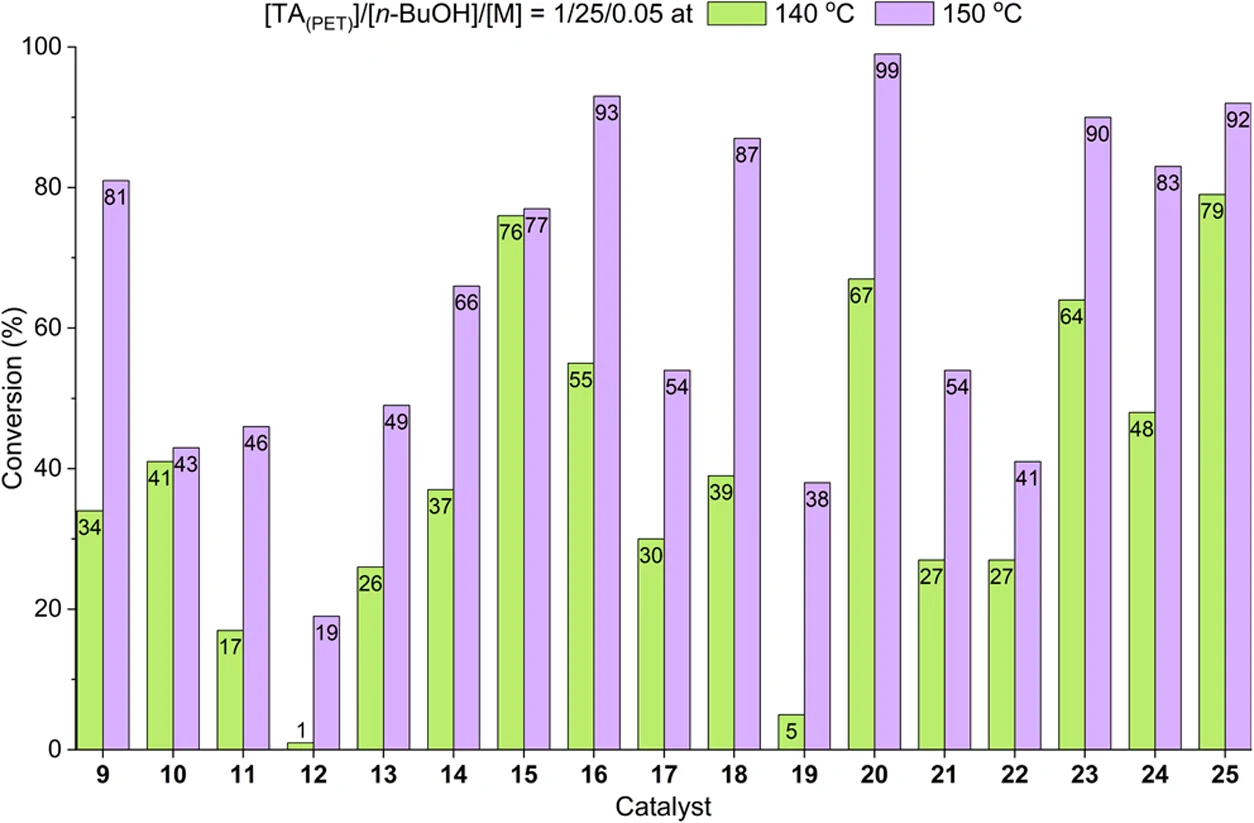
Catalytic activities of **9**–**25** in PET alcoholysis with a stoichiometry of [TA_(PET)_]/[*n*-BuOH]/[M] = 1/25/0.05 at 140 °C (green) or 150 °C (violet) for 2 h.

From a structural chemistry perspective, within the group of heterometallic tetranuclear catalysts **9**–**18**, compounds based on Ca^2+^–K^+^, Mg^2+^–Li^+^, Zn^2+^–K^+^, and Zn^2+^–Na^+^ combinations exhibit significantly higher catalytic activity (66–93%) than catalysts containing Ca^2+^–Na^+^, Zn^2+^–Li^+^, Mg^2+^–K^+^, and Mg^2+^–Na^+^ pairs (43–54%). In this context, the highest activity is observed for catalysts sharing the same structural motif (**9**, **16**, **18**), featuring external M′^+^ ions and internal M^2+^ ions.

The next most active catalysts are those with a reversed metal arrangement in the central core, containing sterically accessible, pentacoordinated Zn^2+^ ions located at the outer edges of the dicubane units (**14** and **15**). Subsequent spectroscopic studies showed that zinc complexes **13**–**15**, unlike the tetranuclear Mg^2+^/Ca^2+^–M′^+^ systems, do not retain their solid-state structures in solution and instead dissociate into dinuclear or trinuclear species. This behavior likely enhances their catalytic performance by reducing steric crowding and increasing solution mobility.

The least catalytically favorable systems are those containing six-coordinate Mg^2+^ ions chelated by three sal-Me ligands at the outer edges of the central core (**10** and **11**), where coordination crowding hampers effective C=O activation. In contrast, in complexes **16**–**18**, the presence of six- or even seven-coordinate Ca^2+^ ions facilitates participation in the polarization of the PET carbonyl group. The high reactivity of catalyst **20**, which is based on a vertex-sharing {CaLi_3_O_4_}_2_ heterodicubane motif, can be rationalized in a manner analogous to that of catalyst **1**, with the additional stabilization of structural integrity provided by the Ca^2+^ ion.

The high catalytic activity of the trinuclear complexes **23**–**25** can be attributed to the presence of sterically unhindered, four-coordinate Zn^2+^ centers combined with two external M′^+^ ions, which confer greater solubility in *n*-BuOH and enhanced mobility compared with complexes **14** and **15**. At 160 °C, compounds **24** and **25** exhibit markedly enhanced activities (94–100%) relative to homometallic Zn^2+^ (11%) or Na^+^/K^+^ aryloxides (47%/40%), highlighting the crucial cooperative role of Zn^2+^–Na^+^/K^+^ ion pairs in PET transesterification. Moreover, the results obtained with catalysts **16**, **20**, and **25** at 150 °C after 2 h are markedly superior to the 92% yield of DBT reported for PET alcoholysis with *n*-BuOH using 4-sulfonic acid butyltripropylammonium ferric bromide, which required harsher conditions (190 °C, 6 h, PET/*n*-BuOH/Fe = 1/2.43/0.03).^99^ The use of calcium-based compounds as catalysts for the recycling of aromatic polyesters can be viewed as a return to the pioneering DuPont patent from 1954, which described PET methanolysis using CaH_2_ as a precatalyst.^100^

We subsequently examined the stability of catalysts **20** and **25** over multiple depolymerization cycles. When catalyst **20** was used at 150 °C for 2 h, the PET conversion decreased to 42 and 32% in the second and third cycles, respectively. In contrast, when catalyst **25** was tested at 180 °C for 2 h, complete PET conversion was achieved over three consecutive cycles. In the fourth and fifth cycles, however, the conversion dropped to 40 and 26%, respectively. Upon addition of fresh catalyst portions, the subsequent six PET batches were converted with efficiencies of 97–99%. After the twelfth cycle, the reaction mixture developed a gel-like consistency due to the formation of a mixture of DBT, ethylene glycol terephthalate oligomers, and TAH_2_ (DBT/oligomers/TAH_2_ = 59:40:1%). The results obtained for compound **20**, together with the molecular structure of **26**, indicate that Li^+^ ions have a strong tendency to form thermodynamically stable lithium terephthalates, which are significantly less reactive than lithium aryloxides such as **1** or **20**. In contrast, catalyst **25**, containing Zn^2+^–K^+^ ion pairs, is considerably less susceptible to deactivation, and doubling its loading enables the efficient conversion of an additional six PET batches.

In order to verify the catalytic activity of the selected catalysts toward PET waste from different sources, PET butanolysis reactions were performed using waste bottles obtained from four different manufacturers. These samples differed in color and origin from the PET materials previously used in this study (Figure S63). The obtained results correlate well with the data presented above, with the maximum difference in reactivity not exceeding 11% (Table S11).

Finally, the activity of catalyst **25** was evaluated using a mixed plastic waste stream composed of PET, polystyrene (PS), high-molecular-weight polyethylene (HDPE), and high-consistency silicone rubber (HCR), each present at 25 wt %. After 3 h of reaction at 160 °C, PET conversion was close to quantitative (Figure S64).

PEICT alcoholysis was conducted using lower catalyst loadings ([TA_(PEICT)_]/[*n*-BuOH]/[M] = 1/25/0.025) at temperatures ranging from 140 to 200 °C. The catalytic activity of alkali metal aryloxides **1**–**5** was very low, even at 200 °C, affording PEICT conversion values of only 30–40% (Table S7). In contrast, Mg^2+^-, Zn^2+^-, and Ca^2+^-based aryloxides **6**–**8** achieved 90–100% polyester conversion under the same conditions (Table S7). This pronounced difference in the reactivity of group 1 catalysts is mainly attributed to the presence of 1,4-cyclohexanedimethanol and isosorbide in the reaction mixture, which reduce the activity of alkali metal aryloxides by limiting their solubility or by blocking key coordination sites. In general, the catalytic activity of all heterometallic species in PEICT transesterification is significantly higher than that of their homometallic counterparts (with the exception of **12**; Tables S7–S9). The highest PEICT conversion efficiencies were obtained in the presence of Zn^2+^-based catalysts **13**, **15**, and **23**–**25** (72–98%) and Ca^2+^-containing compounds **20**–**22** (63–77%) (Tables S8 and S9). The origins of the exceptionally high catalytic activity of compounds **15**, **23**–**25**, and **20** were discussed above in the context of PET chemical recycling. The activity of the pentanuclear compounds **21** and **22** is particularly pronounced at elevated temperatures (e.g., 160 °C) in both PET and PEICT depolymerization. Among the heterometallic double-opened tetranuclear catalysts **9**–**18**, Zn^2+^–M′^+^ complexes (57–98%) exhibited the highest activity, whereas Mg^2+^–M′^+^ and Ca^2+^–M′^+^ dicubanes showed comparable but lower activities (45–59%) (Figure 15). In this context, the superior performance of catalysts **13**–**15** and **23**–**25** can be attributed to their dissociation in solution and the presence of sterically uncrowded, tetra- or pentacoordinated Zn^2+^ ions in the catalytically active species. Notably, the aforementioned calcium-based catalysts (**16** and **20**–**22**) also ranked among the most active systems under the mildest conditions (140 °C), affording 46–66% DBT (Figure 15).

**Figure 15:**
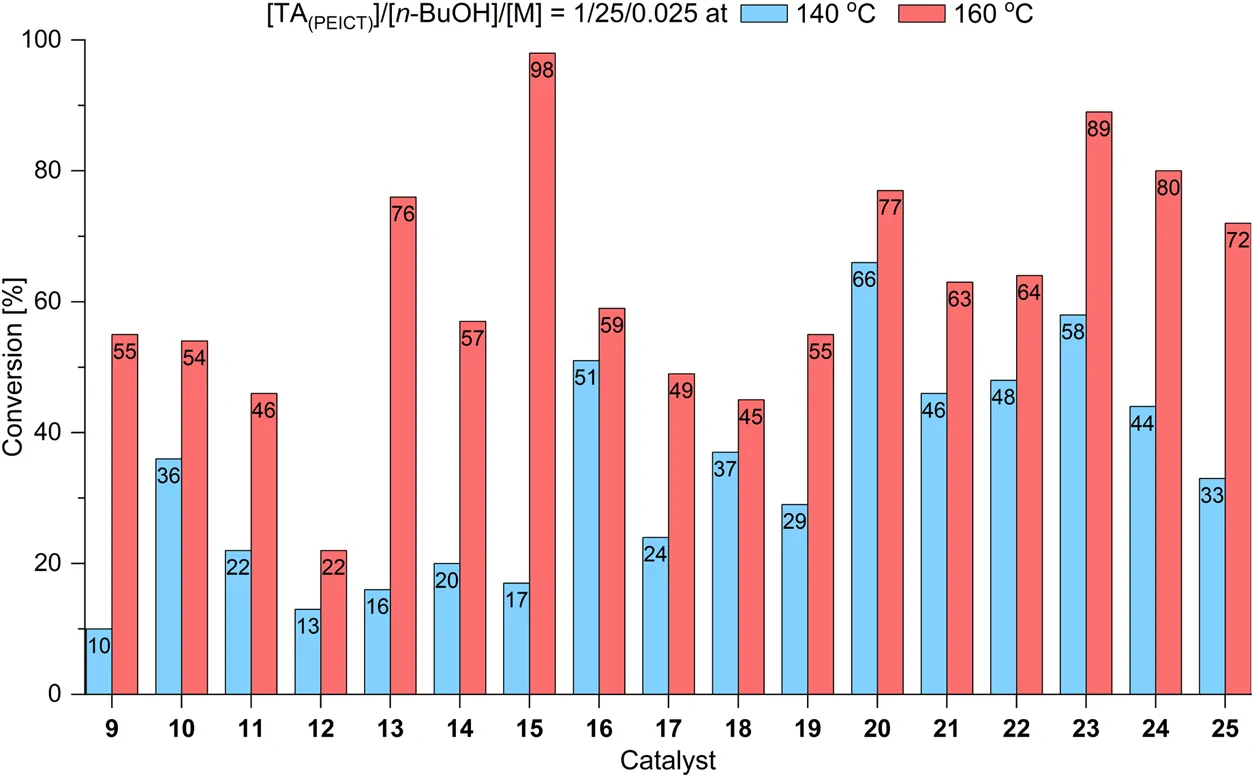
Catalytic activities of **9**–**25** in PEICT alcoholysis with a stoichiometry of [TA_(PEICT)_]/[*n*-BuOH]/[M] = 1/25/0.025 at 140 °C (light blue) or 160 °C (red) for 2 h.

SEM analysis of virgin PET and PEICT powders clearly demonstrated very flat, smooth surfaces on irregularly shaped particles (Figures 16a–d and 17a–d). After the alcoholysis reaction, the unreacted aromatic polyester materials underwent pronounced morphological changes induced by thermal stress and chemical interaction with the alcohol. As a result of chemical degradation, the materials became more brittle, developed cracks, and exhibited surface depressions and creases due to the progressive loss of structural integrity, ultimately fragmenting into smaller particles (Figures 16e-h and 17e-h).

**Figure 16:**
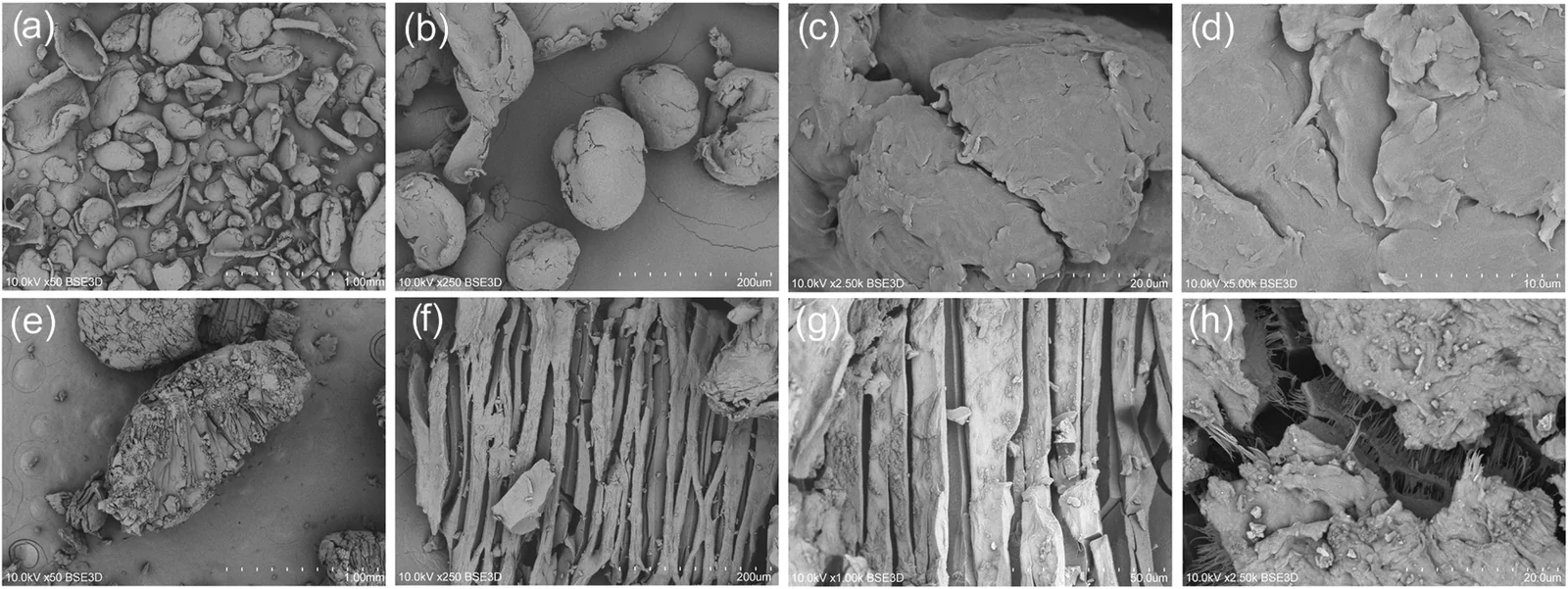
Comparison of SEM micrographs of virgin PET (a–d) and solid residues isolated from the reaction mixture after alcoholysis (e–h).

**Figure 17:**
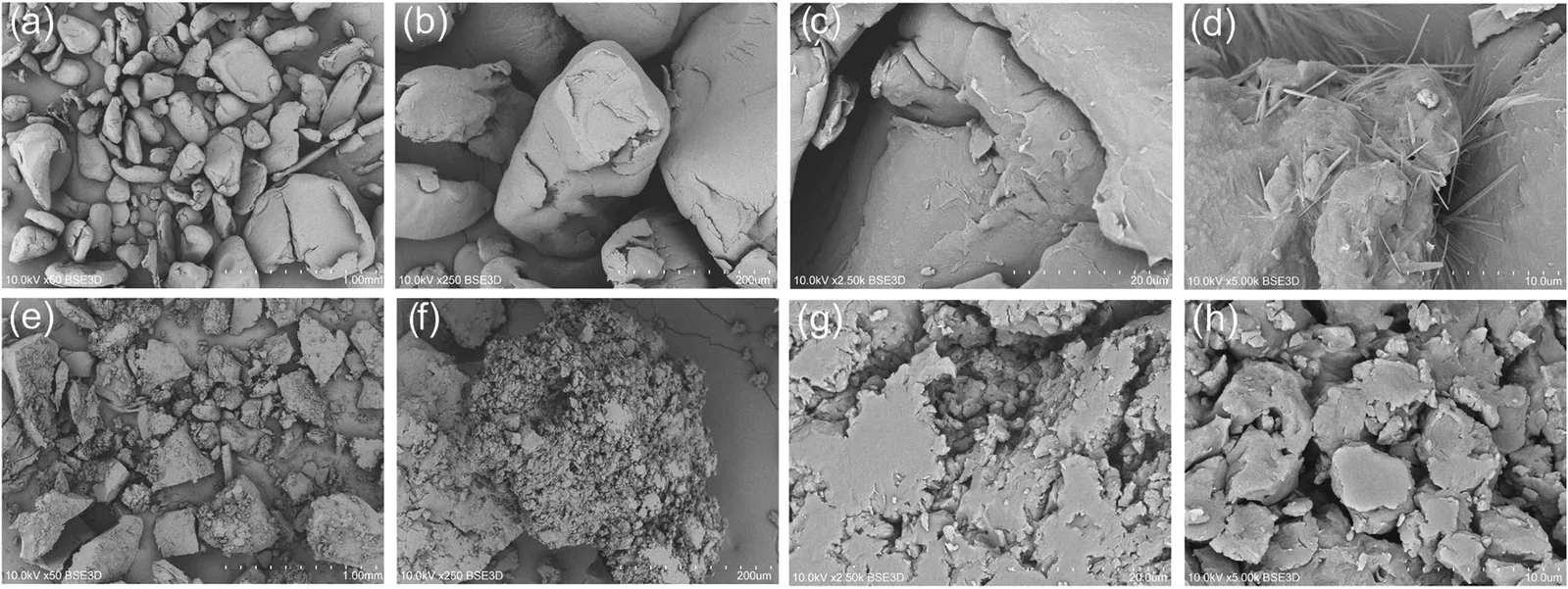
Comparison of SEM micrographs of PEICT (a–d) and solid residues isolated from the reaction mixture after alcoholysis (e–h).

In the initial stages of the process, *n*-BuOH diffuses into the polymer matrix, causing polymer swelling and thereby increasing the diffusion rates. The resulting surface degradation increases the porosity of the polymeric resins and enhances mass transport of the *n*-BuOH-containing catalysts into deeper layers of the material. Nucleophilic attack by the lone electron pairs of ROH on the ester carbonyl carbon (C=O) of PET/PEICT leads to the formation of a new C–O bond with *n*-BuOH and concomitant cleavage of the ester C–O bond.

## Conclusions

In this work, a comprehensive series of homo- and heterometallic alkali- and alkaline earth/transition-metal aryloxides were synthesized, structurally characterized, and systematically evaluated as catalysts for the solvothermal alcoholysis of postconsumer PET and PEICT wastes. The results clearly demonstrate that metal identity, nuclearity, aggregation state, and solution behavior play decisive roles in determining catalytic efficiency under mild depolymerization conditions. Among the homometallic catalysts, the hexanuclear lithium aryloxide **1** exhibited outstanding activity in PET butanolysis, achieving near-quantitative conversion at 160 °C. In contrast, heavier alkali metal aryloxides showed significantly lower activity, following the trend Li^+^ (99%) **≫** Na^+^ (47%) > K^+^ (40%) > Rb^+^ **≈** Cs^+^ (16–19%), which is attributed to decreased Lewis acidity, weaker activation of the ester carbonyl group, and reduced accessibility of catalytically active sites.

Homometallic Mg^2+^, Ca^2+^, and Zn^2+^ aryloxides, despite their higher Lewis acidity, displayed lower activity below 180 °C, with PET conversion following the order Mg^2+^ (74%) > Ca^2+^ (51%) > Zn^2+^ (38%) at 170 °C. However, the incorporation of alkali metal cations into divalent metal aggregates significantly enhances the catalytic performance. In particular, Zn^2+^–M′^+^ and Ca^2+^–M′^+^ heterometallic systems proved highly effective, with several compounds achieving high PET and PEICT conversion even at temperatures as low as 140–160 °C. Among all catalysts tested, the highest activity in PET butanolysis under mild conditions (150 °C) was observed for compounds **20** (99%), **16** (93%), **25** (92%), and **23** (90%).

In PEICT alcoholysis at 160 °C, the presence of bulky diol components, such as isosorbide and 1,4-cyclohexanedimethanol, significantly reduced the activity of alkali metal aryloxides, likely due to solubility limitations and coordination-site blocking. Under these conditions, divalent and heterometallic catalysts—especially Zn^2+^-based systems **13**, **15**, and **23**–**25** (72–98%) outperformed alkali metal analogues, highlighting the importance of adaptable coordination environments and cooperative metal effects.

The markedly higher efficiency of multinuclear, heterometallic compounds points to an aggregate-based mechanism, in which multiple adjacent metal centers cooperatively activate the polymer chain toward nucleophilic attack by alcohol. SEM analysis confirmed progressive surface degradation of PET and PEICT during alcoholysis, with increased porosity and fragmentation facilitating mass transfer of both alcohol and the catalyst into the polymer matrix.

Importantly, recovery of terephthalate as DBT represents an economically attractive route for the chemical recycling of mixed and unsorted aromatic polyester waste streams. The isolation and structural characterization of postcatalytic lithium terephthalate residues, [Li­(BTA)]_*n*_ (**26**), further provide insight into catalyst deactivation pathways and metal–polymer interactions.

Overall, this study establishes clear structure–activity relationships for metal aryloxide catalysts in polyester alcoholysis and demonstrates that rational catalyst design combining appropriate metal ions, nuclearity, and solution lability enables efficient depolymerization of PET and PEICT under mild conditions. These findings offer valuable guidance for the development of further catalytic systems for the sustainable chemical recycling of aromatic polyesters.

## Experimental Section

### Materials and Methods

All syntheses were performed under a dry nitrogen atmosphere using standard Schlenk techniques. Solvents were purified using conventional methods: toluene, hexane, and THF were distilled over sodium; ethanol and methanol were distilled over magnesium; and dichloromethane was distilled over P_2_O_5_. All chemical reagents were purchased from commercial sources: methyl salicylate, dibutylmagnesium, diethylzinc, metallic calcium, lithium, sodium, potassium, rubidium, and cesium (Sigma-Aldrich); ethanol (Stanlab); methanol, *n*-butanol, dichloromethane, and hexane (Chempur); THF and toluene (Eurochem BDG); THF-d_8_ (Eurisotop); and DMSO-d_6_ (Deutero and Eurisotop). PET was obtained from commercially available mineral water bottles (Cisowianka), and PEICT was obtained from a drinking bottle distributed by Mid-Ocean Brand BV. For PET alcoholysis, analytical-grade (p.a.) n-butanol was used as received without further purification (the minimum n-BuOH content was 99.5%, while the maximum contents of acidic impurities, butyl aldehydes and ketones, and water were 0.0025, 0.03, and 0.1%, respectively).

### Caution!

Di-*n*-butylmagnesium and diethylzinc are highly pyrophoric and react violently with air and moisture. They must be handled exclusively under an inert atmosphere in a glovebox or by using appropriate Schlenk techniques in a well-ventilated fume hood. When using needles and syringes, locking fittings, thoroughly dried glassware, and a positive-pressure inert gas supply should always be employed. Alkali metals (lithium, sodium, potassium, rubidium, and cesium) are highly reactive and are pyrophoric. They must be handled with extreme care under an inert atmosphere and protected from contact with air, moisture, and other reactive substances.

^1^H and ^13^C­{^1^H} NMR spectra were recorded at room temperature using JEOL JNM-ECZ 400 MHz and Bruker Avance 600 MHz spectrometers. Chemical shifts are reported in parts per million (ppm) and referenced to residual solvent signals. FTIR-ATR spectra were collected using a Bruker Vertex 70 vacuum spectrometer. ESI-MS data were acquired on a Waters Xevo G3 QTOF mass spectrometer. Measurements were carried out in MeOH/H_2_O mixtures with the addition of a 0.01% CH_3_CN solution of sodium formate (NaOOCH) as an internal calibrant. TGA/DSC measurements were performed using a Mettler-Toledo TGA/DSC 3+ instrument. Analyses were conducted over the temperature range of 30–800 °C at a heating rate of 5 °C min^–1^ under a nitrogen atmosphere (N_2_, 6.0 purity; flow rate 50 mL min^–1^). Powder X-ray diffraction measurements were performed using a Malvern Panalytical Compact X-ray diffractometer (Aeris, 600 W). Phase identification was supported by comparison with reference patterns from the Crystallography Open Database (COD). SEM imaging of pristine PET and PEICT powders, as well as solid residues obtained after alcoholysis, was carried out using a Hitachi S-3400N scanning electron microscope equipped with a Thermo Noran System SIX EDS detector.

Single-crystal X-ray diffraction data were collected at 100 K using an Xcalibur (**8**), Xcalibur Ruby (**14a**), or XtaLAB Synergy R (**9**, **11**, **12**, **21**, **23**, **26**) diffractometer. Experimental details and crystallographic data are summarized in Table S1. Structures were solved by direct methods and refined using the full-matrix least-squares method on *F* ^2^ using the SHELXTL software package.^101^ All nonhydrogen atoms were refined anisotropically. Hydrogen atoms were placed in geometrically calculated positions and included in the structure factor calculations but were not refined. Molecular graphics were generated using Diamond (version 3.1e).^102^ Crystallographic data for the structures reported in this work have been deposited with the Cambridge Crystallographic Data Centre (CCDC) under deposition numbers CCDC 2517543–2517550 and 2521029. These data can be obtained free of charge from the CCDC, 12 Union Road, Cambridge CB2 1EZ, UK (Fax: + 44-1223-336033; E-mail: deposit@ccdc.cam.ac.uk; Web site: https://www.ccdc.cam.ac.uk).

### Synthesis and Spectroscopic Characterization of **1–26**

#### Synthesis of [Rb­(sal-Me)]*_n_* (**4**)

Metallic rubidium (1.00 g, 11.7 mmol) sealed in an ampule, Hsal-Me (1.515 mL, 11.7 mmol), and toluene (10 mL) were introduced into a glass reactor. The mixture was cooled to −50 °C, after which the ampule containing rubidium was mechanically broken. The reaction was allowed to proceed until complete consumption of the metal was observed. The solvent was then removed under reduced pressure to afford the product as a powder. Yield: 2.46 g (89%). ^1^H NMR (400 MHz, THF-d_8_): δ 7.57 (1H, dd, *J* = 8.0. 2.0 Hz, ArH), 6.91 (1H, m, ArH), 6.45 (1H, d, *J* = 8.4 Hz, ArH), 5.95 (1H, m, ArH), 3.61 (3H, s, CH_3_). ^13^C NMR (101 MHz, THF-d_8_): 175.40 (1C, C=O), 169.81 (1C, C–O), 134.08 (1C, ArH), 132.79 (1C, ArH), 124.86 (1C, ArH), 115.25 (1C, Ar), 108.52 (1C, ArH), 50.19 (1C, CH_3_). FTIR-ATR (cm^–1^): 3080 (w), 3050 (m), 3022 (w), 2995 (w), 2954 (m), 2842 (w), 2323 (vw), 2167 (w), 2146 (vw), 2112 (vw), 2033 (w), 1981 (vw), 1927 (vw), 1898 (w), 1821 (vw), 1670 (vs), 1593 (m), 1526 (m), 1464 (s), 1448 (m), 1433 (m), 1391 (m), 1356 (m), 1318 (m), 1258 (m), 1209 (m), 1183 (s), 1153 (m), 1133 (m), 1076 (m), 1036 (m), 977 (w), 968 (w), 951 (m), 848 (m), 793 (m), 764 (m), 708 (s), 648 (m), 567 (m), 535 (m), 422 (m).

#### Synthesis of [Cs­(sal-Me)]_*n*_ (**5**)

Metallic cesium (1.00 g, 7.5 mmol) sealed in an ampule was introduced into a glass reactor. Methyl salicylate (0.975 mL, 7.5 mmol) and toluene (10 mL) were added. The mixture was cooled to −50 °C, after which the ampule containing cesium was mechanically crushed. The reaction was allowed to proceed until complete consumption of the metal was observed. The solvent was removed under reduced pressure to afford the product as a powder. Yield: 1.80 g (84%). ^1^H NMR (400 MHz, THF-d_8_): δ 7.57 (1H, dd, *J* = 8.0, 1.9 Hz, ArH), 6.92 (1H, m, ArH), 6.46 (1H, d, *J* = 8.5 Hz, ArH), 5.95 (1H, m, ArH), 3.61 (3H, s, CH_3_). ^13^C NMR (101 MHz, THF-d_8_): 175.02 (1C, C=O), 169.60 (1C, C–O), 134.11 (1C, ArH), 132.91 (1C, ArH), 124.95 (1C, ArH), 115.44 (1C, Ar), 108.37 (1C, ArH), 50.23 (1C, CH_3_). FTIR-ATR (cm^–1^): 3080 (w), 3049 (m), 3020 (w), 2988 (w), 2949 (m), 2903 (vw), 2838 (w), 2714 (vw), 2315 (vw), 2169 (vw), 2110 (vw), 2034 (w), 1924 (w), 1901 (w), 1820 (vw), 1671 (vs), 1590 (m), 1524 (m), 1461 (m), 1447 (m), 1430 (m), 1388 (m), 1357 (m), 1315 (m), 1315 (m), 1255 (m), 1207 (m), 1175 (m), 1150 (s), 1131 (m), 1067 (m), 1034 (m), 967 (w), 952 (m), 847 (s), 764 (s), 707 (m), 646 (m), 565 (m), 535 (m), 435 (w), 418 (m).

#### Synthesis of [Ca_3_(sal-Me)_6_(EtOH)_2_] (**8**)

Metallic calcium (0.3251 g, 8.11 mmol) and Hsal-Me (2.1 mL, 16.22 mmol) were introduced into a 150 mL Schlenk flask equipped with a magnetic stir bar, along with methanol (20 mL). The mixture was stirred and heated for 12 h, after which ethanol (5 mL) was added. Stirring was continued for an additional 6 h. The solution was then concentrated to approximately half of its original volume and placed in a refrigerator to induce crystallization. The resulting colorless crystals were isolated by filtration, washed with hexane (3 × 10 mL), and dried under vacuum. Yield: 1.74 g (58%). ^1^H NMR (400 MHz, THF-d_8_): δ 7.70 (6H, m, ArH), 7.16 (6H, m, ArH), 6.89 (6H, d, *J* = 8.2 Hz, ArH), 6.47 (6H, m, ArH), 4.24 (4H, m, EtOH), 3.74 (18H, m, CH_3_), 1.30 (6H, t, *J* = 6.7 Hz, EtOH).^13^C NMR (101 MHz, THF-d_8_): 172.08 (6C, C=O), {171.32, 171.08, 170.98 (6C, C–O)}, 135.04 (6C, ArH), {132.19, 131.53 (6C, ArH)}, {124.58, 124.28 (6C, ArH)}, {116.55, 115.02, 114.07 (6C, ArH)}, {112.85, 111.91 (6C, Ar)}, {51.71, 51.46 (6C, CH_3_)}. FTIR-ATR (cm^–1^): 3323 (vw), 3064 (w), 3022 (w), 2989 (w), 2944 (m), 2847 (w), 2678 (vw), 2323 (vw), 2050 (vw), 1909 (vw), 1800 (vw), 1654 (vs), 1600 (m), 1543 (m), 1469 (m), 1437 (m), 1391 (w), 1371 (w), 1326 (m), 1219 (s), 1158 (m), 1085 (m), 1039 (m), 964 (m), 949 (m), 888 (w), 865 (m), 853 (m), 819 (m), 797 (m), 749 (s), 706 (s), 658 (m), 579 (m), 555 (m), 532 (m), 444 (m), 424 (m).

#### Synthesis of [MgRb­(μ-H_2_O)­(sal-Me)_3_(H_2_O)_0.5_]_*n*_ (**12**)

A rubidium methyl salicylate compound [Rb­(sal-Me)]_*n*_ (**4**) (0.3465 g, 1.46 mmol) and [Mg_2_Li_2_(sal-Me)_6_] (**9**) (0.7090 g, 0.73 mmol) were dissolved in THF (40 mL) in a 150 mL Schlenk flask equipped with a stir bar. The mixture was stirred and heated for 12 h, then concentrated to half its volume and left to crystallize in a refrigerator. Colorless crystals were collected by filtration, washed with hexane (3 × 10 mL), and dried under vacuum. Yield: 0.52 g (59%). ^1^H NMR (400 MHz, THF-d_8_): δ 7.63 (3H, d, *J* = 6.9 Hz, ArH), 7.08 (3H, m, ArH), 6.65 (3H, m, ArH), 6.27 (3H, m, ArH), 3.68 (9H, s, CH_3_). ^13^C NMR (101 MHz, THF-d_8_): 172.93 (3C, C=O), 171.91 (3C, C–O), 135.12 (3C, ArH), 131.51 (3C, ArH), 124.38 (3C, ArH), 114.24 (3C, Ar), 112.31 (3C, ArH), 51.43 (3C, CH_3_). FTIR-ATR (cm^–1^): 3060 (vw), 3018 (vw), 2950 (w), 2845 (vw), 2790 (vw), 2576 (vw), 1654 (s), 1601 (m), 1544 (m), 1501 (w), 1472 (m), 1448 (m), 1437 (m), 1337 (m), 1325 (m), 1261 (m), 1225 (vs), 1195 (m), 1155 (m), 1087 (m), 1046 (m), 964 (m), 861 (m), 829 (w), 818 (m), 795 (m), 753 (s), 706 (m), 661 (m), 589 (m), 569 (m), 532 (m), 453 (m), 438 (m), 409 (m).

#### Synthesis of [Zn_2_Na_2_(sal-R)_6_]*_**n**_* **(14a**, for R = Me (0.86), Et(0.14))

Metallic sodium (0.180 g, 7.83 mmol) and Hsal-Me (3.00 mL, 23.4 mmol) were suspended in toluene (25 mL) in a 150 mL Schlenk flask and stirred at ambient temperature for 6 h. ZnEt_2_ (7.8 mL, 7.83 mmol) was then added dropwise, and the reaction mixture was stirred for an additional 12 h. Ethanol (5 mL) was added, and the mixture was stirred for 3 h. The solution was concentrated to half of its volume and placed in a refrigerator to crystallize. The resulting colorless crystals were filtered off, washed with hexane (3 × 10 mL), and dried under vacuum. Yield: 2.7 g (64%). ^1^H NMR (400 MHz, THF-d_8_): δ 7.66 (6H, m, ArH), 6.90 (6H, m, ArH), 6.84 (6H, m, ArH), 6.25 (6H, m, ArH), 3.79 (18H, s, CH_3_). ^13^C NMR (101 MHz, THF-d_8_): δ 170.62 (6C, C=O), 169.58 (6C, C–O), 134.61 (6C, ArH), 132.03 (6C, ArH), 123.47 (6C, ArH), 116.50 (6C, Ar), 113.75 (6C, ArH), 51.06 (6C, CH_3_). FTIR-ATR (cm^–1^): 3046 (vw), 3023 (w), 2950 (w), 2907 (vw), 2841 (w), 2656 (vw), 1677 (s) 1642 (m), 1597 (m), 1550 (m), 1470 (m), 1439 (s), 1318 (m), 1260 (m), 1218 (vs), 1193 (m), 1156 (m), 1085 (m), 1041 (m), 964 (w), 858 (m), 814 (m), 796 (w), 753 (s), 707 (m), 661 (m), 584 (m), 534 (m), 475 (m), 456 (m), 434 (m).

**Synthesis of [M′_6_(sal-Me)_6_] (for M′^+^ = Li^+^ (1), Na^+^ (2), K^+^ (3)), [Mg_2_(sal-Et)_4_(EtOH)_2_] (6), [Zn_4_(sal-Me)_8_] (7), [M_2_M′_2_(sal-Me)_6_(THF)_*x*_] (for M^2+^ = Mg^2+^ and M′^+^ = Li^+^ (9), Na^+^ (10), K^+^ (11); M^2+^ = Zn^2+^ and M′^+^ = Li^+^ (13), Na^+^ (14), K^+^ (15); and *x* = 0, 2, 4), [Ca_2_Li_2_(sal-Me)_6_(THF)_2_] (16), [Ca_2_M′(sal-Me)_6_(MeOH)_4_] (for M′^+^ = Na^+^ (17), K^+^ (18)), [Mg_4_Na_2_(sal-Me)_6_(sal)_2_(THF)_4_] (19), [CaLi_6_(sal-Me)_8_] (20), [Ca_4_Na­(μ_5_-OH)­(sal-Et)_8_(EtOH)] (21), and [Ca_4_K­(μ_5_-OH)­(sal-Et)_8_(EtOH)] (22)**

Compounds **1**–**3**, **6**, **7**, **9**–**11**, and **13**–**22** for catalytic applications were synthesized using previously published procedures.^80–84^

#### Synthesis of Heterometallic Magnesium–Alkali Metal Compounds with Mg:M′ Stoichiometry of **1**:**2**

##### Synthesis of [Li_6_(sal-Me)_6_] (**1**) and [Mg_2_Li_2_(sal-Me)_6_] (**9**)

To a 150 mL Schlenk flask equipped with a stir bar, metallic lithium (0.0416 g, 5.99 mmol), Hsal-Me (1.605 mL, 12.0 mmol), THF (25 mL), and toluene (10 mL) were introduced and stirred at ambient temperature for 4 h. MgBu_2_ (3.0 mL, 3.0 mmol) was then added dropwise, and stirring was continued for 12 h. The solution was concentrated to half of its volume and left to crystallize in a refrigerator. Colorless crystals were isolated by filtration, washed with hexane (3 × 10 mL), and dried under vacuum. Spectroscopic and PXRD analysis indicated the formation of a mixture of compounds **1** and **9**. A new polymorphic form of **9** was obtained in crystalline form.

##### Synthesis of [Na_6_(sal-Me)_6_] (**2**) and [Mg_2_Na_2_(sal-Me)_6_(THF)_4_] (**10**)

To a 150 mL Schlenk flask equipped with a stir bar, metallic sodium (0.1552 g, 6.75 mmol), Hsal-Me (1.750 mL, 13.5 mmol), THF (25 mL), and toluene (10 mL) were introduced and stirred at ambient temperature for 4 h. MgBu_2_ (3.40 mL, 3.40 mmol) was added dropwise, and the reaction was stirred for an additional 12 h. The solution was concentrated to half of its volume and left to crystallize in a refrigerator. The colorless crystals were filtered, washed with hexane (3 × 10 mL), and dried under vacuum. Spectroscopic and PXRD analysis revealed the formation of a mixture of compounds **2** and **10**.

##### [K_6_(sal-Me)_6_] (**3**) and [Mg_2_K_2_(sal-Me)_6_(THF)_2_] (**11**)

To a 150 mL Schlenk flask equipped with a stir bar, metallic potassium (0.2200 g, 5.63 mmol), Hsal-Me (1.450 mL, 11.3 mmol), THF (25 mL), and toluene (10 mL) were introduced and stirred at ambient temperature for 4 h. MgBu_2_ (2.80 mL, 2.80 mmol) was added dropwise, followed by stirring for 12 h. The solution was concentrated to half of its volume and placed in a refrigerator. The resulting crystals were filtered, washed with hexane (3 × 10 mL), and dried under vacuum. Spectroscopic and PXRD analysis showed the formation of a mixture of compounds **3** and **11**. A new polymorphic form of **11** was obtained.

#### Synthesis of [ZnLi_2_(sal-Me)_4_(THF)_2_] (**23**)

To a 150 mL Schlenk flask equipped with a stir bar, metallic lithium (0.041 g, 6 mmol), Hsal-Me (1.6 mL, 12 mmol), toluene (20 mL), and THF (10 mL) were introduced, and the reaction was stirred at ambient temperature for 4 h. ZnEt_2_ (3 mL, 3 mmol) was added dropwise, and the reaction was continued for 10 h. The solution was concentrated to half of its volume and left to crystallize in a refrigerator. Colorless crystals were isolated by filtration, washed with hexane (3 × 10 mL), and dried under vacuum. Yield: 0.73 g (30%). ^1^H NMR (400 MHz, THF-d_8_): δ 7.72 (4H, dd, *J* = 8.0, 1.8 Hz, ArH), 7.00 (4H, t, *J* = 7.6 Hz, ArH), 6.70 (4H, d, *J* = 8.4 Hz, ArH), 6.35 (4H, t, *J* = 7.4 Hz), 3.89 (12H, s, CH_3_), 3.62 (8H, m, THF), 1.77 (8H, m, THF). ^13^C NMR (101 MHz, THF-d_8_): δ 169.90 (4C, C=O; 4C, C–O), 135.11 (4C, ArH), 131.80 (4C, ArH), 122.81 (4C, ArH), 115.79 (4C, Ar), 114.48 (4C, ArH), 51.31 (4C, CH_3_). FTIR-ATR (cm^–1^): 3021 (w), 2978 (w), 2948 (m), 2874 (w), 2844 (w), 2661 (vw), 2323 (vw), 1680 (vs), 1596 (m), 1552 (m), 1505 (w), 1470 (m), 1446 (m), 1394 (w), 1329 (m), 1318 (m), 1263 (m), 1225 (s), 1191 (m), 1157 (m), 1142 (m), 1084 (m), 1051 (m), 1039 (m), 965 (w), 950 (w), 914 (m), 901 (m), 860 (m), 817 (m), 797 (m), 757 (m), 706 (m), 664 (m), 600 (m), 590 (m), 571 (m), 534 (m), 485 (m), 473 (m).

#### Synthesis of [ZnNa_2_(sal-Et)_4_]_*n*_ (**24**)

Metallic sodium (0.1773 g, 7.70 mmol), Hsal-Me (2.00 mL, 15.4 mmol), toluene (10 mL), and ethanol (20 mL) were introduced into a 150 mL Schlenk flask and stirred at ambient temperature for 3 h. ZnEt_2_ (3.85 mL, 3.85 mmol) was added dropwise, and the reaction mixture was stirred for an additional 12 h. The solution was concentrated to half of its volume and left to crystallize in a refrigerator. The colorless crystals were filtered, washed with hexane (3 × 10 mL), and dried under vacuum. Yield: 1.08 g (36%). ^1^H NMR (400 MHz, THF-d_8_): δ 7.79 (4H, dd, *J* = 24.5, 6.4 Hz, ArH), 7.38 (4H, m, ArH), 6.88 (4H, d, *J* = 8.1 Hz, ArH), 6.75 (4H, m, ArH), 4.37 (8H, d, *J* = 5.8 Hz, CH_2_), 1.37 (12H, t, *J* = 7.1 Hz, CH_3_). ^13^C NMR (101 MHz, THF-d_8_): 170.73 (4C, C=O), 169.32 (4C, C–O), 135.88 (4C, ArH), {132.16, 131.24, 130.64} (4C, ArH), {129.48, 128.72} (4C, ArH), 118.85 (4C, ArH), {116.84, 116.59} (4C, Ar), 61.62 (4C, CH_2_), 14.31 (4C, CH_3_). FTIR-ATR (cm^–1^): 3347 (vw), 2986 (w), 2934 (vw), 2907 (vw), 1708 (m), 1677 (m), 1547 (m), 1486 (w), 1437 (s), 1391 (m), 1367 (m), 1317 (m), 1301 (m), 1254 (m), 1233 (m), 1213 (m), 1161 (m), 1146 (m), 1111 (vw), 1085 (m), 1040 (w), 1021 (w), 959 (vw), 947 (vw), 888 (m), 860 (m), 840 (m), 816 (m), 801 (m), 755 (vw), 714 (m), 702 (m), 663 (m), 572 (m), 531 (m), 472 (m), 454 (m), 430 (w).

#### Synthesis of [ZnK_2_(sal-Me)_4_]_*n*_ (**25**)

Metallic potassium (0.1047 g, 2.68 mmol), Hsal-Me (0.695 mL, 5.36 mmol), and THF (50 mL) were introduced into a 150 mL Schlenk flask and stirred at ambient temperature for 2 h. ZnEt_2_ (1.35 mL, 1.34 mmol) was added dropwise, and the reaction was stirred for an additional 12 h. The solution was concentrated to half of its volume, and the resulting white precipitate was isolated by filtration, washed with hexane (3 × 10 mL), and dried under vacuum. Yield: 0.92 g (91%). ^1^H NMR (400 MHz, THF-d_8_): δ 7.58 (4H, d, *J* = 5.4 Hz, ArH), 6.98 (4H, m, ArH), 6.89 (4H, m, ArH), 6.30 (4H, m, ArH), 3.72 (12H, s, CH_3_). ^13^C NMR (101 MHz, THF-d_8_): 171.42 (4C, C=O), 169.70 (4C, C–O), 134.49 (4C, ArH), 131.50 (4C, ArH), 123.18 (4C, ArH), 116.39 (4C, Ar), 113.80 (4C, ArH), 51.06 (4C, CH_3_). FTIR-ATR (cm^–1^): 3285 (vw), 3063 (w), 3020 (w), 2953 (w), 1847 (w), 2667 (vw), 1701 (m), 1675 (m), 1642 (m), 1596 (m), 1541 (m), 1468 (m), 1441 (m), 1322 (m), 1258 (m), 1221 (s), 1192 (m), 1155 (m), 1140 (m), 1081 (m), 1032 (m), 965 (w), 948 (m), 860 (m), 811 (m), 801 (m), 755 (vs), 709 (m), 659 (m), 584 (m), 564 (m), 534 (m), 482 (m), 448 (m), 428 (m).

### Alcoholysis of PET

Alcoholysis of PET (0.4 g, 2.08 mmol) using *n*-BuOH (4.75 mL, 51.89 mmol) as the reagent and homo- and heterometallic aryloxides as catalysts was investigated as a chemical recycling route for postconsumer PET. Reactions were carried out in *n*-BuOH at temperatures ranging from 120 to 220 °C for 2 h in 25 mL PTFE-lined hydrothermal reactors. Catalysts **1–25** were employed using a stoichiometry of [TA_(PET)_/*n*-BuOH/M] = 1/25/0.05. The progress of the alcoholysis reactions and the catalytic activities of compounds **1–25** were monitored over time by ^1^H NMR spectroscopy and quantified based on the amount of isolated, unreacted polymer residue.

### DBT

^1^H NMR (400 MHz, DMSO-d_6_): δ 8.06 (4H, s, ArH), 4.29 (4H, t, *J* = 6.5 Hz, OCH_2_), 1.69 (4H, m, CH_2_), 1.42 (4H, m, CH_2_), 0.92 (6H, t, *J* = 7.4 Hz, CH_3_). ^13^C NMR (101 MHz, DMSO-d_6_): 165.03 (2C, C=O), 133.72 (2C, Ar), 129.44 (4C, ArH), 64.86 (2C, OCH_2_), 30.17 (2C, CH_2_), 18.72 (2C, CH_2_), 13.58 (2C, CH_3_). FTIR-ATR (cm^–1^): 3539 (vw), 3427 (vw), 2960 (m), 2935 (m), 2874 (m), 1984 (vw), 1717 (vs), 1681 (m), 1614 (w), 1578 (w), 1505 (w), 1465 (m), 1408 (m), 1384 (m), 1356 (w), 1256 (s), 1248 (s), 1172 (w), 1155 (m), 1113 (m), 1100 (m), 1019 (m), 993 (w), 963 (m), 943 (m), 902 (w), 875 (m), 842 (m), 796 (m), 728 (s), 632 (w), 497 (m), 435 (m).

### Alcoholysis of PEICT

Alcoholysis of PEICT (0.3 g, 0.404 mmol) using *n*-BuOH (2.84 mL, 31.02 mmol) as the reagent and homo- and heterometallic aryloxides as catalysts was studied as a chemical recycling method for postconsumer PEICT. The reactions were conducted in *n*-BuOH at 140–240 °C for 2 h using 25 mL PTFE-lined hydrothermal reactors. Catalysts **1–25** were applied with a stoichiometry of [TA_(PEICT)_/*n*-BuOH/M] = 1/25/0.025. Reaction progress and catalytic activity were evaluated over time by ^1^H NMR spectroscopy and quantified based on the amount of isolated, unreacted polymer residue.

### Synthesis of [Li­(BTA)]*_n_* (**26**)

The reaction was carried out in four PTFE-lined hydrothermal reactors. In each reactor, PET (0.80 g, 4.16 mmol), [Li_6_(sal-Me)_6_] (**1**) (0.04 g, 0.042 mmol), and *n*-BuOH (9.5 mL) were introduced. The reactions were conducted at 180 °C for 14 h. The contents of all reactors were combined, and excess alcohol was removed by distillation. The resulting mixture was exposed to atmospheric moisture for 48 h. Yield: 0.07 g (12%). ^1^H NMR (400 MHz, THF-d_8_): δ 8.10 (2H, d, *J* = 8.1 Hz, ArH), 7.89 (2H, d, *J* = 8.1 Hz, ArH), 4.23 (2H, t, *J* = 6.5 Hz, OCH_2_), 1.65 (2H, m, CH_2_), 1.42 (2H, m, CH_2_), 0.93 (3H, t, *J* = 7.4 Hz, CH_3_). ^13^C NMR (101 MHz, THF-d_8_): 172.13 (1C, C=O), 166.32 (1C, C=O), 143.14 (1C, Ar), 132.33 (1C, Ar), 130.25 (2C, ArH), 129.10 (2C, ArH), 64.91 (1C, OCH_2_), 31.61 (1C, CH_2_), 19.98 (1C, CH_2_), 13.94 (1C, CH_3_). FTIR-ATR (cm^–1^): 3367 (vw), 3061 (vw), 2960 (m), 2935 (m), 2874 (m), 1948 (w), 1717 (m), 1680 (m), 1617 (m), 1594 (m), 1557 (m), 1506 (m), 1462 (m), 1418 (m), 1405 (m), 1390 (m), 1341 (w), 1271 (s), 1177 (m), 1154 (m), 1137 (m), 1111 (m), 1059 (m), 1018 (m), 989 (w), 967 (w), 936 (m), 879 (m), 856 (w), 828 (w), 810 (m), 735 (vs), 705 (m), 668 (w), 505 (m), 409 (m). The isolated compound contained approximately 1% Na^+^ impurity.

## Supplementary Material



## Notes

Additional crystallographic data for **8, 9**, **11, 12**, **14a**, **21**, **23, 24**, **26**; FTIR-ATR, ^1^H, and ^13^C NMR spectra for **4**, **5**, **8**, **12–15**, **23–26**; FTIR-ATR, NMR, and ESI-MS data of organic products obtained from PET/PEICT alcoholysis; and summary of PET/PEICT alcoholysis (PDF)
