# Supplementary material for: Sustainable Recovery of Dibutyl Terephthalate from Aromatic Polyester Waste via Solvothermal Alcoholysis
Source: Inorg Chem. 2026 Aug 21;65(35):20201–21. doi: 10.1021/acs.inorgchem.6c01014 (PMC13556131; doi:10.1021/acs.inorgchem.6c01014)
Supplement: Supplementary file 1 [file ic6c01014_si_001.pdf]

# Supporting information for

## Sustainable Recovery of Dibutyl Terephthalate from Aromatic Polyester Waste via Solvothermal Alcoholysis

Karolina Matuszak,[1] Rafał Petrus,[1,*] and Tadeusz Lis[2]

[1] *Faculty of Chemistry, Wrocław University of Science and Technology,*

*23 Smoluchowskiego, 50-370 Wrocław, Poland*

[2] *Faculty of Chemistry, University of Wrocław, 14 F. Joliot-Curie, 50-383 Wrocław, Poland.*

[*]*Corresponding author email:* rafal.petrus@pwr.edu.pl

**Contents**

X-Ray Crystallography of **8-9**, **11-12**, **14a**, **21**, **23-24**, **26**……………..……………………S2

FTIR, $^1$H, $^1$H DOSY, and $^{13}$C NMR spectra of **4** and **5**……………………………….…….S4

Crystal structure, FTIR, and NMR data of **8**…………………………...…………………...S8

Formula weights (FWs) of **4**, **5**, **12-15**, **23-26** estimated from $^1$H DOSY NMR spectra.......S8

Crystal structure of **21**…………………….…………………………………………………...S10

Continuous-shape measurements of the coordination environment around metal ions in **8**, **12**, **14a**, **23**, **24**, and **26**……………………………………………………………………….S11

FTIR, $^1$H, $^1$H DOSY, and $^{13}$C NMR spectra of **12** and **14a**………………………….…......S11

$^1$H DOSY NMR spectra of **13-15**…………………………….…………………………..S14

FTIR spectra and PXRD patterns of **1-3** and **9-11**……………………………………......S17

Crystal structures of **9** and **11**……………………………………………………………….S19

FTIR, $^1$H, $^1$H-DOSY, and $^{13}$C-NMR spectra of **23-25**………………………………………S23

FTIR-ATR, NMR, PXRD, and ESI-MS data of organic products and catalyst residues obtained from PET/PEICT alcoholysis…………………………………………………..S26

Summary of PET alcoholysis in the presence of **1-25** at 120 -240°C…………………….…..S39

Summary of PEICT alcoholysis in the presence of **1-25** at 140 - 240°C…………………...S41

## Crystallographic Data for Compounds 8-9, 11-12, 14a, 21, 23-24, 26.

**Table S1A.** Crystal and data collection parameters for **8-9**, **11-12**, and **14a**.

| Crystal | **8**·4($C_2H_6O$) | **9** | **11** | **12**·2($H_2O$) | **14a** |
|---|---|---|---|---|---|
| Chemical formula | $C_{52}H_{54}Ca_3O_{20}$ | $C_{48}H_{42}Li_2Mg_2O_{18}$ | $C_{64}H_{74}K_2Mg_2O_{22}$ | $C_{24}H_{24}MgO_{10.50}Rb$ | $C_{48.81}H_{43.62}Na_2O_{18}Zn_2$ |
| Formula Mass | 1303.46 | 969.31 | 1322.05 | 626.24 | 1094.97 |
| Crystal system | Triclinic | Monoclinic | Triclinic | Monoclinic | Triclinic |
| Space group | $P\bar{1}$ | $P2_1/n$ | $P\bar{1}$ | $P2_1$ | $P\bar{1}$ |
| $a$/Å | 11.477(3) | 11.413(2) | 11.439(3) | 12.775(3) | 9.809(2) |
| $b$/Å | 12.468(4) | 12.120(2) | 12.822(4) | 8.9600(17) | 10.387(3) |
| $c$/Å | 12.561(3) | 17.484(3) | 12.839(3) | 12.880(3) | 13.261(4) |
| $\alpha$/° | 104.98(3) | | 62.61(3) | | 104.13(3) |
| $\beta$/° | 102.93(3) | 104.48(3) | 76.72(2) | 98.24(2) | 110.22(3) |
| $\gamma$/° | 103.46(3) | | 73.56(2) | | 95.89(2) |
| Unit cell volume/Å$^3$ | 1609.7(8) | 2341.7(8) | 1592.7(9) | 1459.1(6) | 1203.2(6) |
| Temperature/K | 100(2) | 100(2) | 100(2) | 100(2) | 100(2) |
| $Z$ | 1 | 2 | 1 | 2 | 1 |
| Radiation type | MoKα | CuKα | MoKα | MoKα | MoKα |
| Absorption coefficient, $\mu$/mm$^{-1}$ | 0.335 | 1.114 | 0.247 | 1.777 | 1.091 |
| No. of reflections measured | 14164 | 35309 | 37221 | 30476 | 12819 |
| No. of independent reflections | 7465 | 4309 | 6938 | 6365 | 5237 |
| No. of observed reflections ($I > 2\sigma(I)$) | 5175 | 3963 | 6341 | 5876 | 4546 |
| $R_{int}$ | 0.0494 | 0.0225 | 0.0382 | 0.0303 | 0.0234 |
| Final $R_1$ values ($I > 2\sigma(I)$) | 0.0802 | 0.0437 | 0.0416 | 0.0312 | 0.0313 |
| Final $wR(F^2)$ values ($I > 2\sigma(I)$) | 0.2136 | 0.1202 | 0.1184 | 0.0753 | 0.0712 |
| Final $R_1$ values (all data) | 0.1112 | 0.0469 | 0.0449 | 0.0363 | 0.0402 |
| Final $wR(F^2)$ values (all data) | 0.2530 | 0.1226 | 0.1212 | 0.0778 | 0.0756 |
| Goodness of fit on $F^2$ | 1.035 | 1.073 | 1.030 | 1.052 | 1.046 |
| Δρmax/eÅ$^{-3}$ | 0.902 | 1.013 | 0.547 | 0.416 | 0.552 |
| Δρmin/eÅ$^{-3}$ | -0.774 | -0.270 | -0.528 | -0.297 | -0.325 |

**Table S1B.** Crystal and data collection parameters for **21**, **23-24**, and **26**.

| Crystal | **21** | **23** | **24**·($C_7H_8$) | **26** |
|---|---|---|---|---|
| Chemical formula | $C_{74}H_{79}Ca_4NaO_{26}$ | $C_{40}H_{44}Li_2O_{14}Zn$ | $C_{36}H_{36}Na_2O_{12}Zn$ | $C_{12}H_{13}Li_{0.98}Na_{0.02}O_4$ |
| Formula Mass | 1567.68 | 828.00 | 864.13 | 228.40 |
| Crystal system | Monoclinic | Monoclinic | Monoclinic | Monoclinic |
| Space group | *P*2/n | $P2_1$/n | *C*2/c | *I2/a* |
| *a*/Å | 22.635(5) | 10.0401(10) | 21.919(4) | 14.485(2) |
| *b*/Å | 13.039(3) | 21.232(2) | 12.087(2) | 12.2514(16) |
| *c*/Å | 26.972(8) | 18.6916(16) | 15.721(3) | 26.667(7) |
| *α*/° | | | | |
| *β*/° | 110.98(3) | 98.26(2) | 91.51(2) | 102.22(2) |
| *γ*/° | | | | |
| Unit cell volume/Å$^3$ | 7433(4) | 3943.2(7) | 4163.6(13) | 4625.1(15) |
| Temperature/K | 100(2) | 100(2) | 100(2) | 100(2) |
| *Z* | 4 | 4 | 4 | 16 |
| Radiation type | CuKα | MoKα | MoKα | MoKα |
| Absorption coefficient, $\mu$/mm$^{-1}$ | 3.280 | 0.691 | 0.673 | 0.097 |
| No. of reflections measured | 68658 | 109887 | 24618 | 123300 |
| No. of independent reflections | 13163 | 10486 | 4527 | 6143 |
| No. of observed reflections ($I > 2\sigma(I)$) | 8129 | 9573 | 4221 | 4907 |
| $R_{int}$ | 0.0938 | 0.0271 | 0.0645 | 0.0627 |
| Final $R_1$ values ($I > 2\sigma(I)$) | 0.0994 | 0.0267 | 0.0375 | 0.0593 |
| Final $wR(F^2)$ values ($I > 2\sigma(I)$) | 0.2697 | 0.0677 | 0.0994 | 0.1442 |
| Final $R_1$ values (all data) | 0.1423 | 0.0307 | 0.0399 | 0.0748 |
| Final $wR(F^2)$ values (all data) | 0.3011 | 0.0694 | 0.1028 | 0.1539 |
| Goodness of fit on $F^2$ | 1.034 | 1.027 | 1.057 | 1.027 |
| Δρmax/eÅ$^{-3}$ | 1.473 | 0.433 | 0.404 | 0.640 |
| Δρmin/eÅ$^{-3}$ | -0.866 | -0.319 | -0.385 | -0.602 |

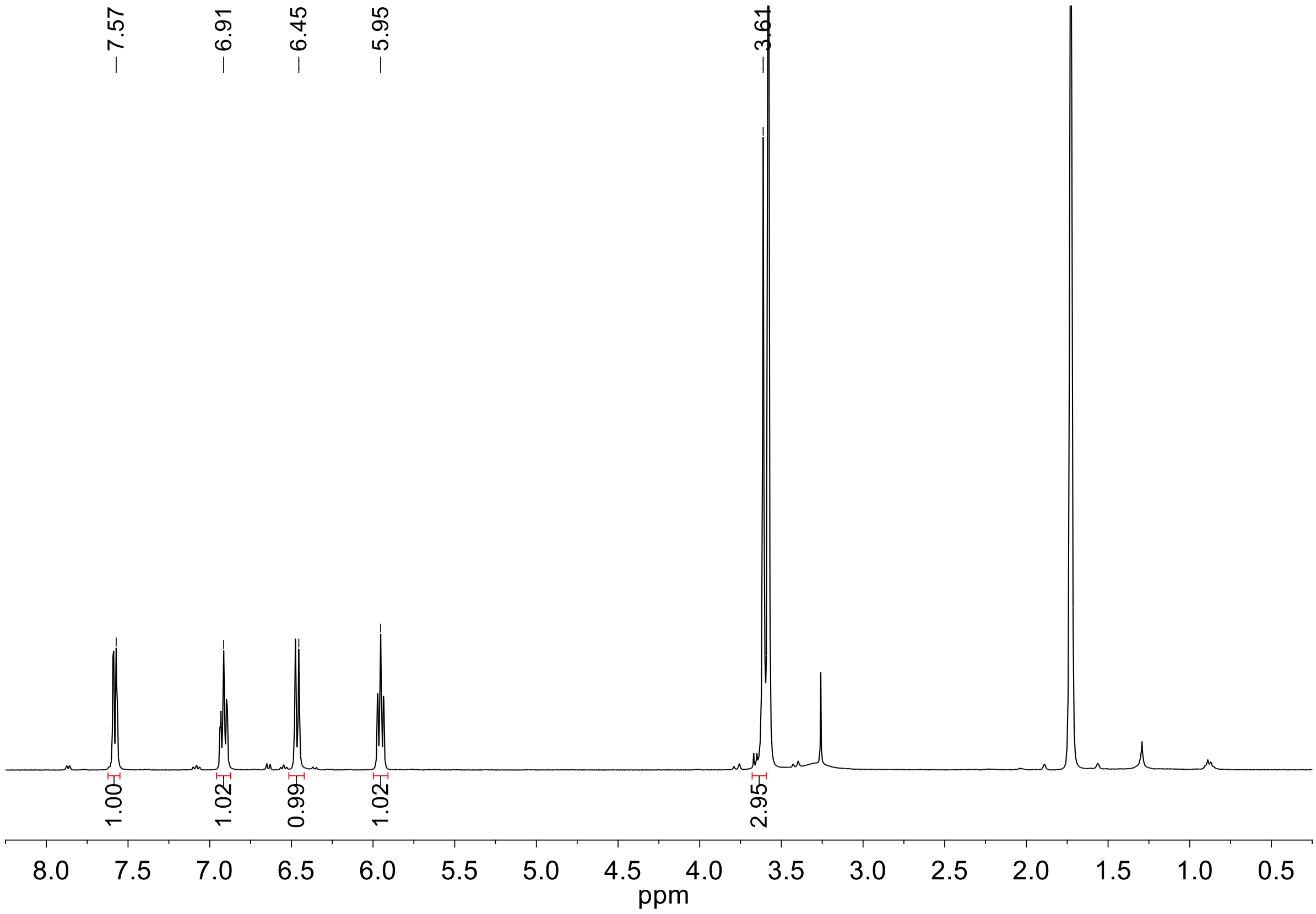


**Figure S1.** $^{1}H$ NMR spectrum of **4** in THF-$d_8$.

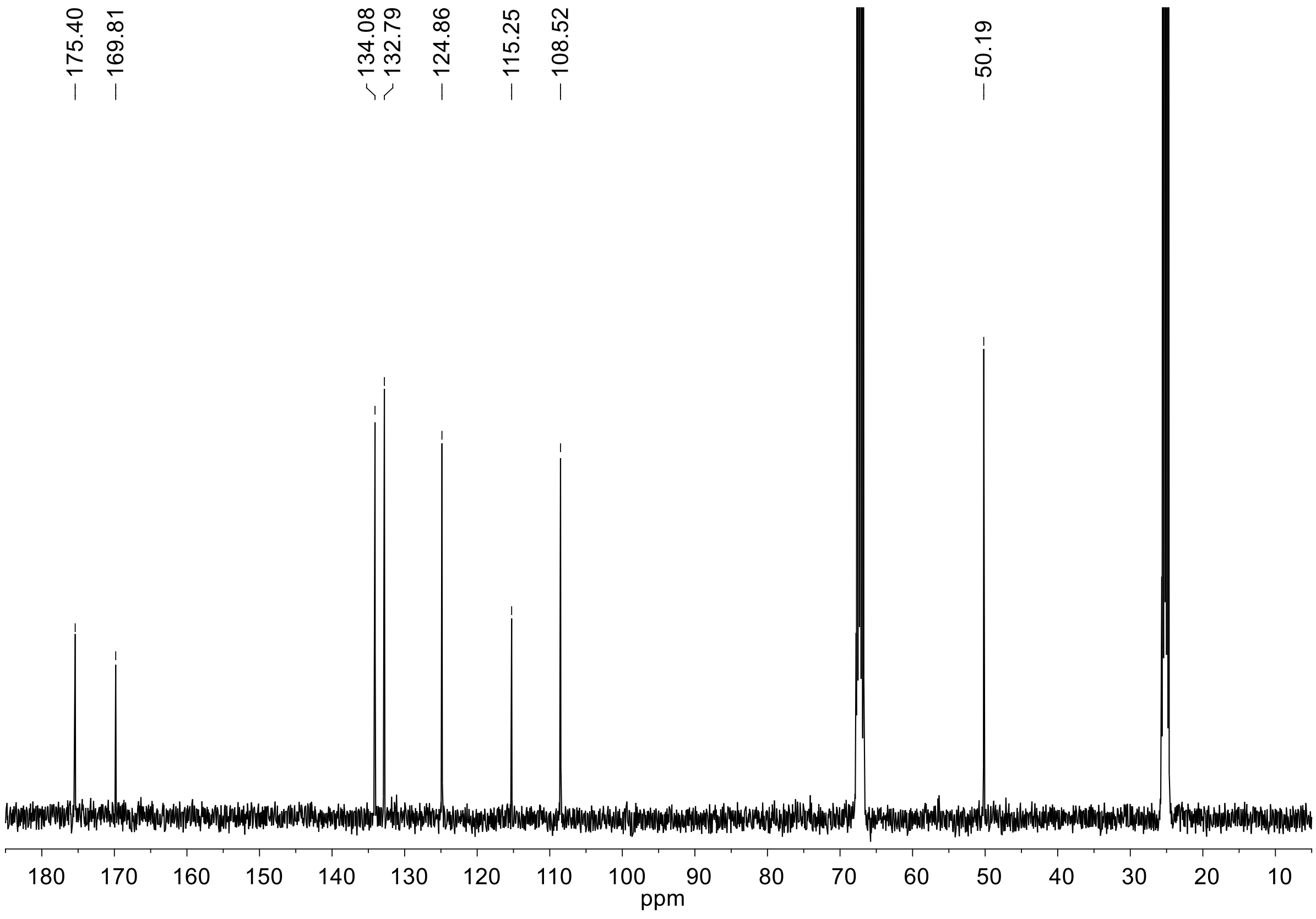


**Figure S2.** $^{13}C$ NMR spectrum of **4** in THF-$d_8$.

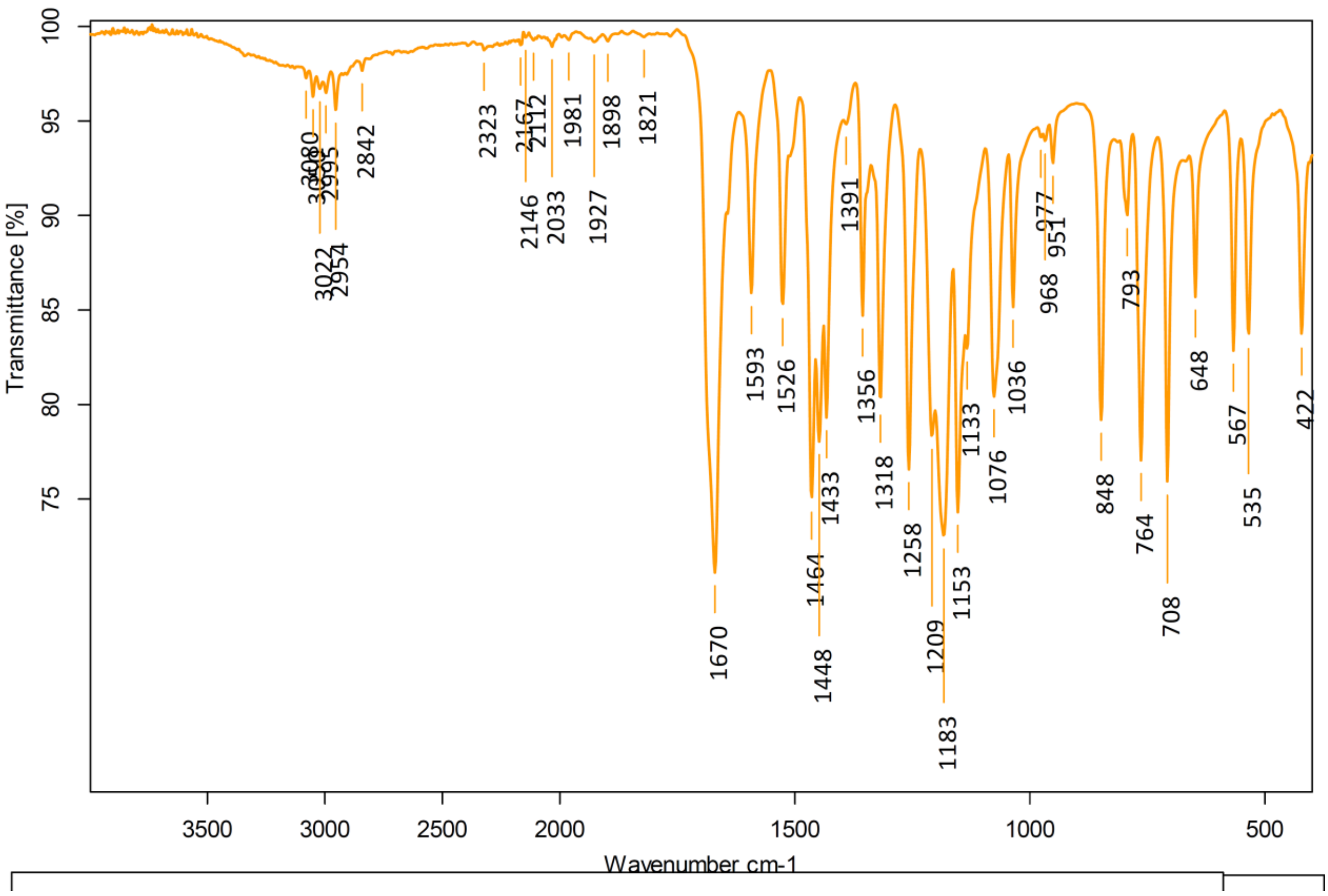


**Figure S3**. FTIR-ATR spectrum of **4**.

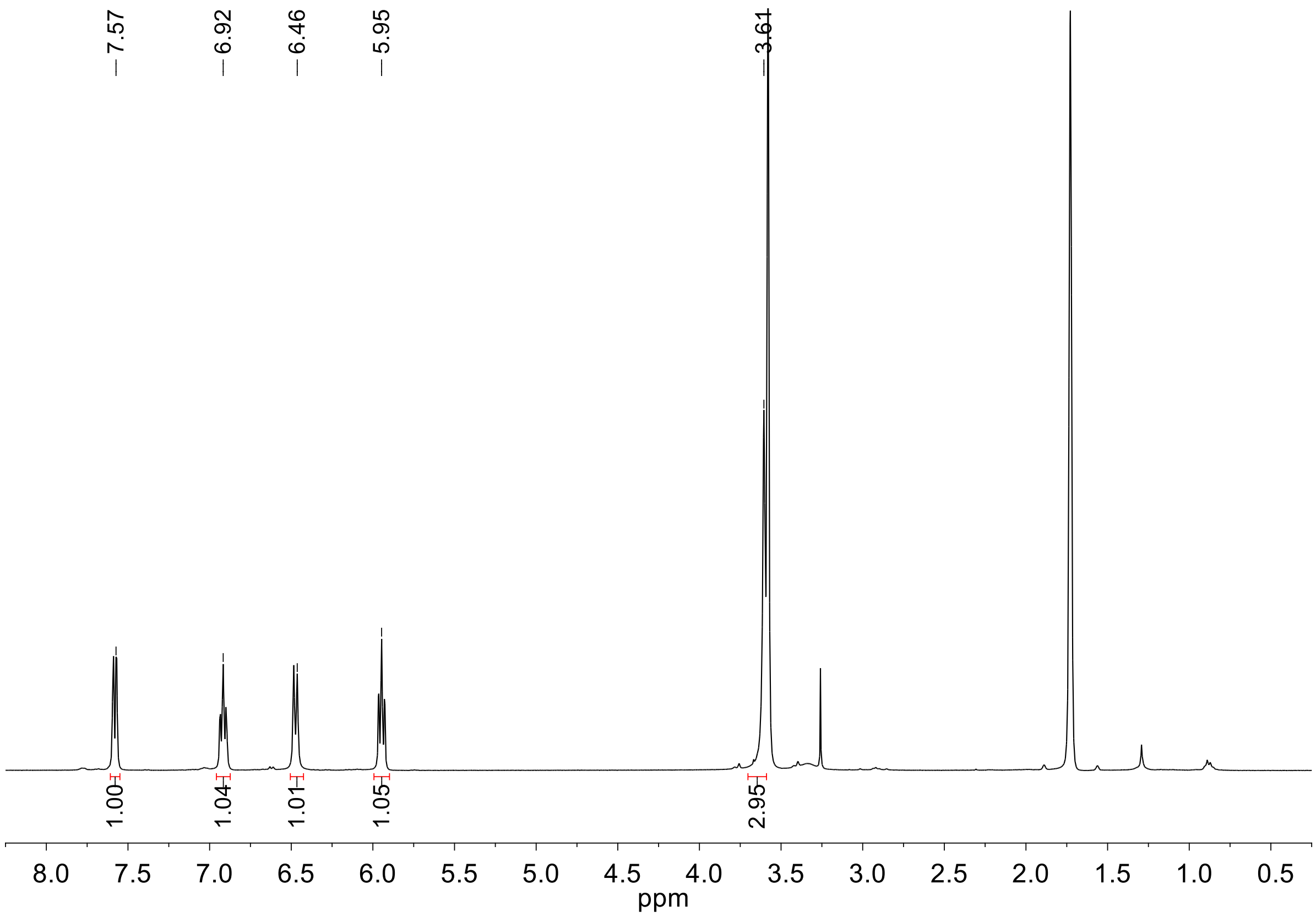


**Figure S4.** $^1$H NMR spectrum of **5** in THF-$d_8$.

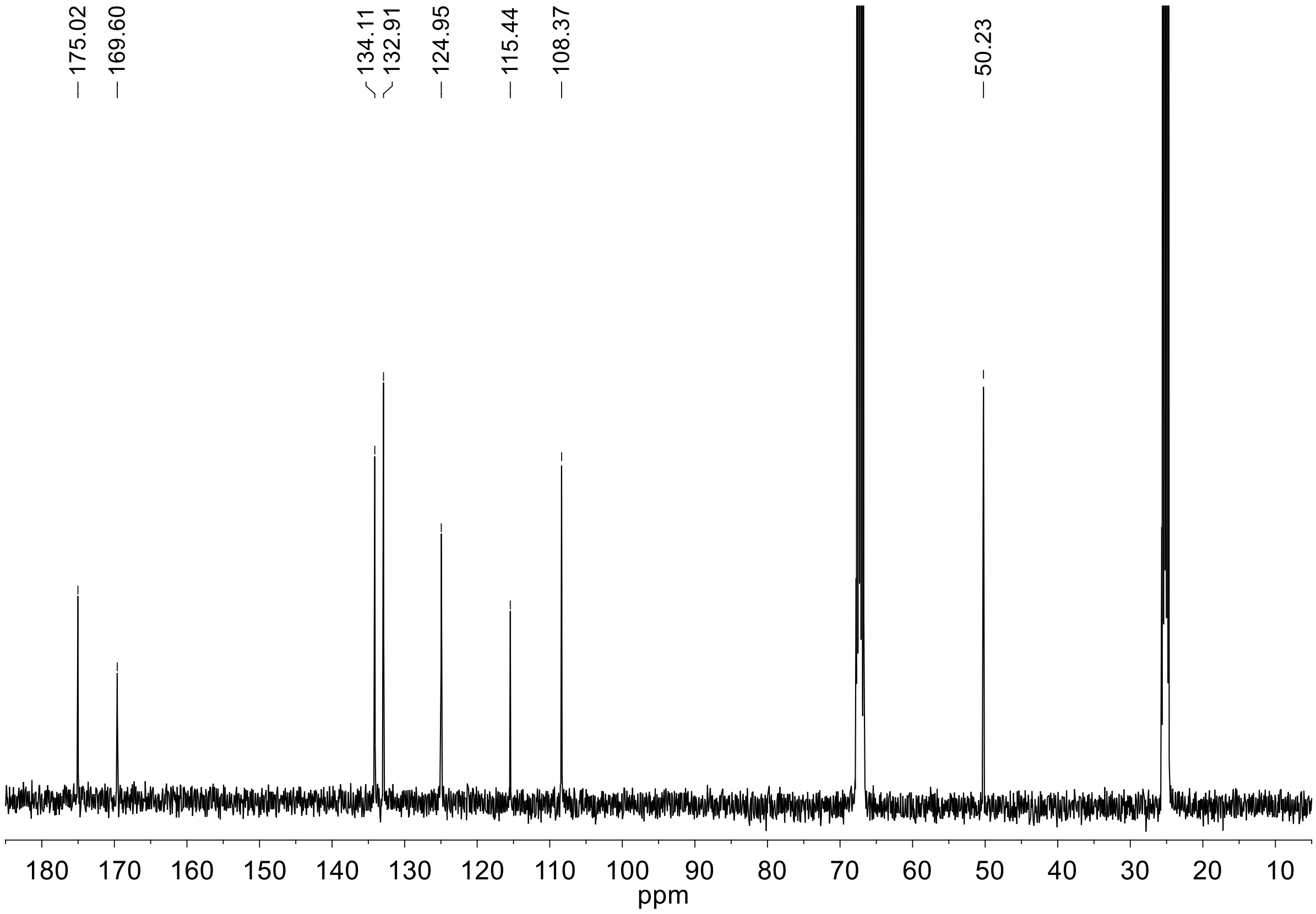


**Figure S5.** $^{13}$C NMR spectrum of **5** in THF-d$_8$.

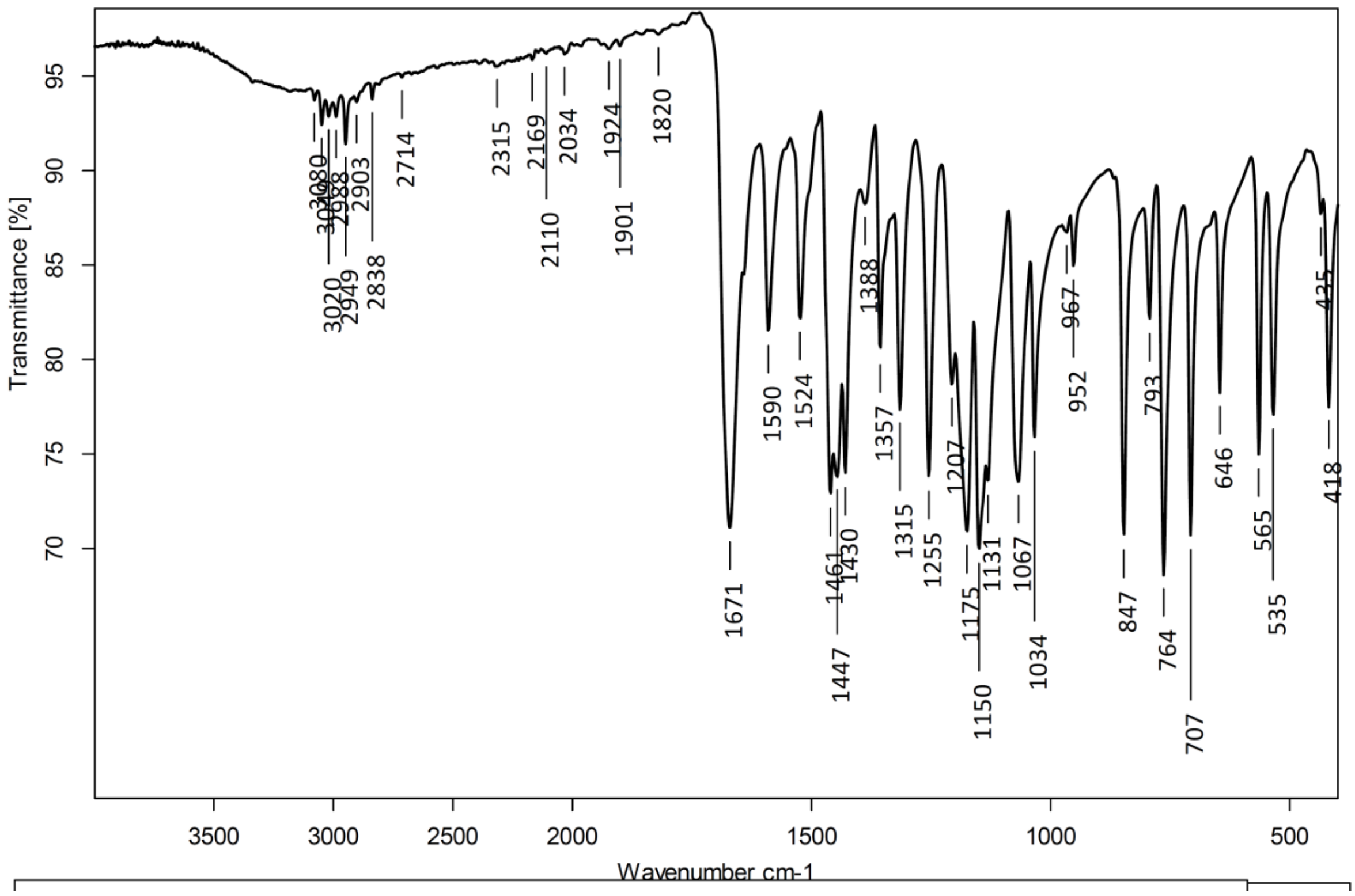


**Figure S6.** FTIR-ATR spectrum of **5**.

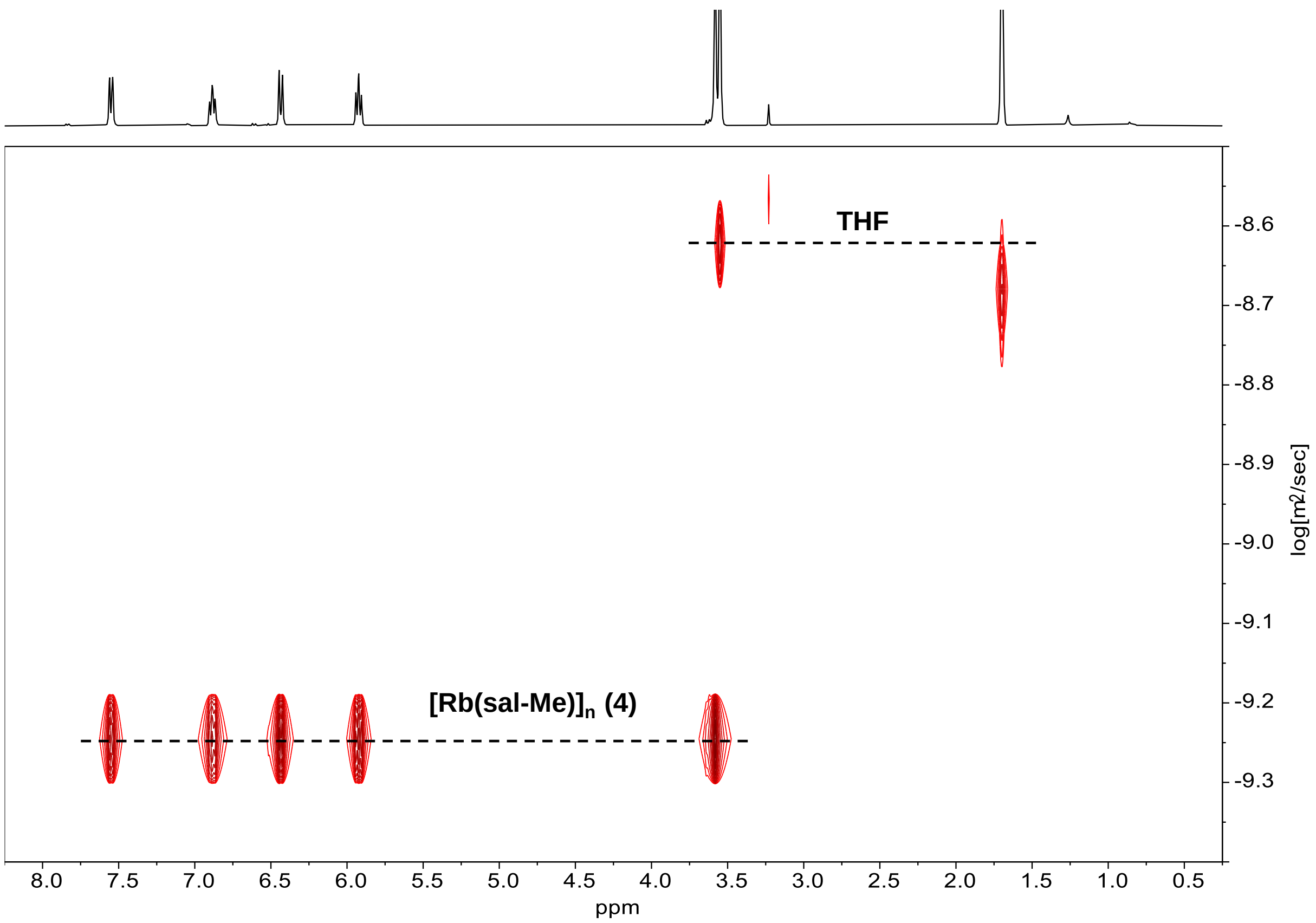


**Figure S7.** $^{1}$H-DOSY NMR spectrum of **4** in THF-$d_8$.

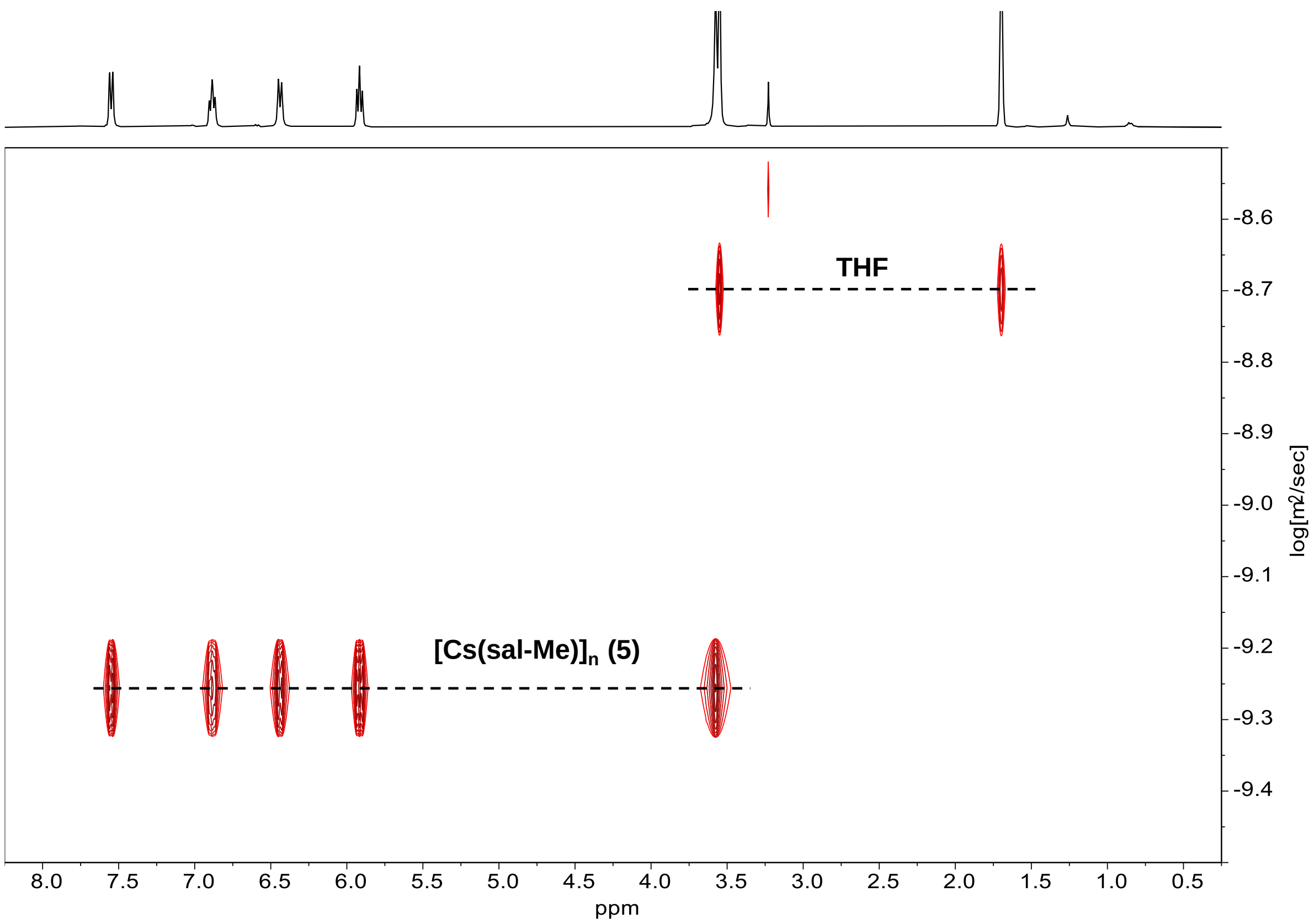


**Figure S8.** $^{1}$H-DOSY NMR spectrum of **5** in THF-$d_8$.

**Table S2.** Formula weights (FWs) and the hydrodynamic radii (rH) of **4-5**, **12-15**, **14a**, **23-26**, estimated from the Stokes-Einstein Gierer-Wirtz method.

| compound | log($D_x$) | T (°C) | FW (g/mol) | $FW_{calc}$ (g/mol) | $r_{X\text{-ray}}$ (Å) | $r_H$ (Å) |
|---|---|---|---|---|---|---|
| **4** | -9.2422 | 21.2 | $(236.61)_n$ | 1332 | 7.24 | 9.44 |
| **5** | -9.2628 | 22.8 | $(284.05)_n$ | 1568 | 7.38 | 9.97 |
| **12** | -9.1896 | 22.5 | $(590.22)_n$ | 1052 | 8.25 | 8.74 |
| **13** | -9.119<br>-9.2368 | 22.9 | 1051.54 | 740<br>1368 | 7.65 | 7.76<br>9.56 |
| **14a** | -9.0894 | 22.7 | $(1083.63)_n$ | 633 | 7.87 | 7.37 |
| **14** | -9.0840 | 23 | 1227.85 | 622 | 7.92 | 7.33 |
| **15** | -9.1447 | 23.2 | 1260.06 | 852 | - | 8.14 |
| **23** | -9.2345 | 20.8 | 828.00 | 1255 | 7.40 | 9.26 |
| **24** | -9.1262 | 20.7 | $(715.93)_n$ | 717 | 6.37 | 7.68 |
| **25** | -9.1855 | 21.4 | $(748.14)_n$ | 994 | - | 8.57 |
| **26** | -9.3365 | 19.8 | $(228.17)_n$ | 2120 | 8.67 | 11.03 |

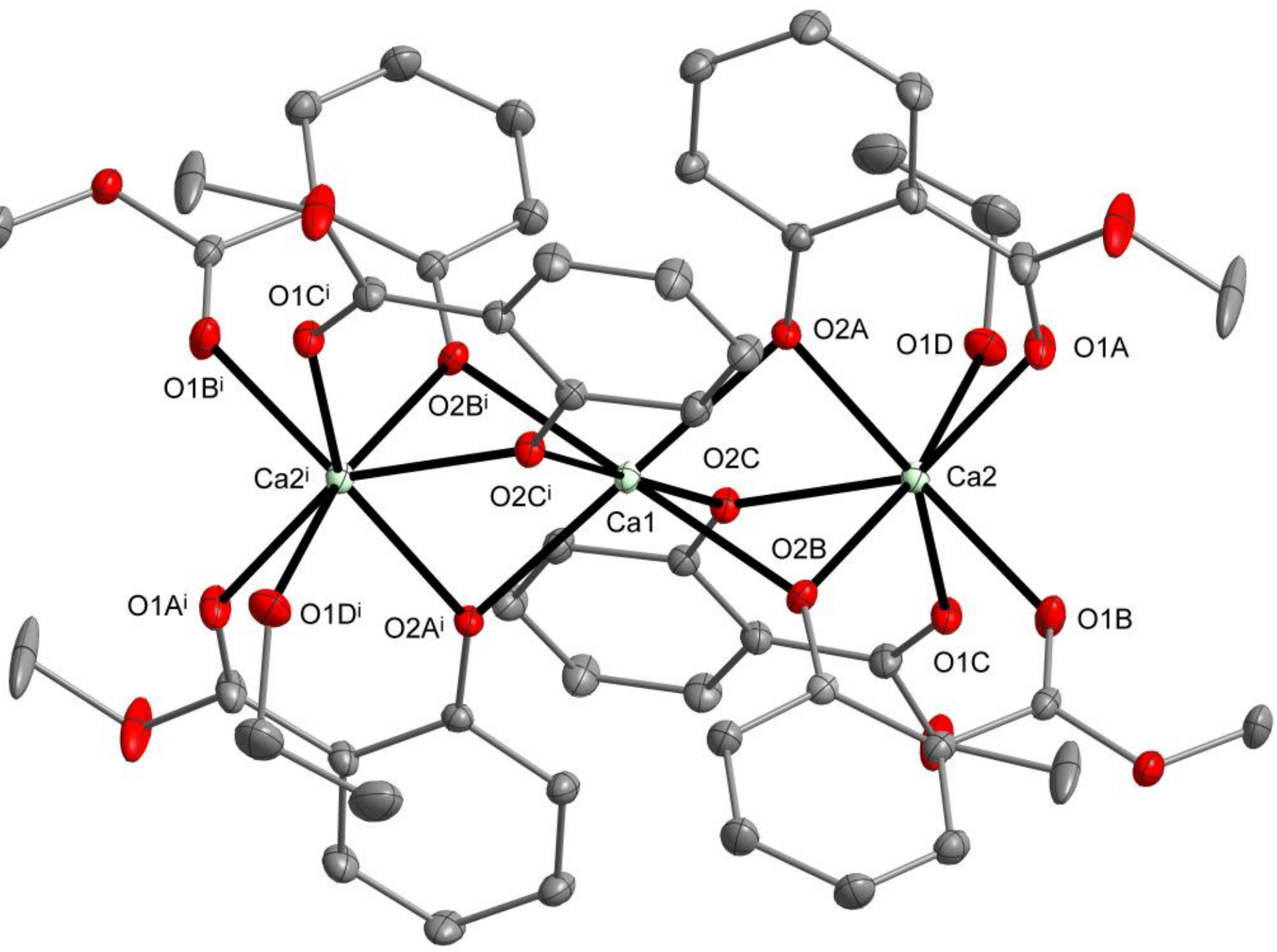


**Figure S9.** The molecular structure of $[Ca_3(\text{sal-Me})_6(EtOH)_2]$ (**8**).

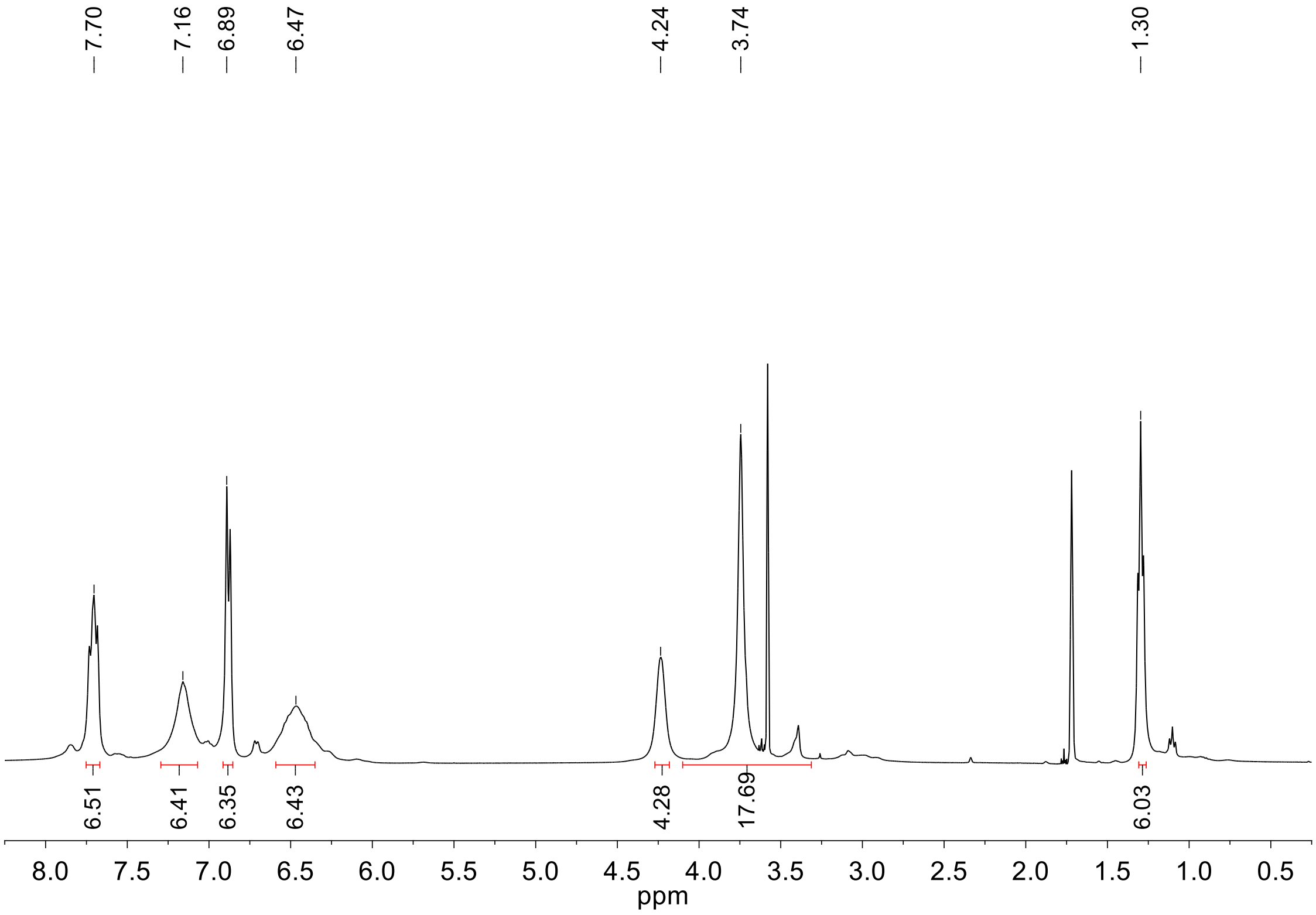


**Figure S10.** $^1$H NMR spectrum of **8** in THF-$d_8$.

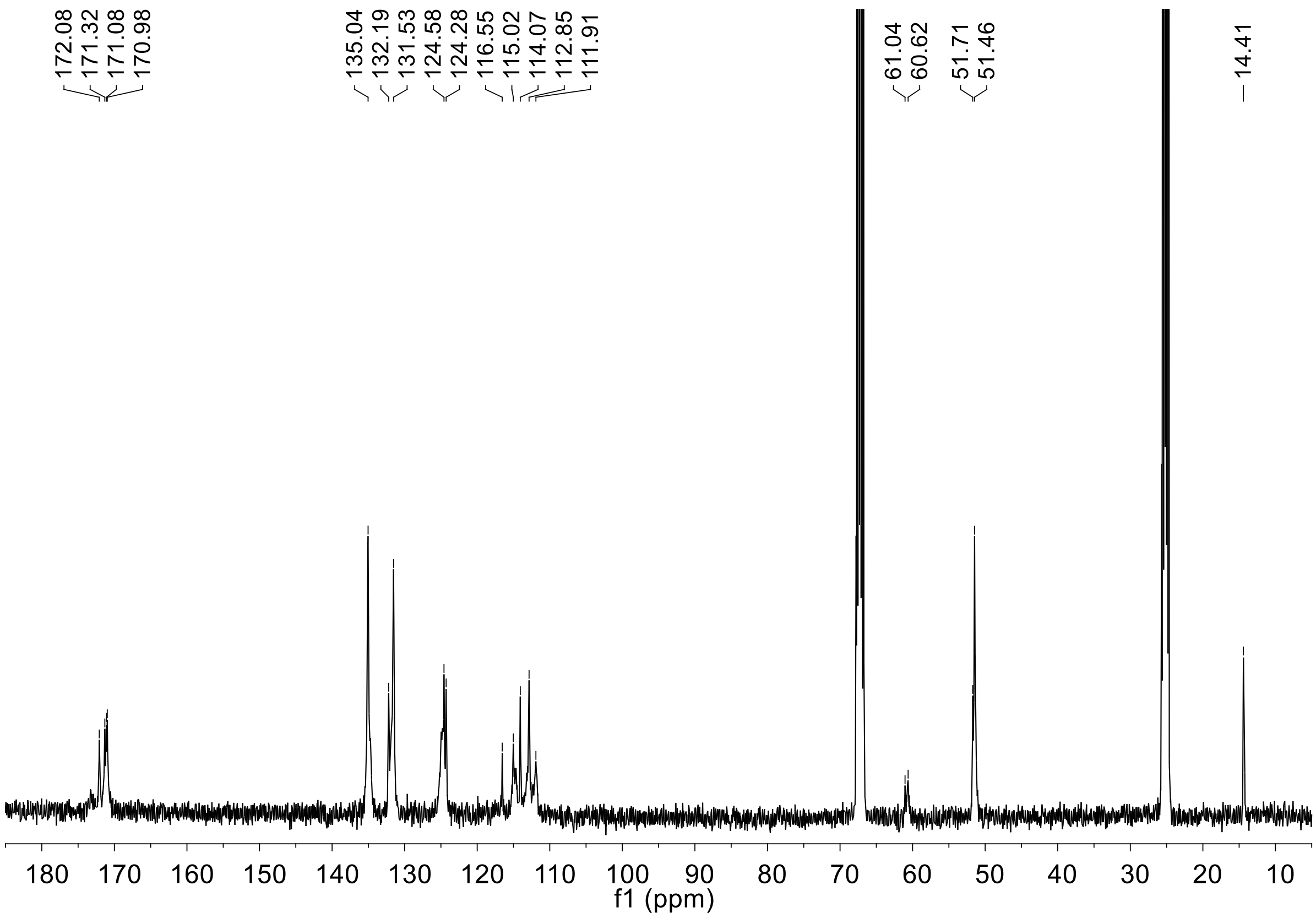


**Figure S11.** $^{13}$C NMR spectrum of **8** in THF-$d_8$.

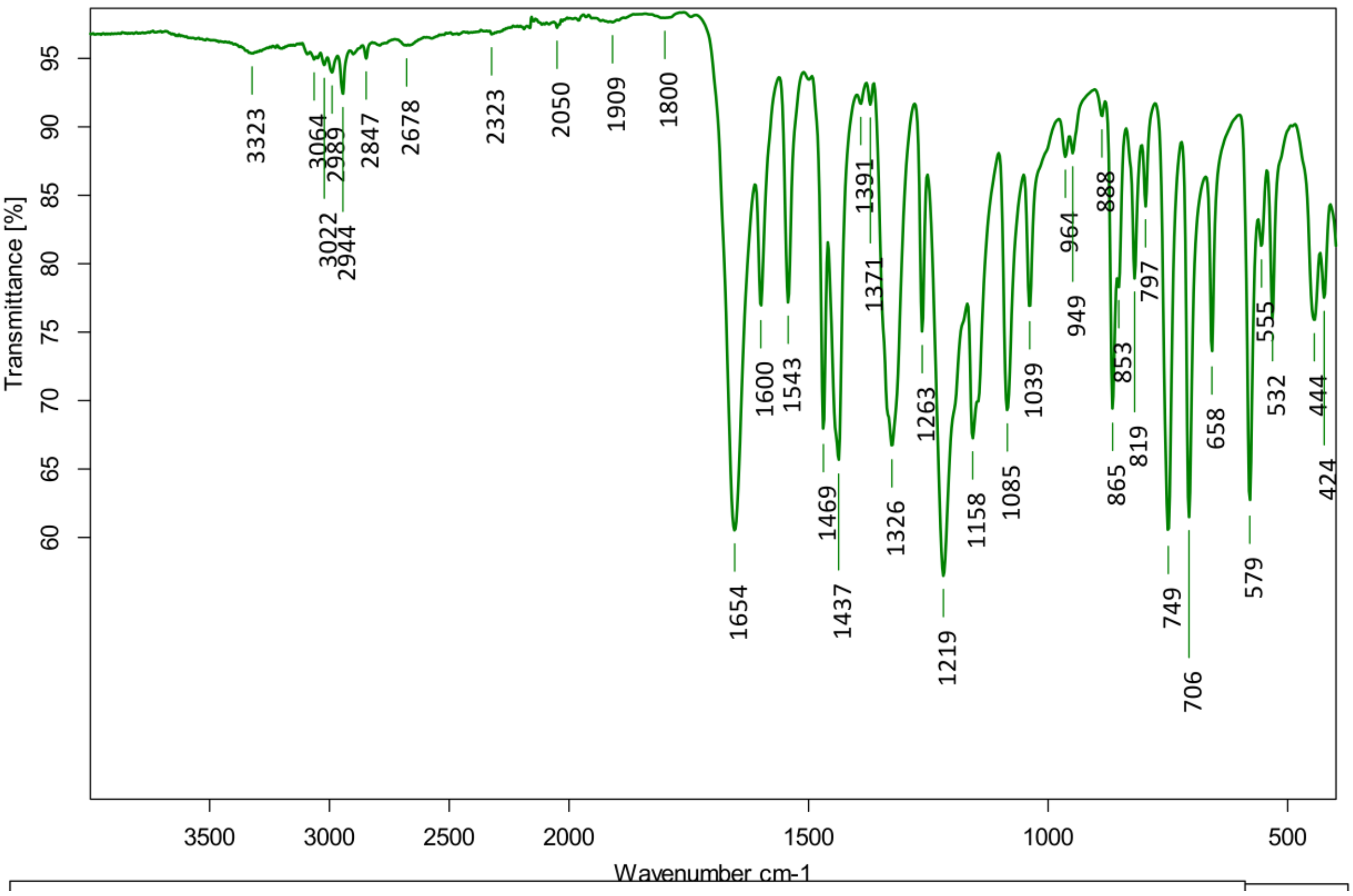


**Figure S12.** FTIR-ATR spectrum of **8**.

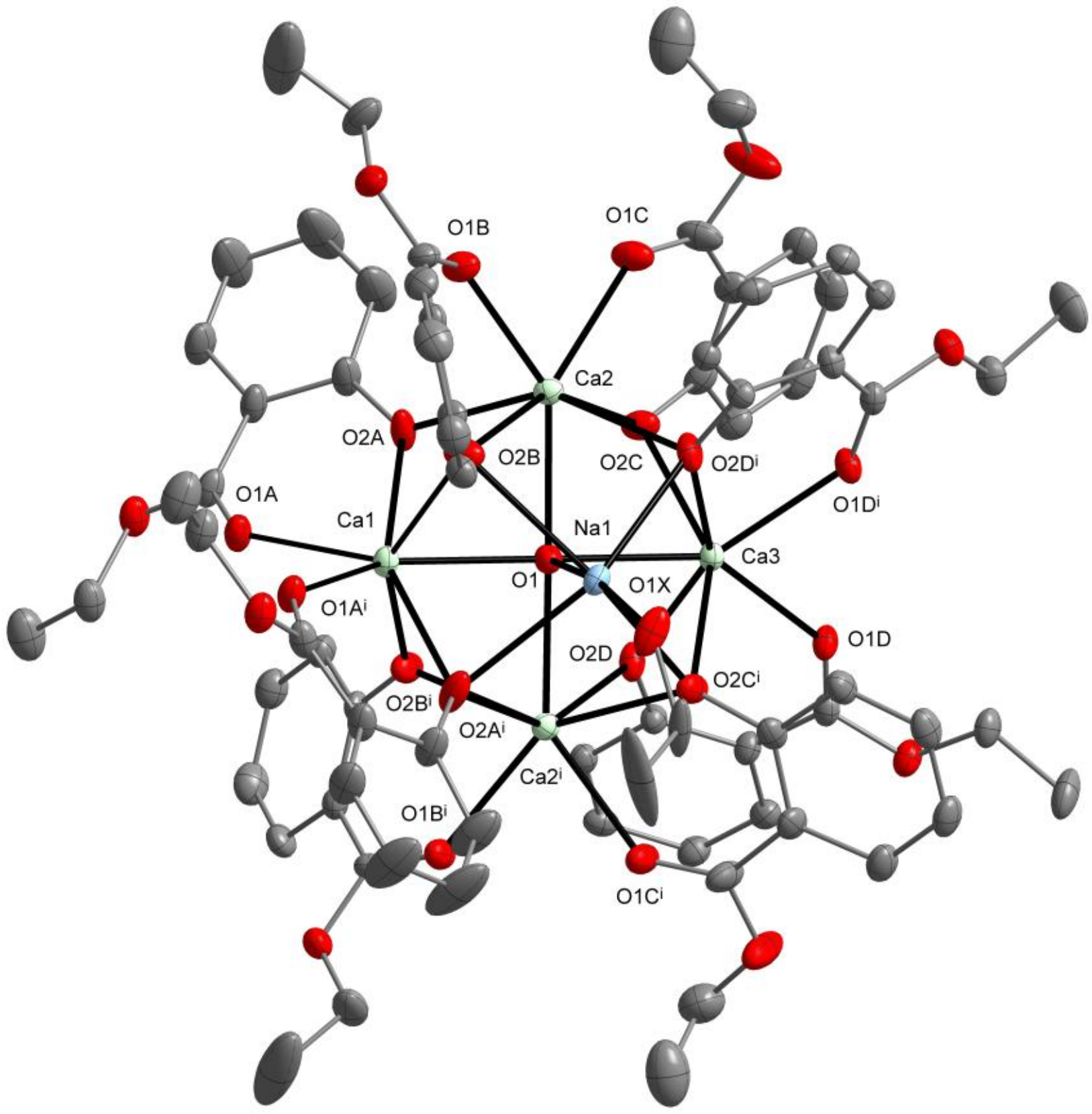


**Figure S13.** The molecular structure of [$Ca_4Na(\mu_5\text{-}OH)(sal\text{-}Et)_8(EtOH)$] (**21**).

**Table S3.** Continuous-shape measurements (CShM) of the coordination environment around M ions.

| compound | atom | donor atoms | polyhedron | S parameter |
|---|---|---|---|---|
| **8** | Ca1 | $O_6$ | octahedron | 2.471 |
| | Ca2 | $O_7$ | capped trigonal prism | 1.842 |
| | Ca2i | $O_7$ | capped trigonal prism | 1.840 |
| **12** | Mg | $O_6$ | octahedron | 0.749 |
| | Rb | $O_8$ | biaugmented trigonal prism | 8.290 |
| **14a** | Zn1 | $O_5$ | square pyramid | 0.482 |
| | Na1 | $O_6$ | octahedron | 2.551 |
| **23** | Zn | $O_4$ | tetrahedron | 4.917 |
| | Li1 | $O_5$ | square pyramid | 1.491 |
| | Li2 | $O_5$ | square pyramid | 0.397 |
| **24** | Zn1 | $O_5$ | tetrahedron | 2.851 |
| | Na1 | $O_5$ | square pyramid | 4.348 |
| | Na2 | $O_5$ | square pyramid | 4.347 |
| **26** | Li1 | $O_4$ | tetrahedron | 1.454 |
| | Li2 | $O_4$ | tetrahedron | 1.230 |
| | Li3 | $O_4$ | tetrahedron | 2.498 |

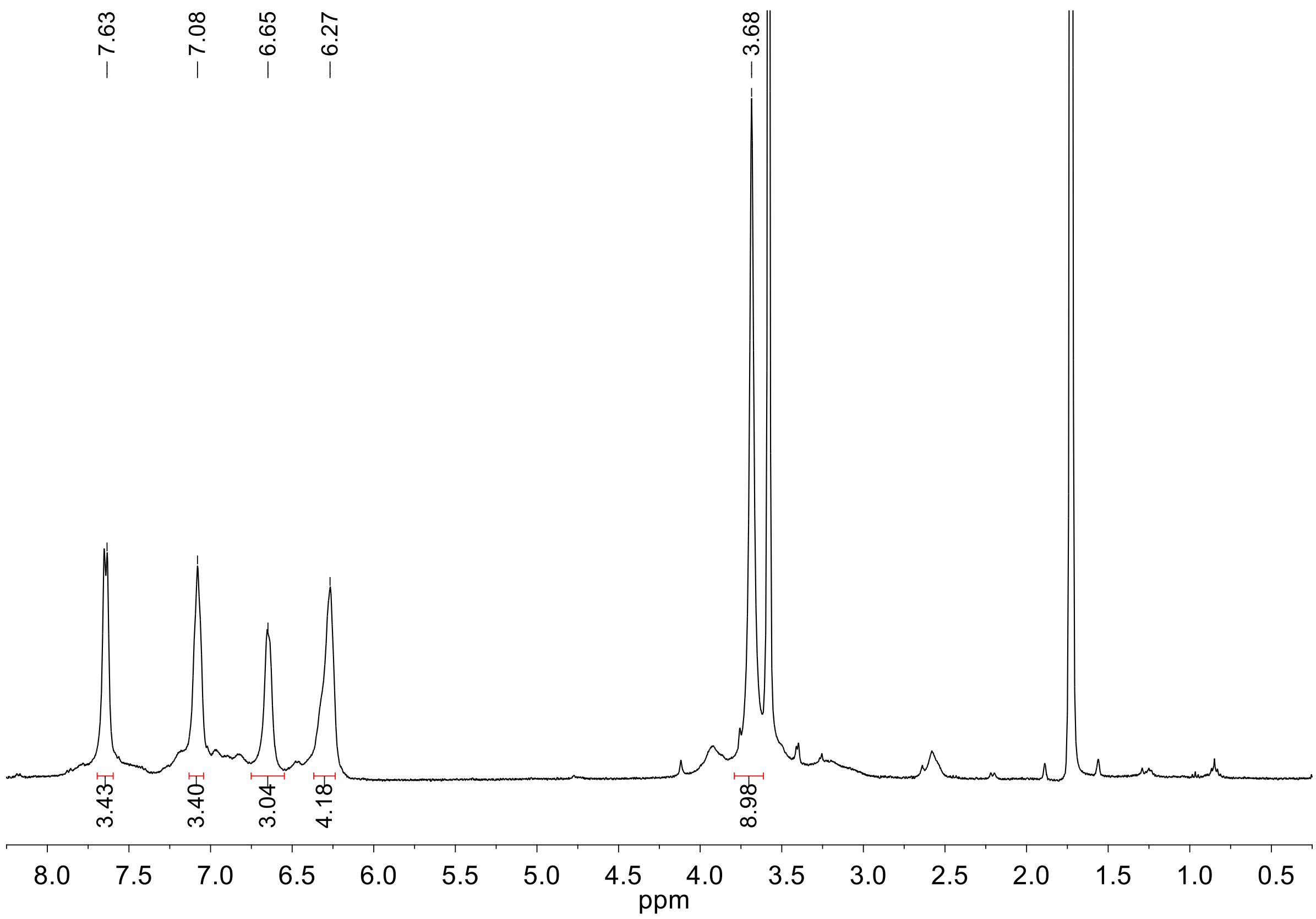


**Figure S14.** $^1$H NMR spectrum of **12** in THF-$d_8$.

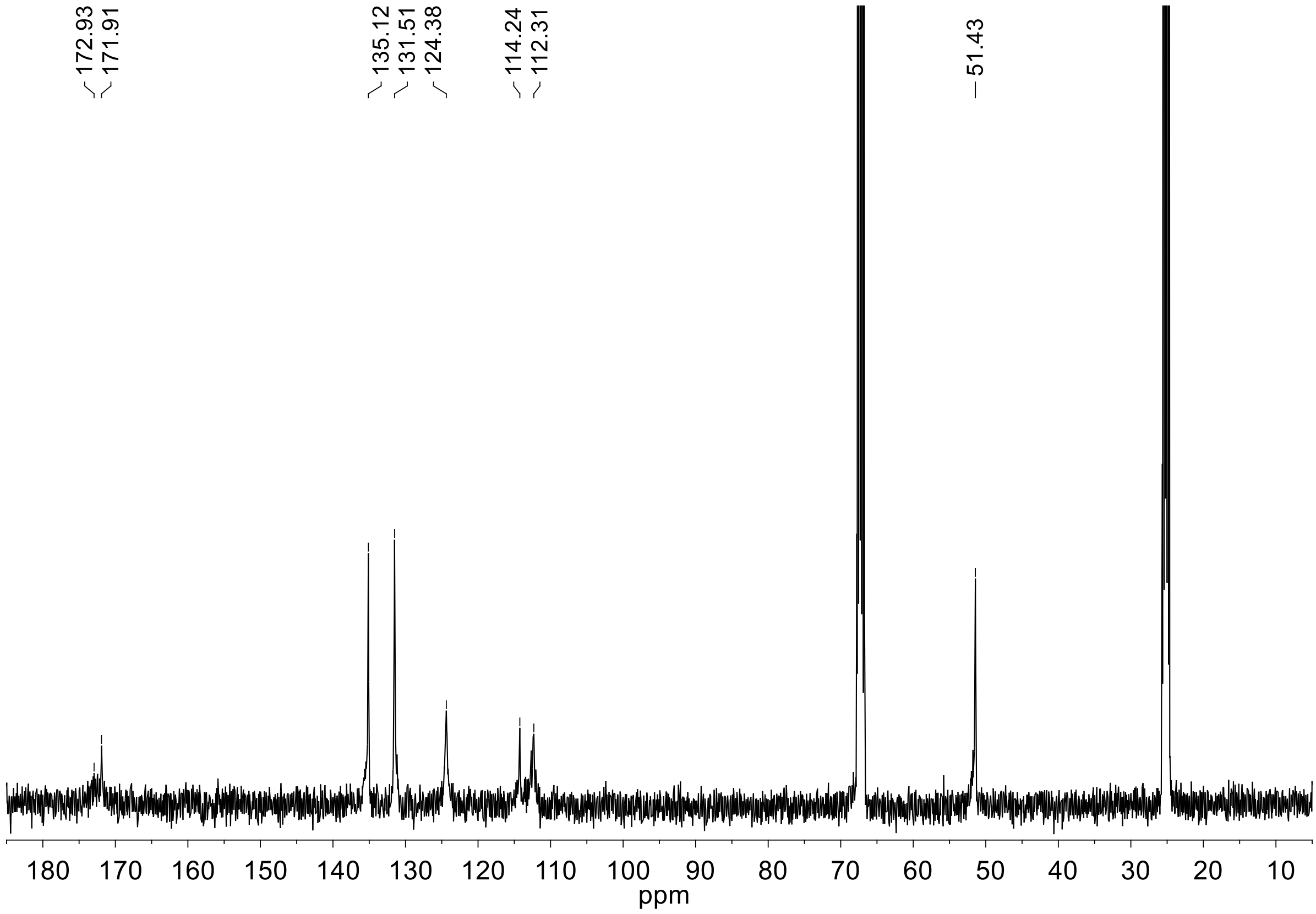


**Figure S15.** $^{13}C$ NMR spectrum of **12** in THF-$d_8$.

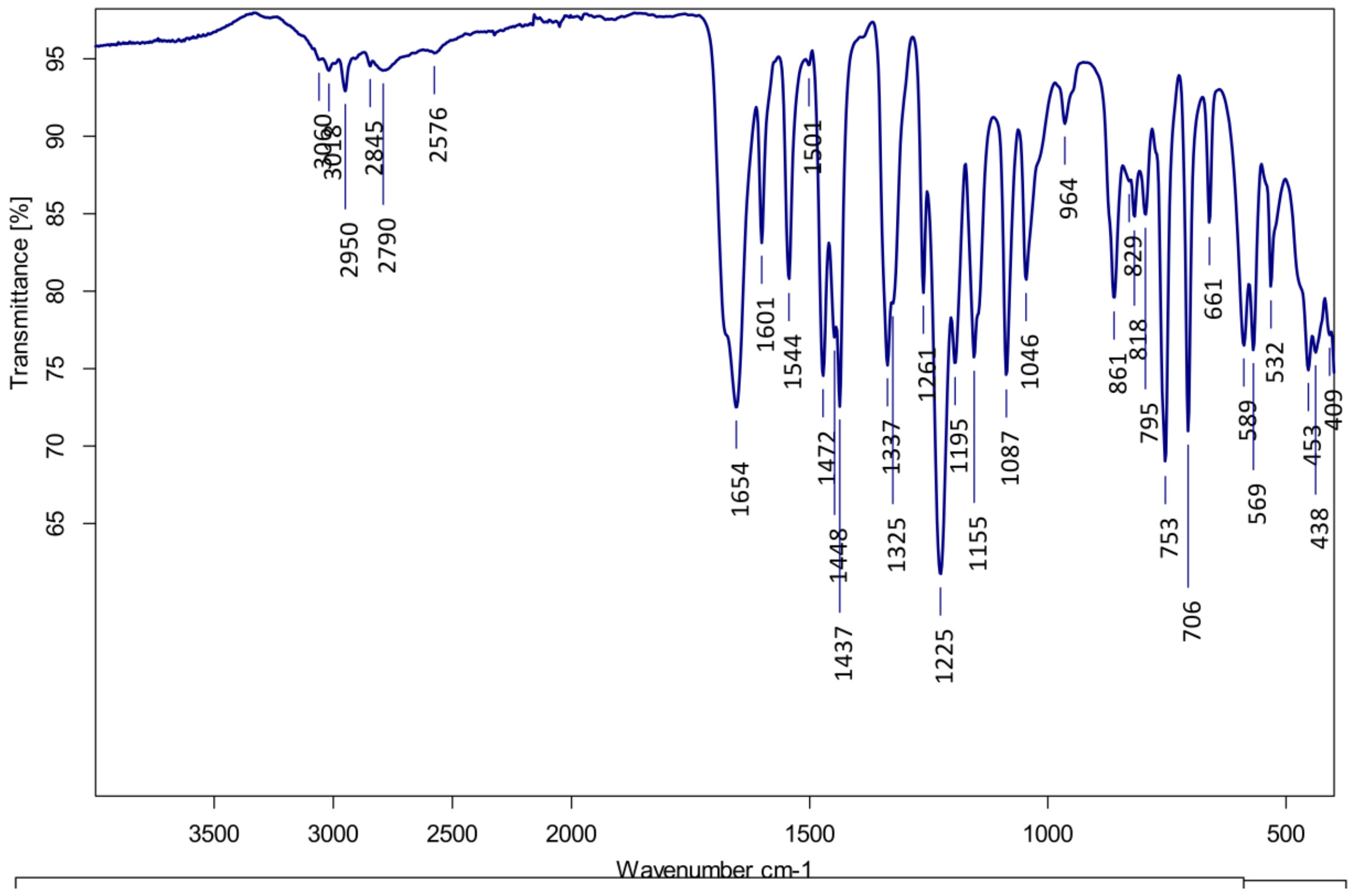


**Figure S16.** FTIR-ATR spectrum of **12**.

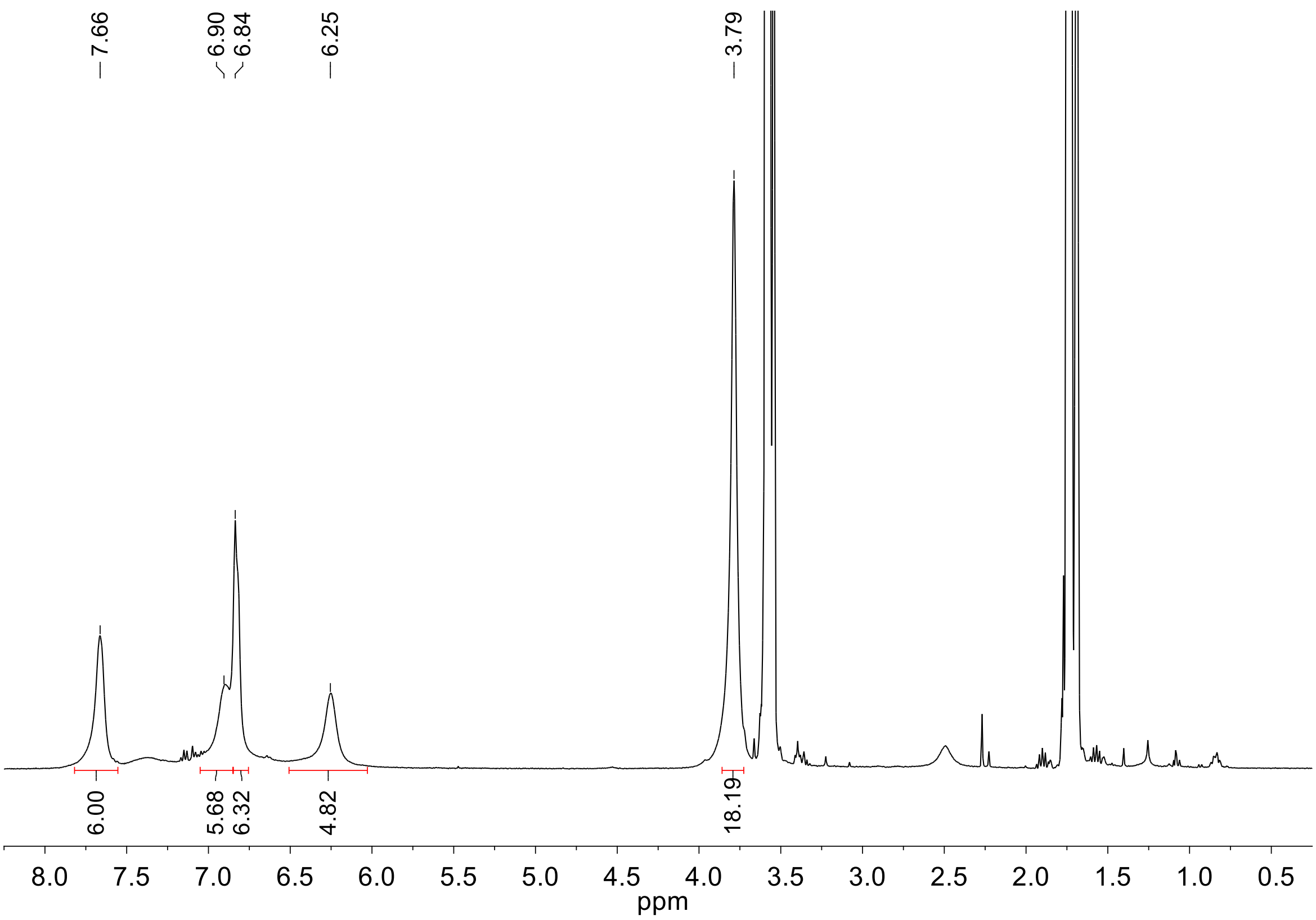


**Figure S17.** $^{1}$H NMR spectrum of **14a** in THF-$d_8$.

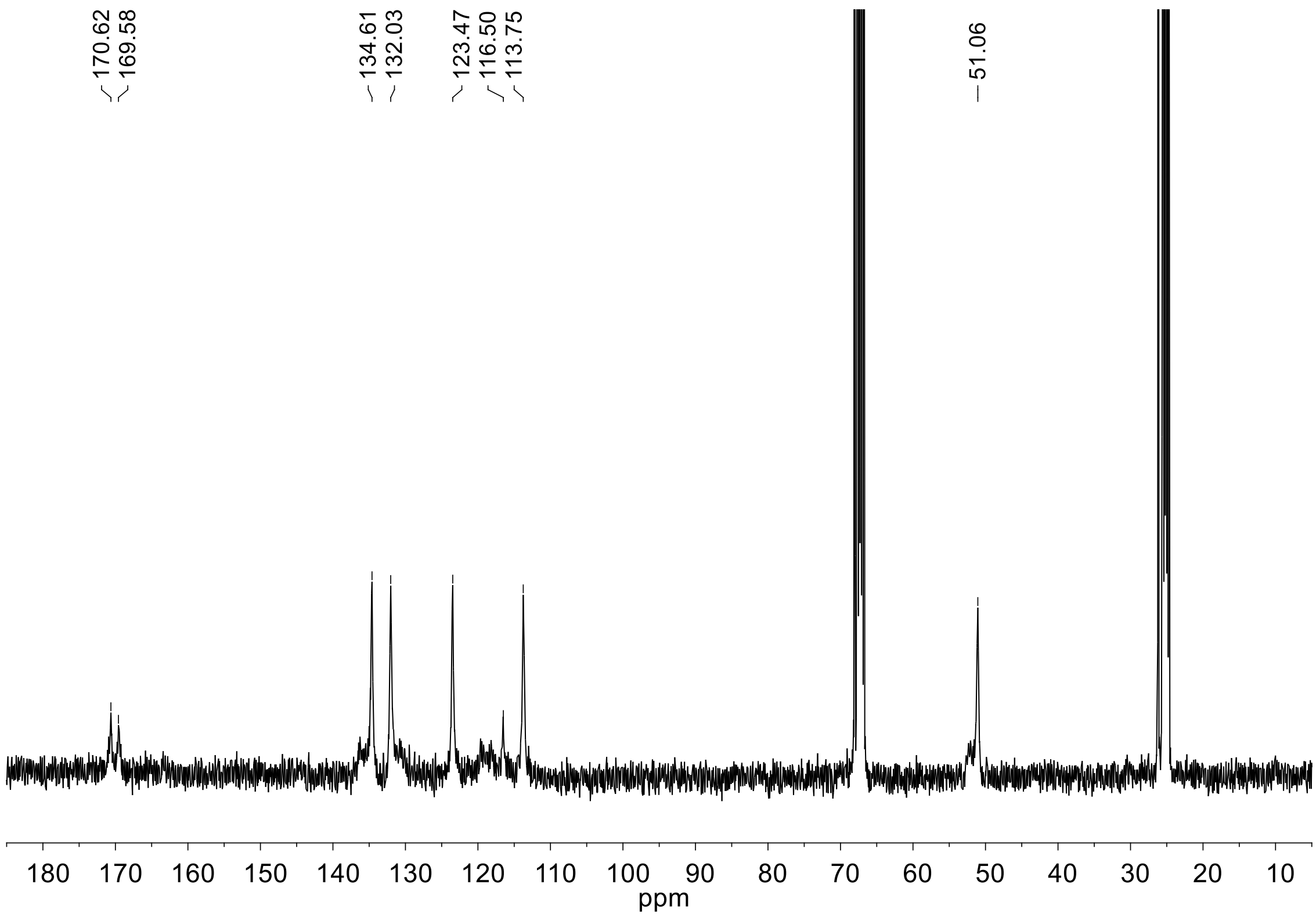


**Figure S18.** $^{13}$C NMR spectrum of **14a** in THF-$d_8$.

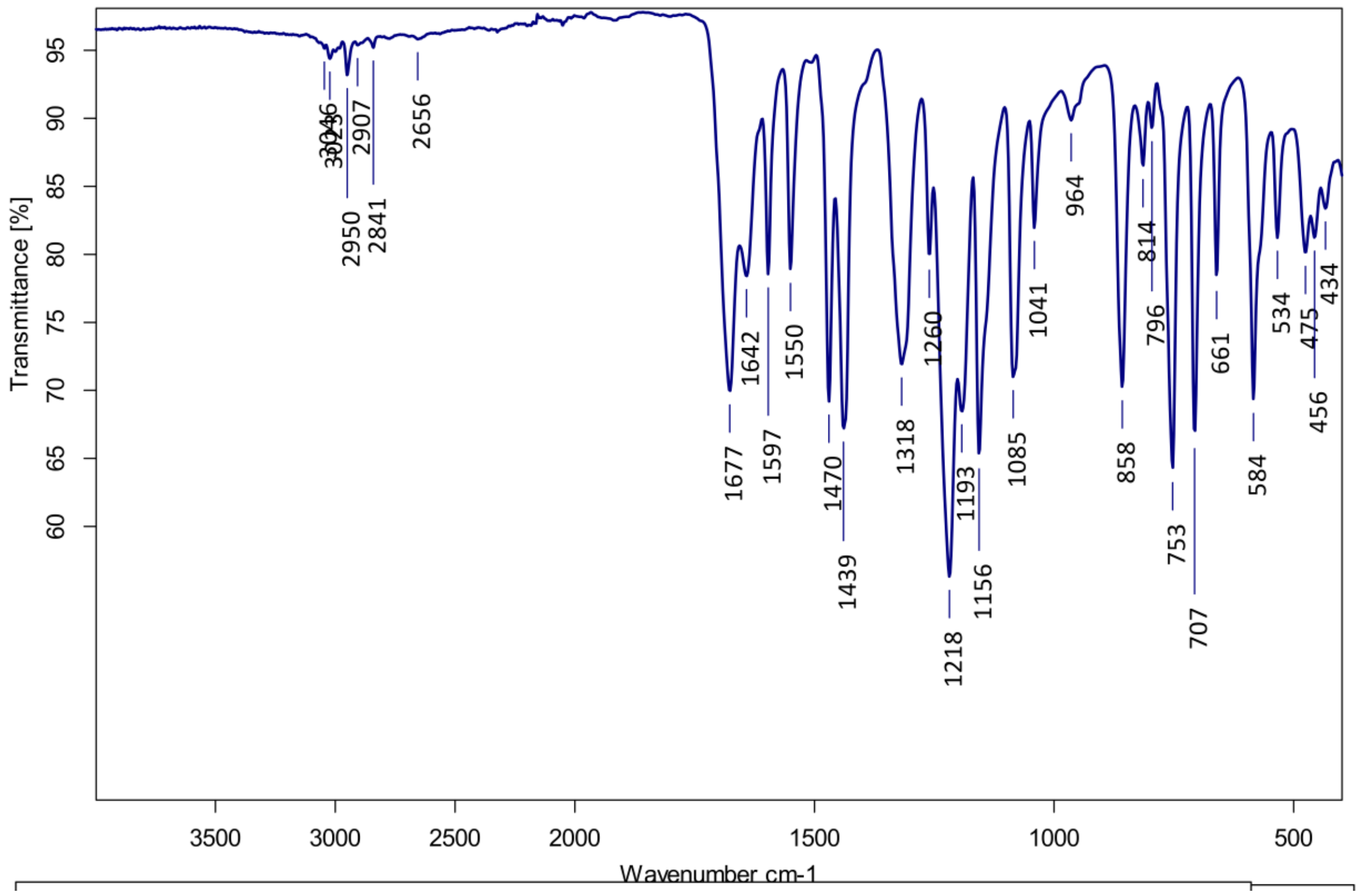


**Figure S19.** FTIR-ATR spectrum of **14a**.

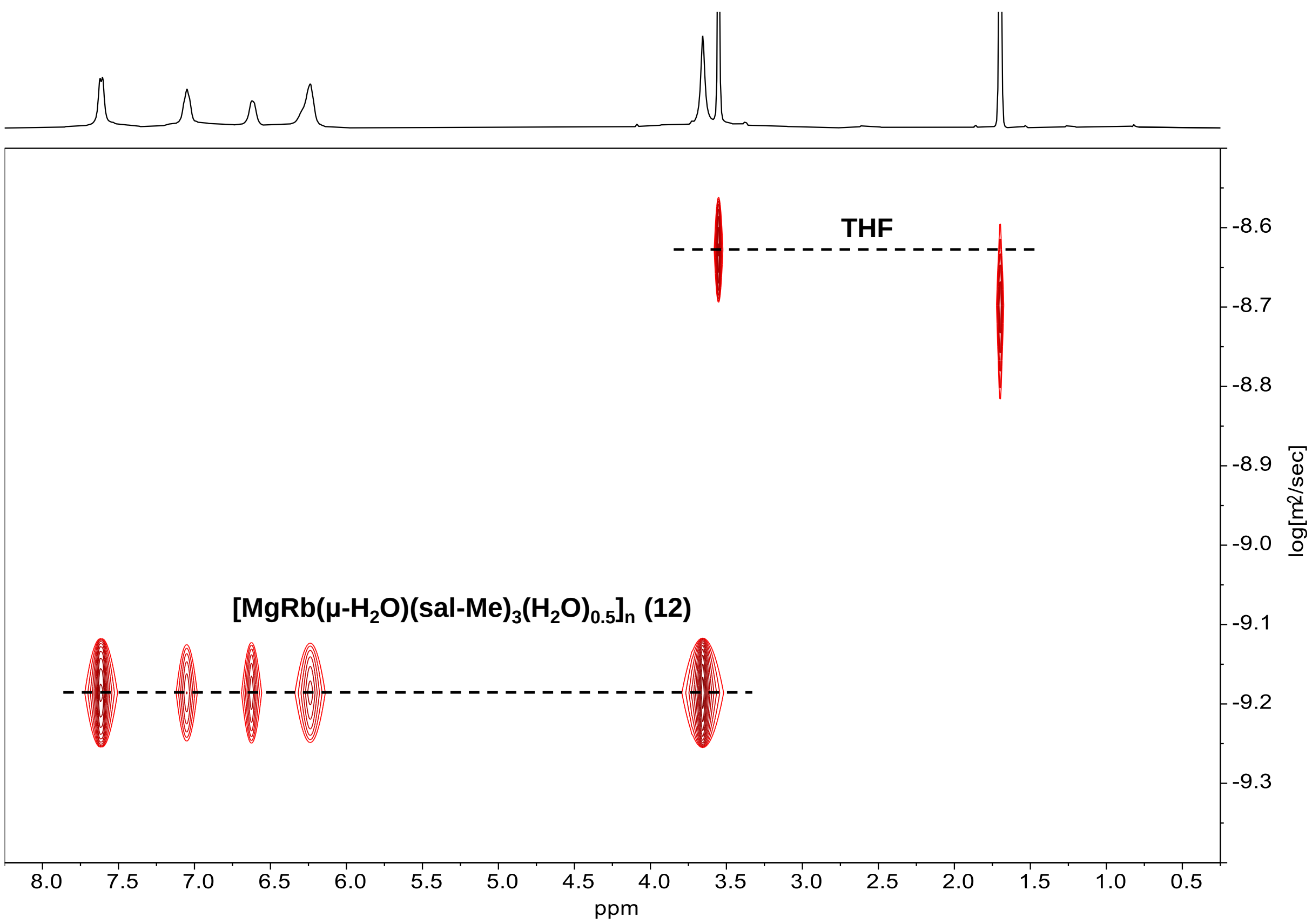


**Figure S20.** $^{1}$H-DOSY NMR spectrum of **12** in THF-$d_8$.

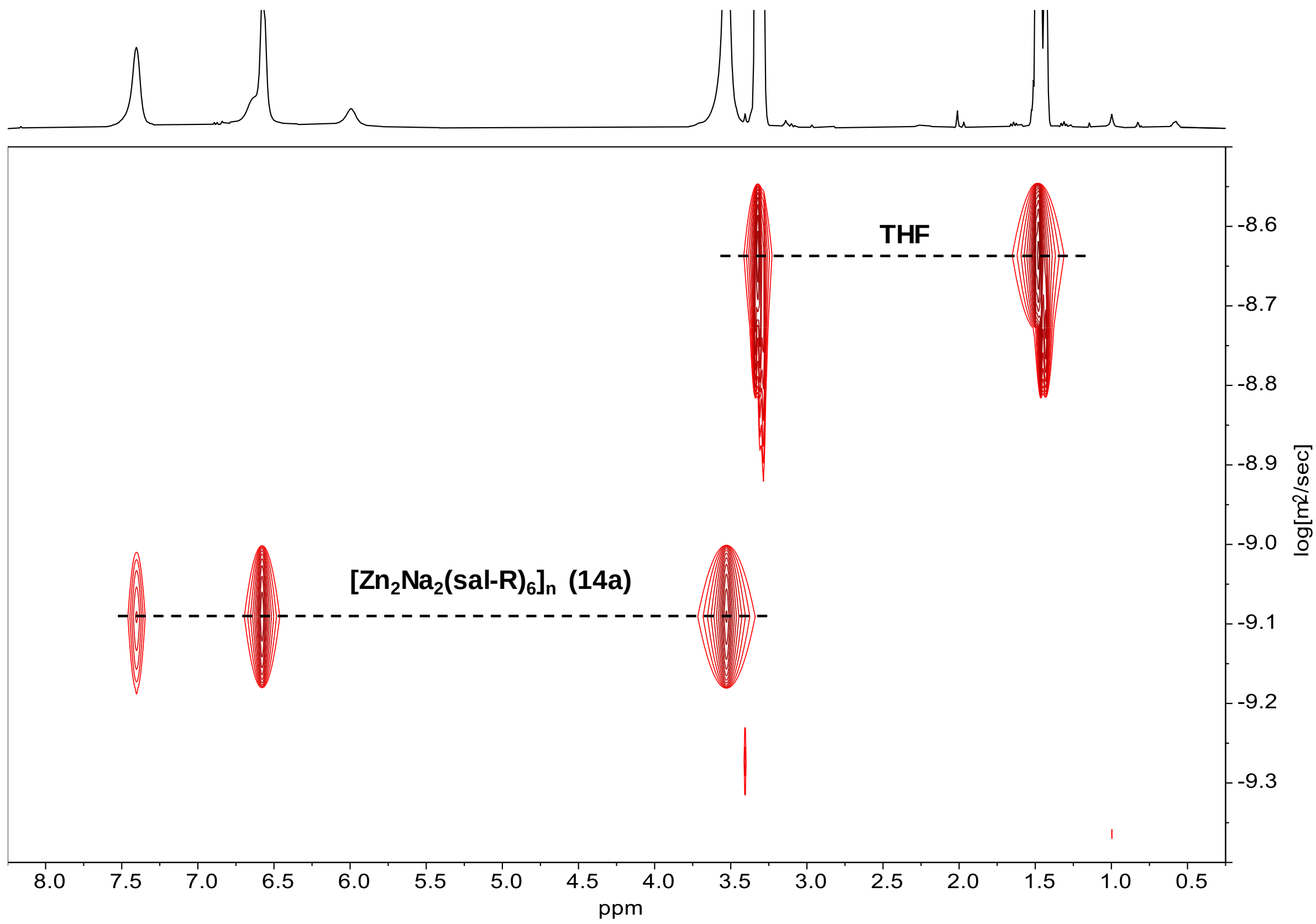


**Figure S21**.$^{1}$H-DOSY NMR spectrum of **14a** in THF-d8.

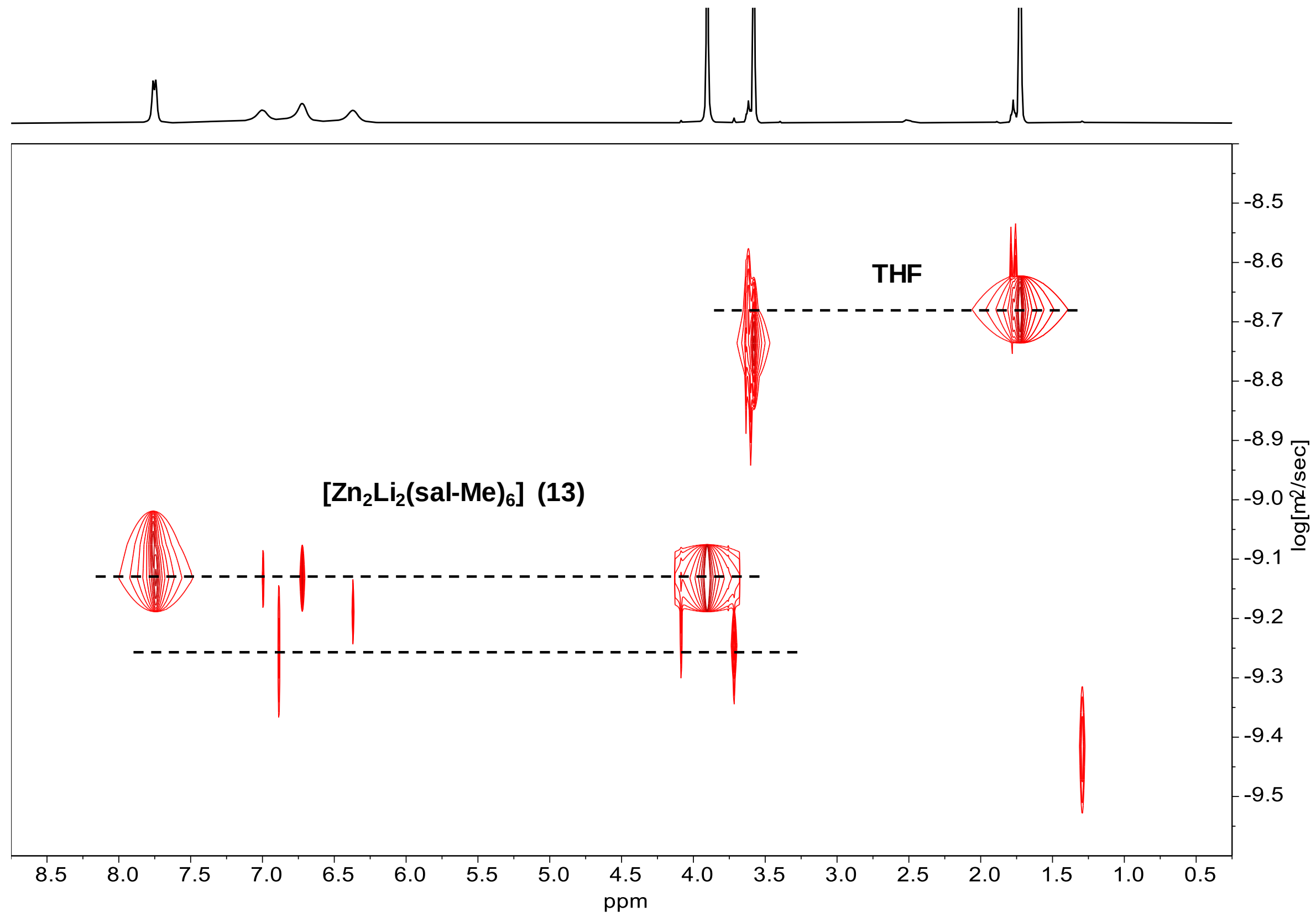


**Figure S22.** $^{1}$H-DOSY NMR spectrum of **13** in THF-d8.

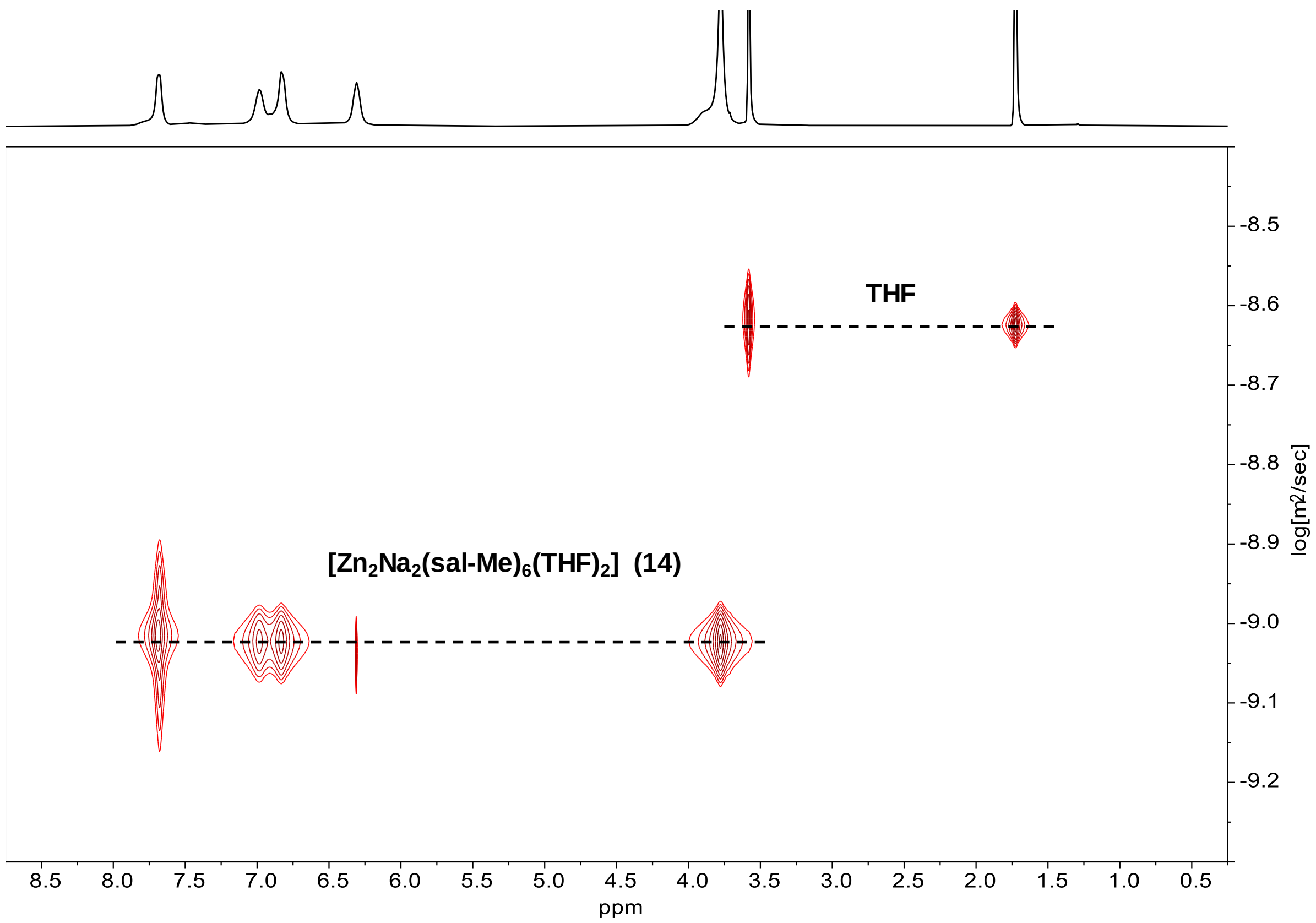


**Figure S23.** $^{1}$H-DOSY NMR spectrum of **14** in THF-$d_8$.

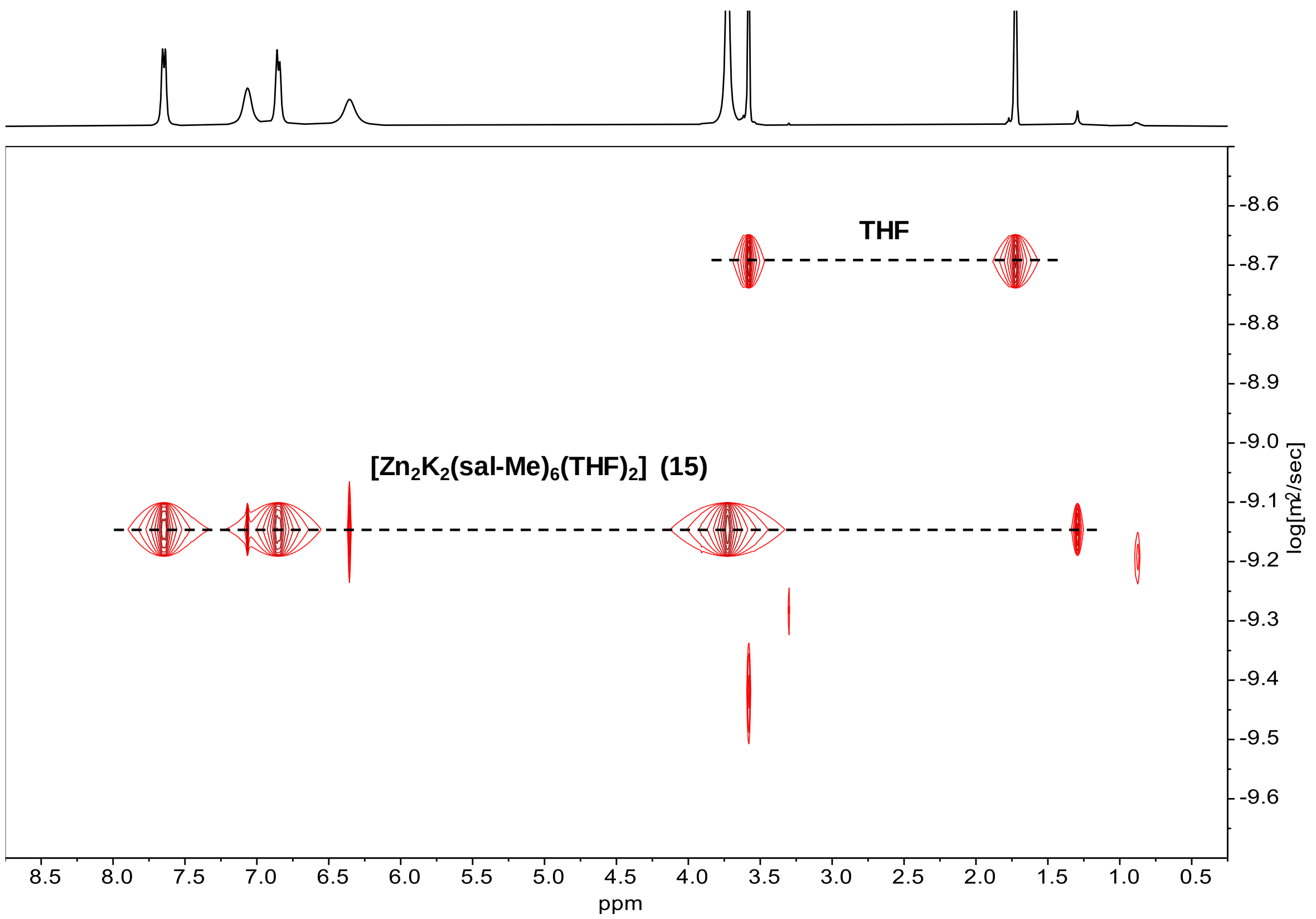


**Figure S24.** $^{1}$H-DOSY NMR spectrum of **15** in THF-$d_8$.

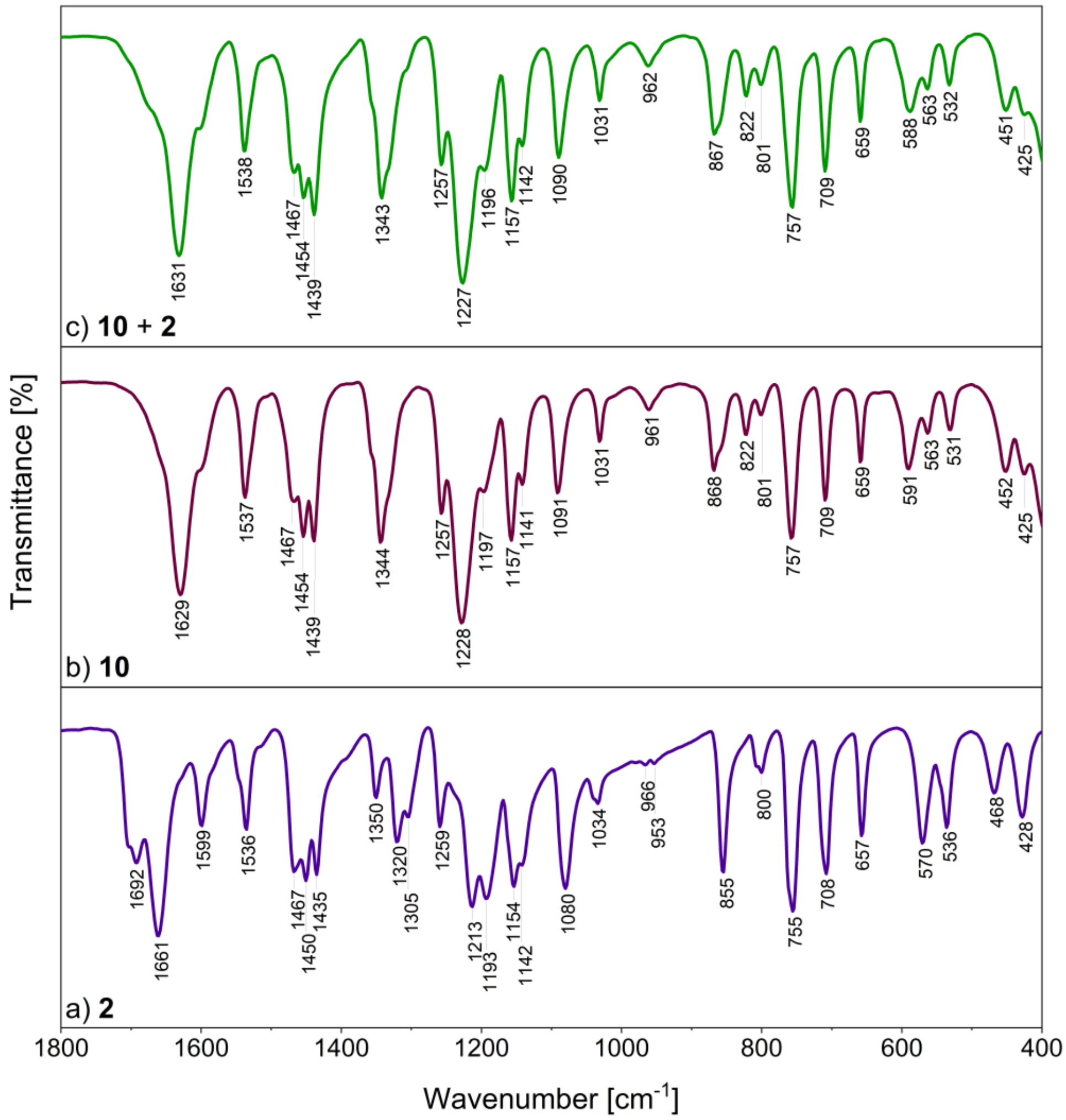


**Figure S25.** Comparison of FTIR-ATR spectra of a) **2**, b) **10**, and c) **10+2**.

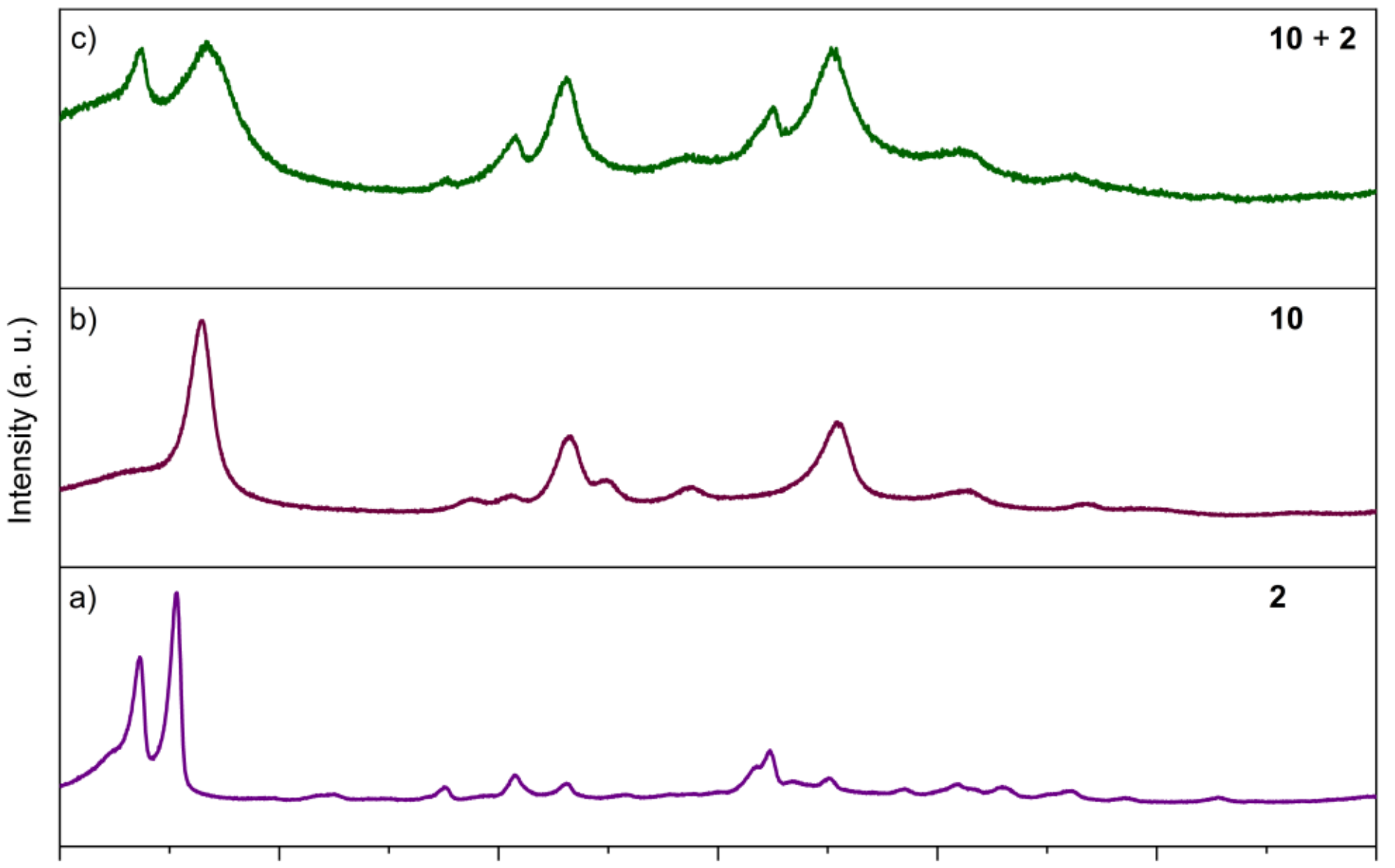


**Figure S26.** Comparison of PXRD patterns of a) **2**, b) **10**, and c) **10+2**.

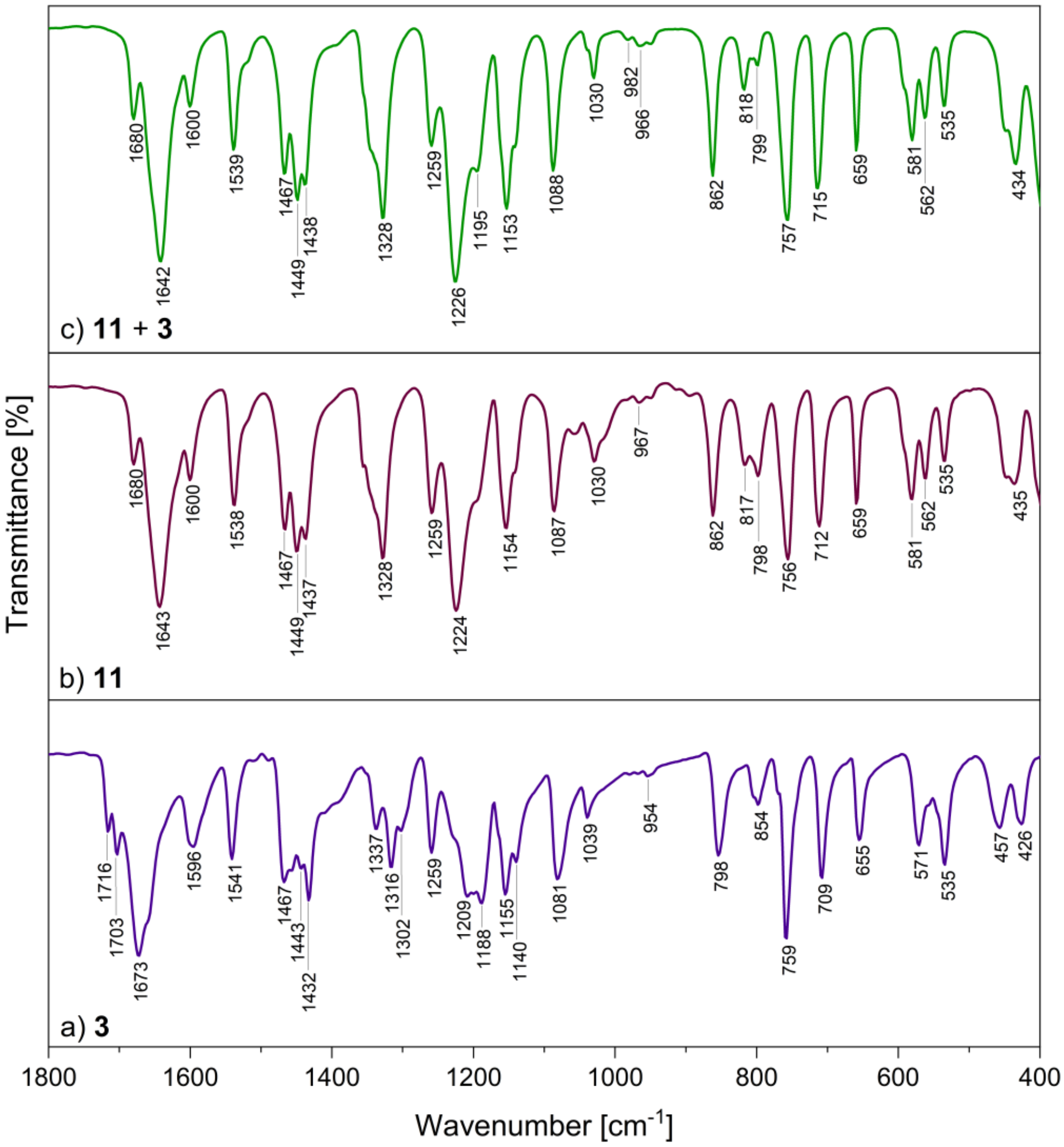


**Figure S27.** Comparison of FTIR-ATR spectra of a) **3**, b) **11**, and c) **11+23**.

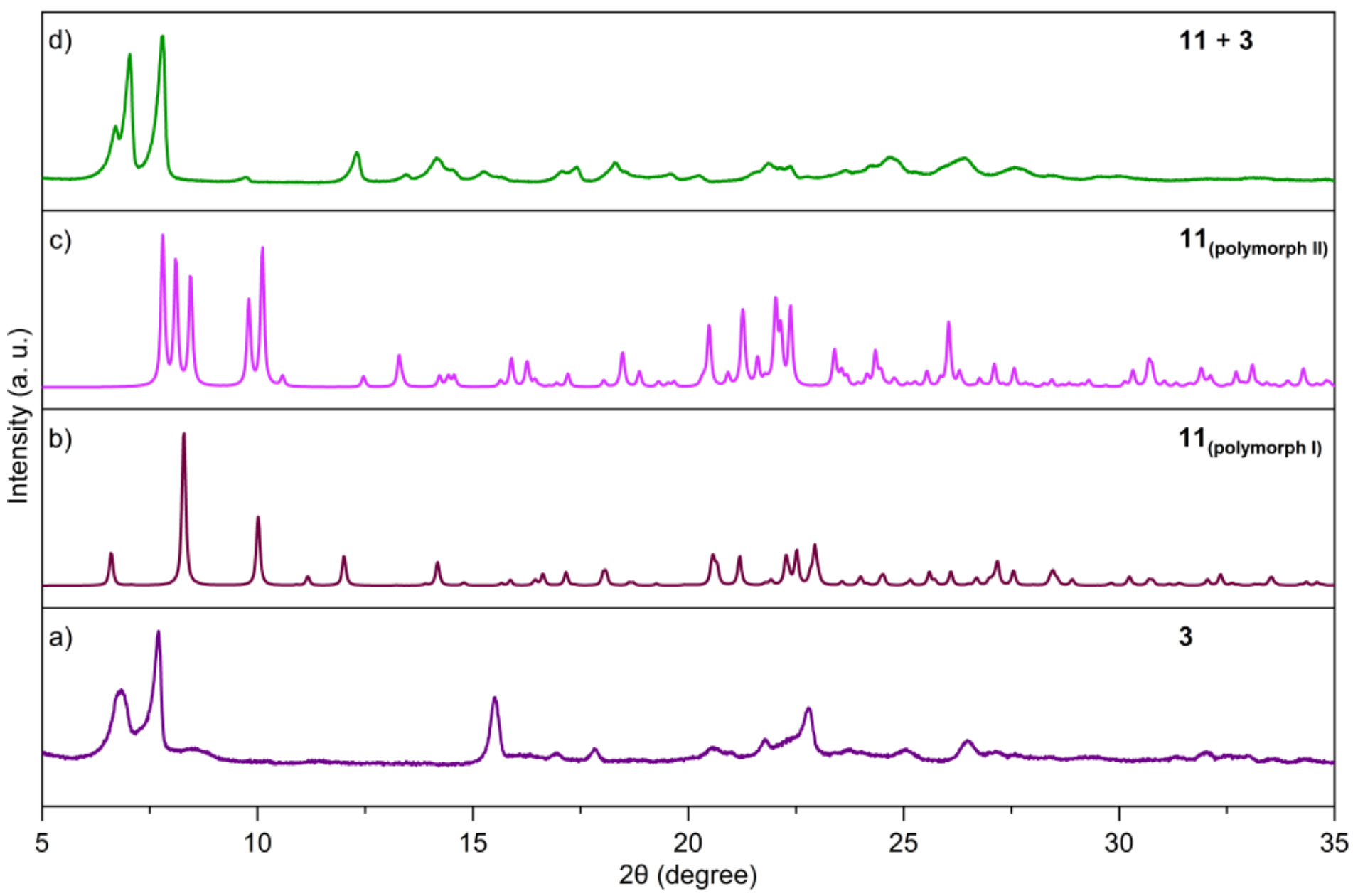


**Figure S28.** Comparison of PXRD patterns of a) **3**, b) **11(polymorph I)**, c) **11(polymorph II)**, and d) **11+23**.

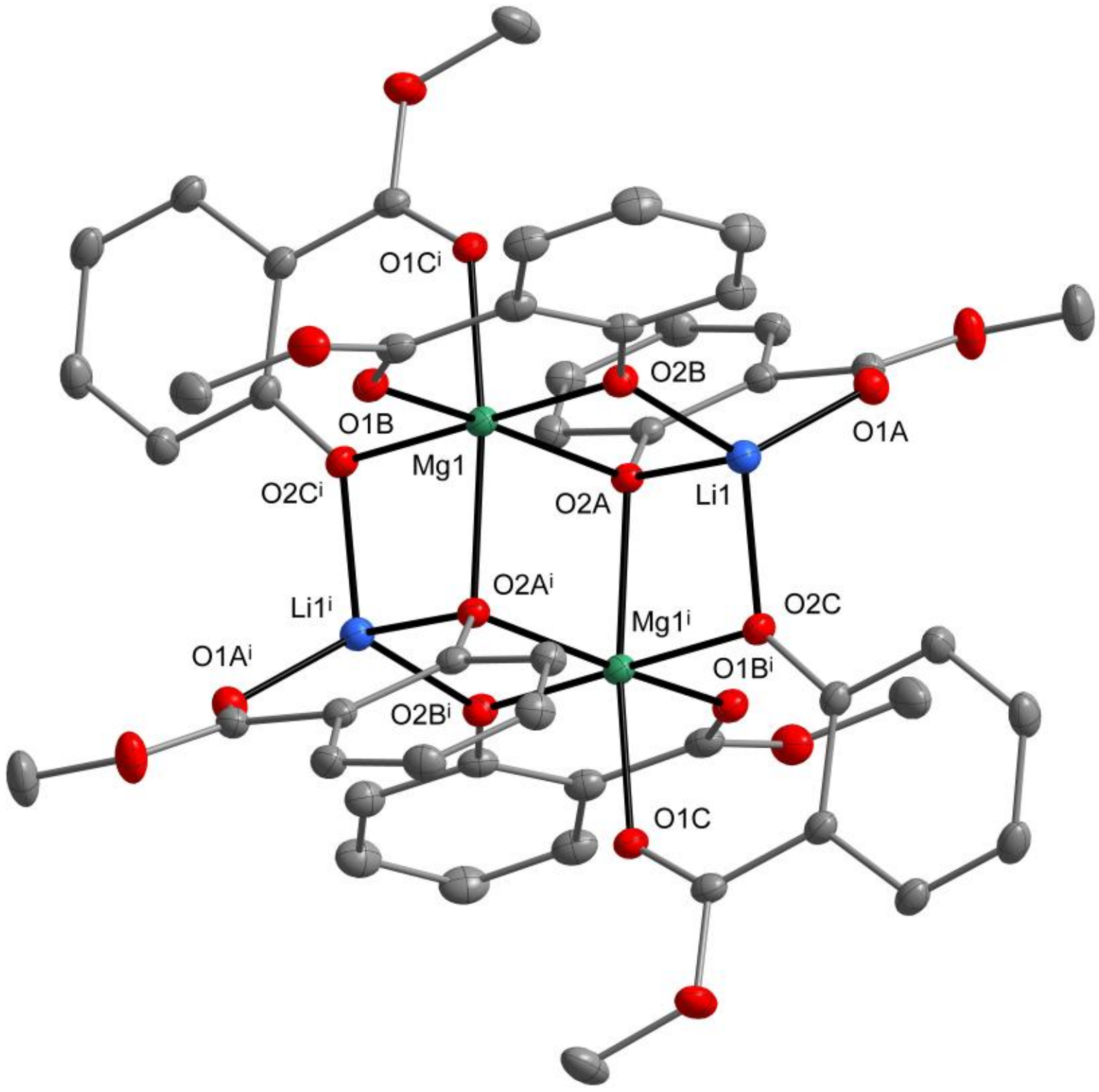


**Figure S29.** The molecular structure of $[Mg_2Li_2(sal\text{-}Me)_6]$ (**9**).

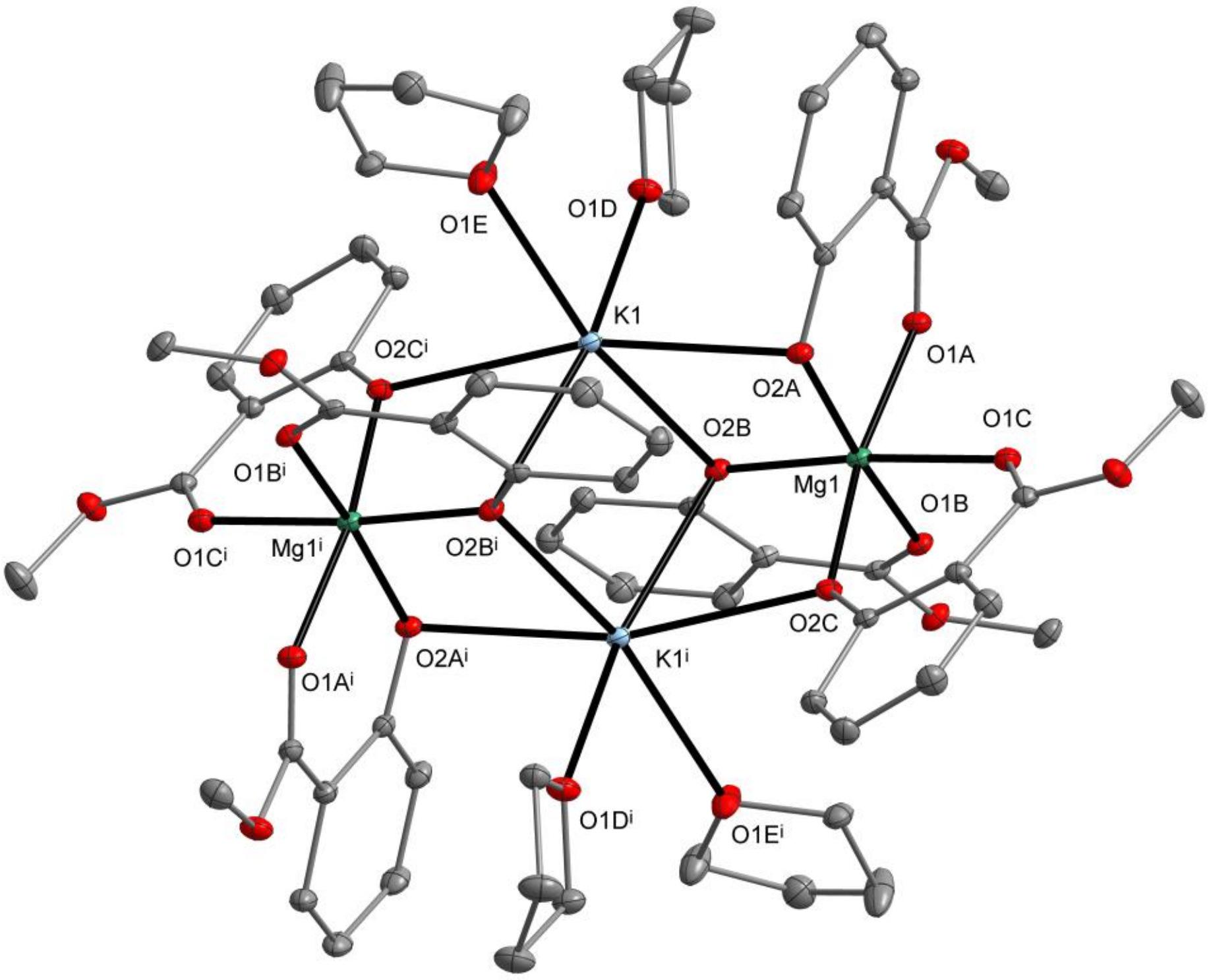


**Figure S30.** The molecular structure of $[Mg_2K_2(sal\text{-}Me)_6(THF)_4]$ (**11**).

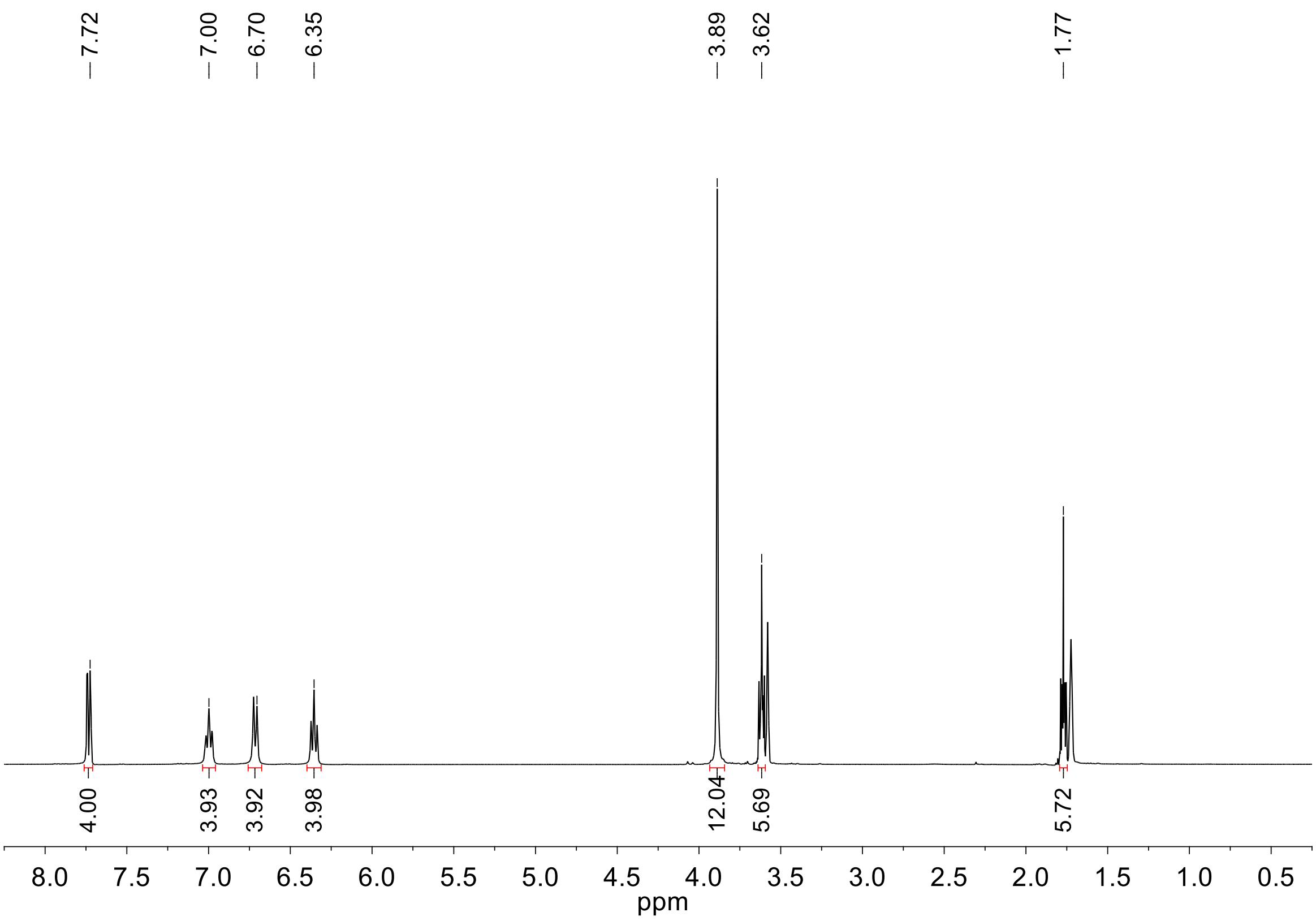


**Figure S31.** $^{1}$H NMR spectrum of **23** in THF-$d_8$.

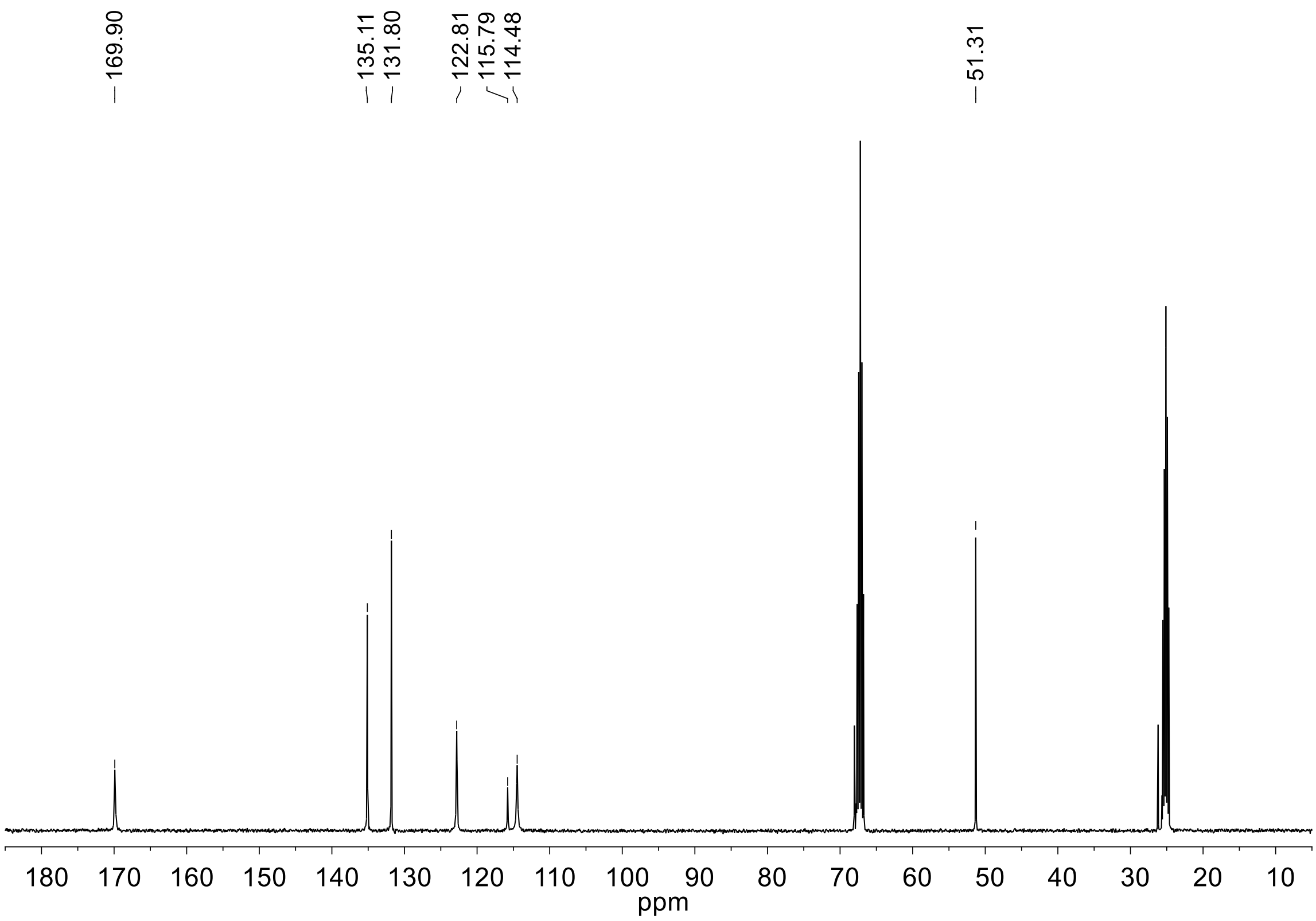


**Figure S32.** $^{13}$C NMR spectrum of **23** in THF-$d_8$.

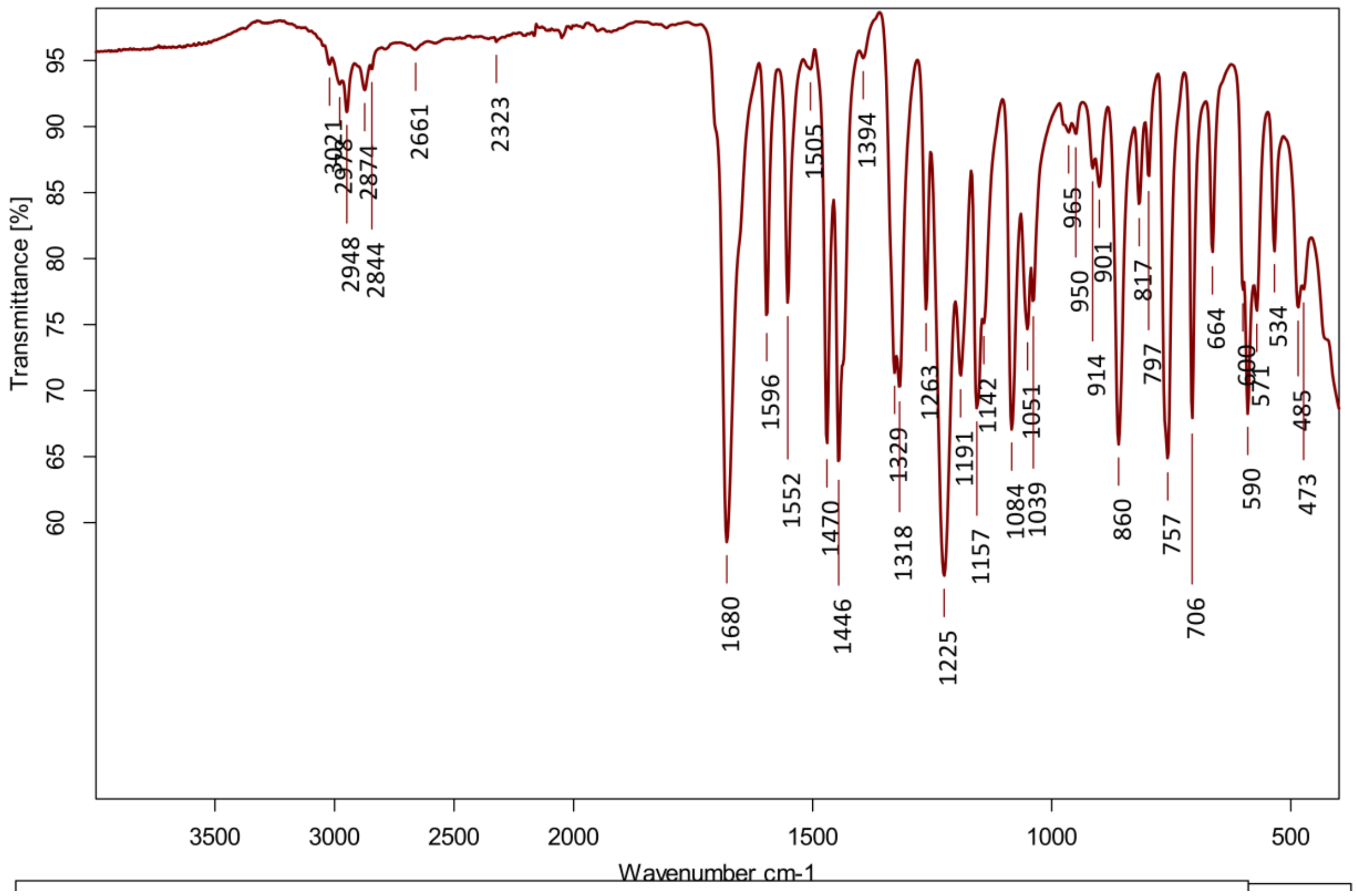


**Figure S33.** FTIR-ATR spectrum of **23**.

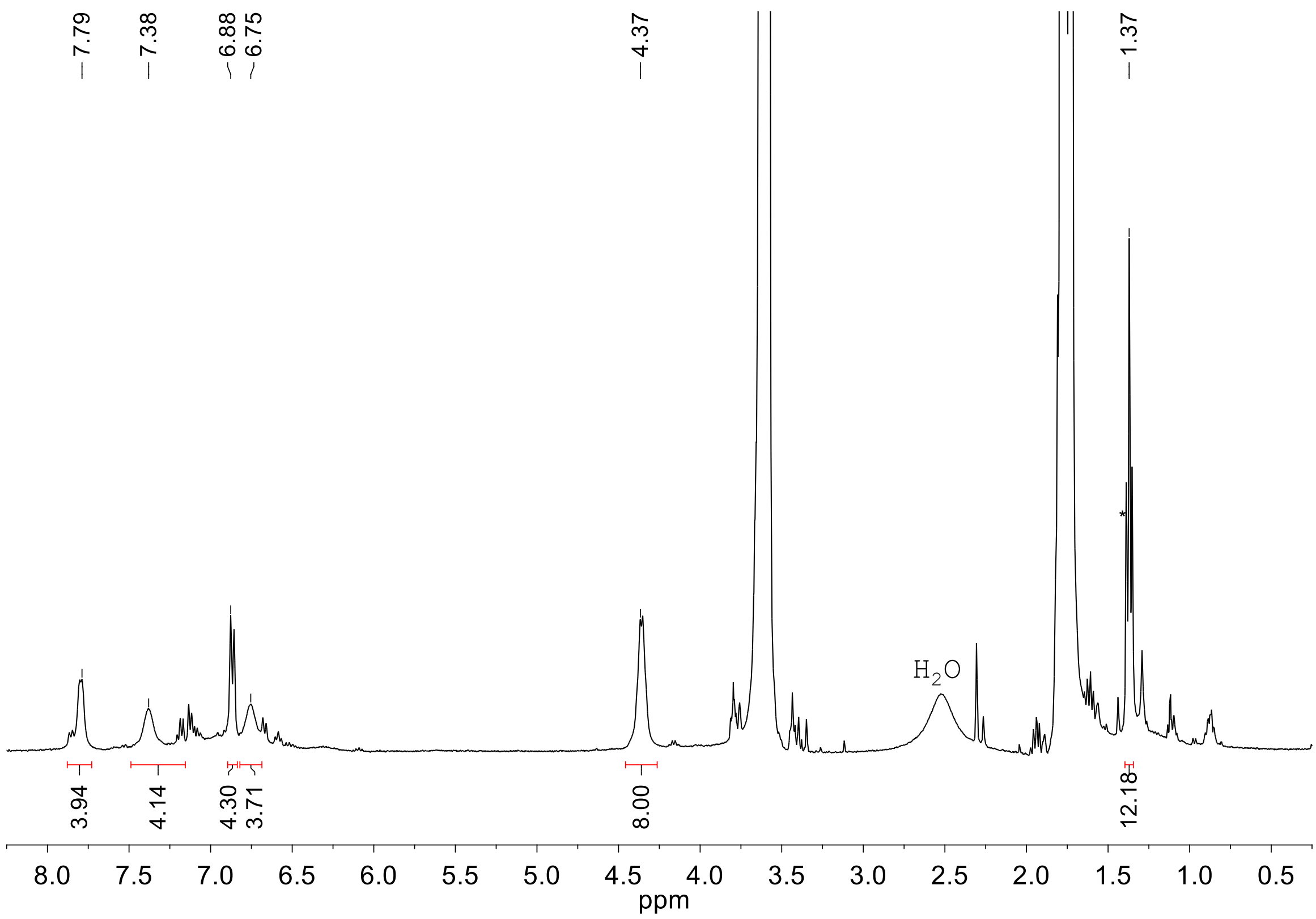


**Figure S34.** $^1$H NMR spectrum of **24** in THF-$d_8$.

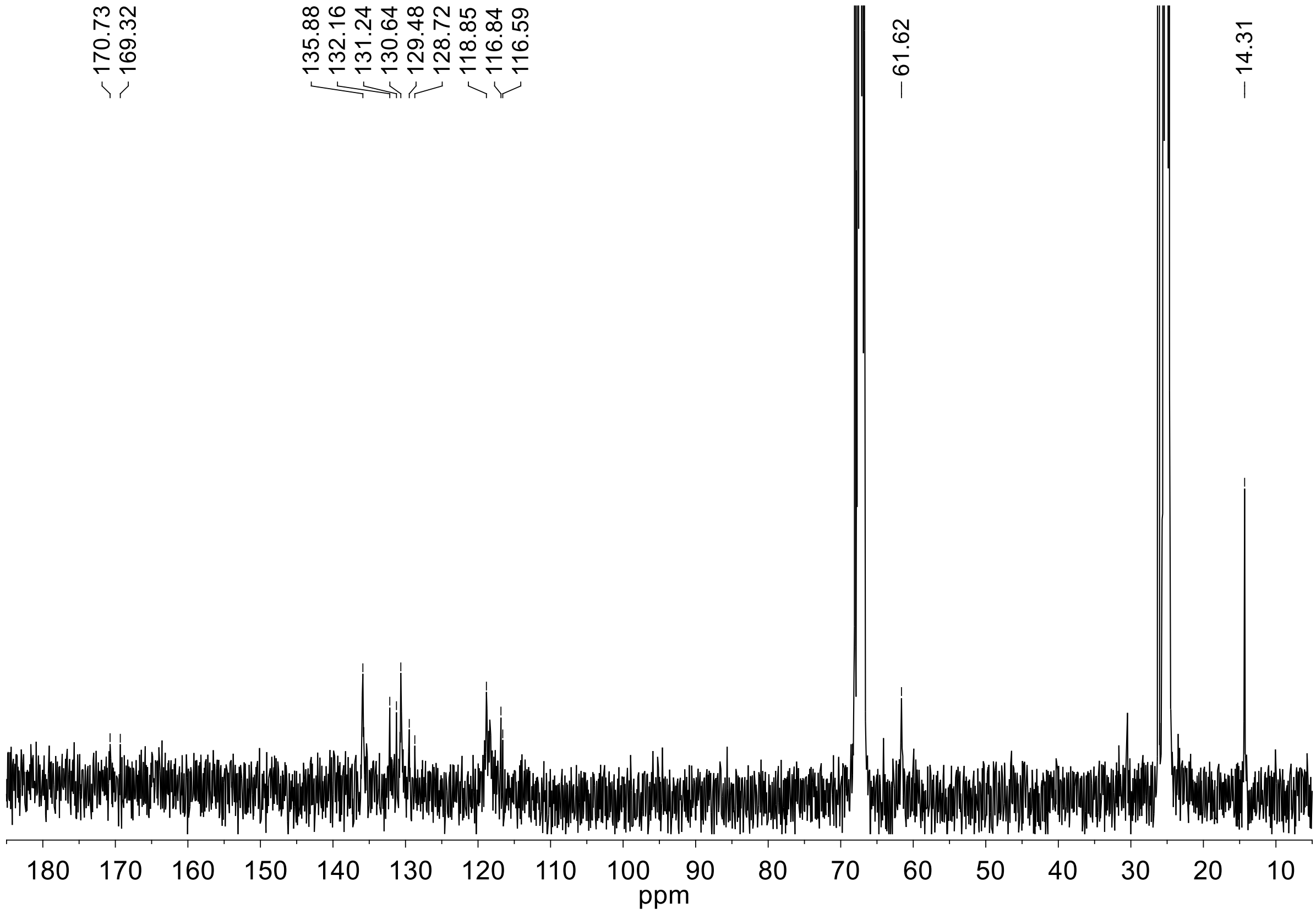


**Figure S35.** $^{13}C$ NMR spectrum of **24** in THF-$d_8$.

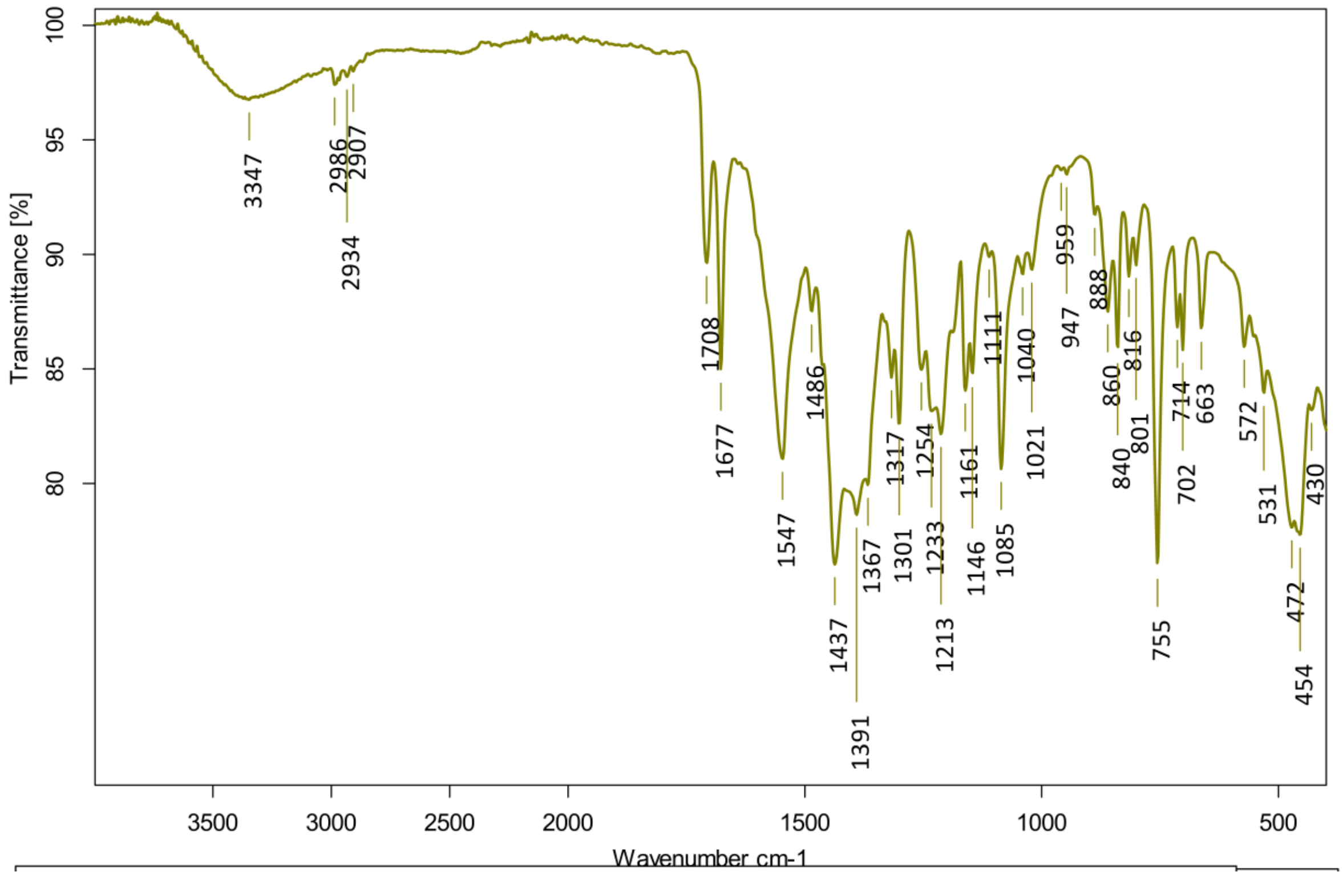


**Figure S36.** FTIR-ATR spectrum of **24**.

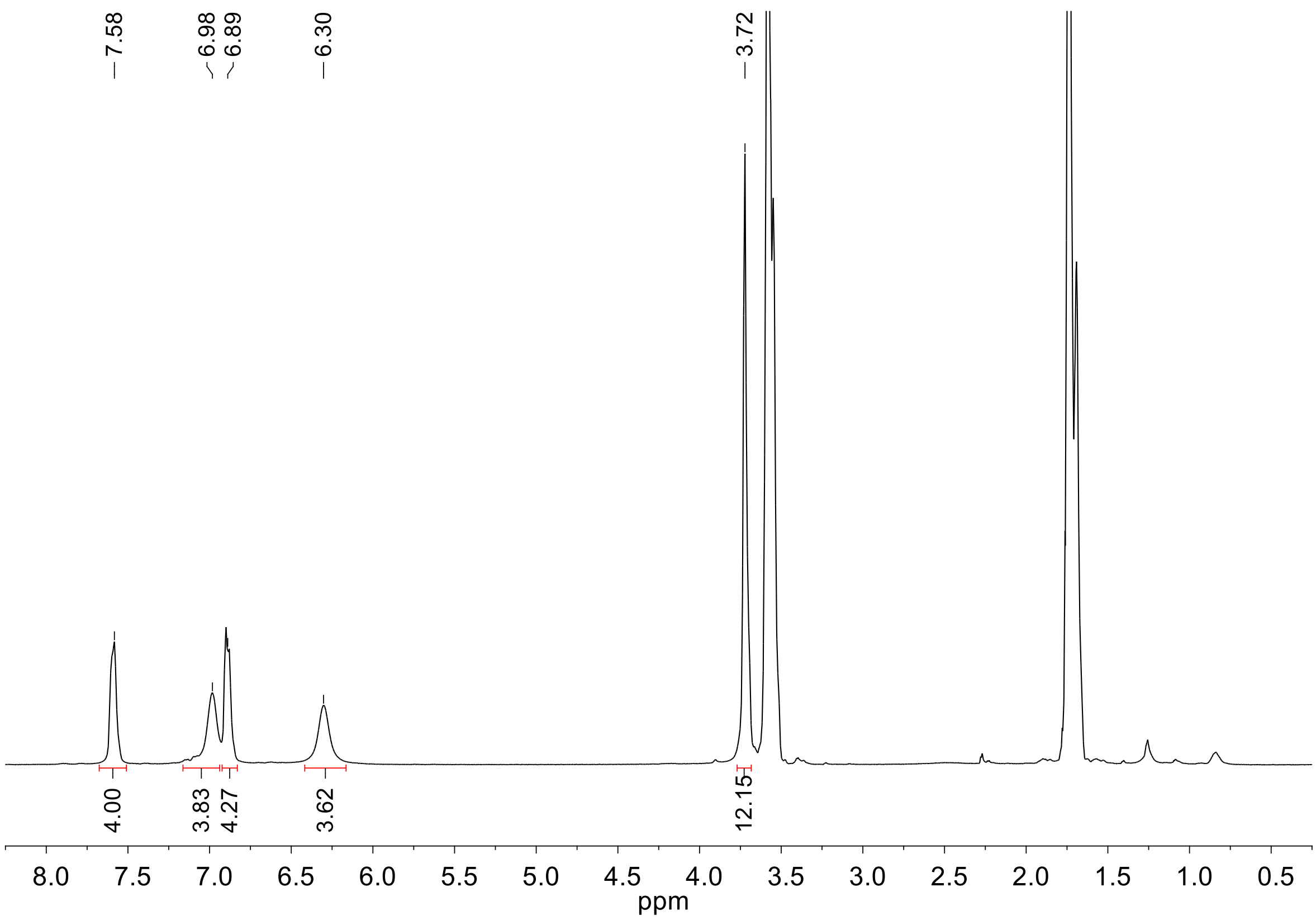


**Figure S37.** $^{1}$H NMR spectrum of **25** in THF-$d_8$.

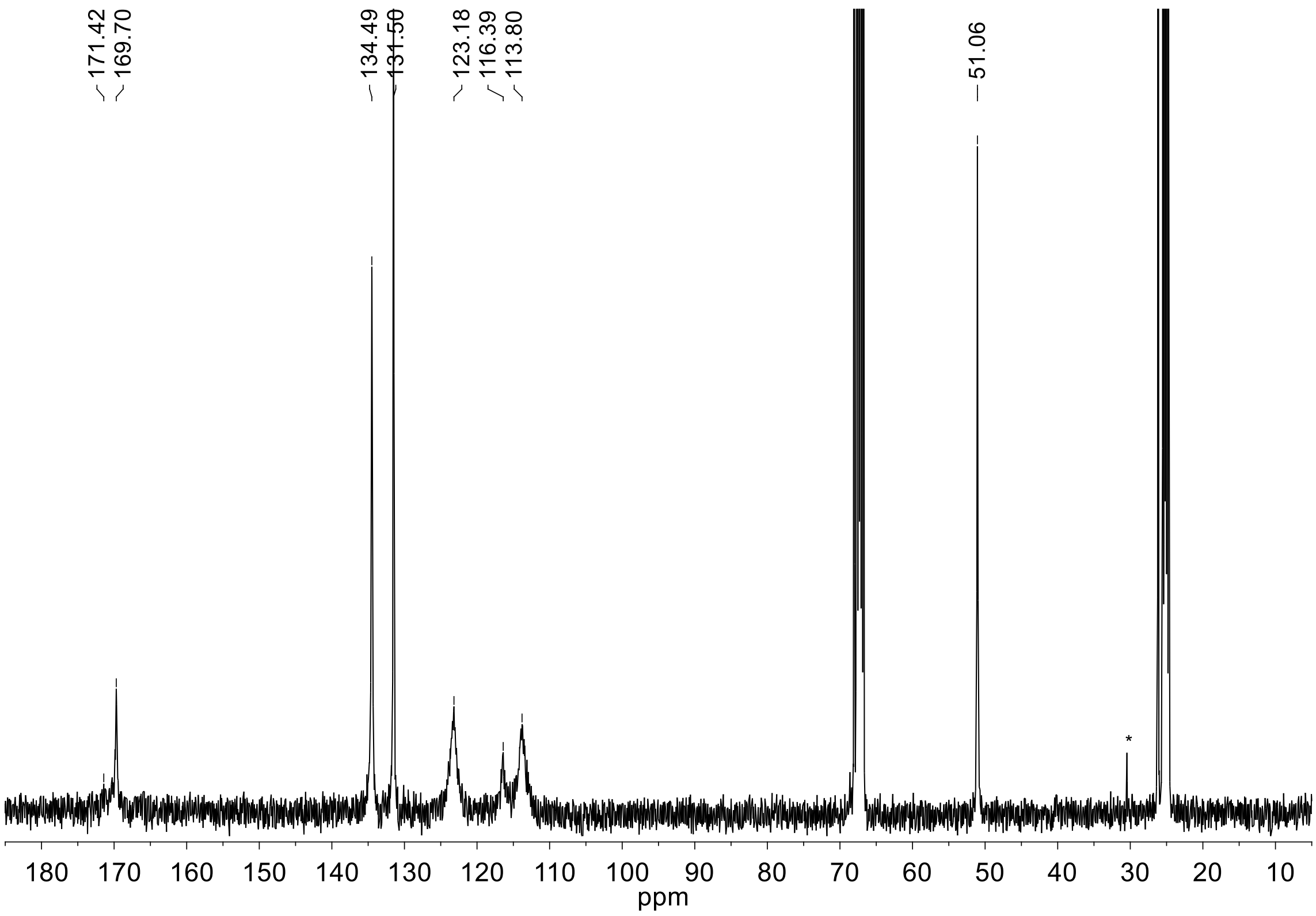


**Figure S38.** $^{13}$C NMR spectrum of **25** in THF-$d_8$.

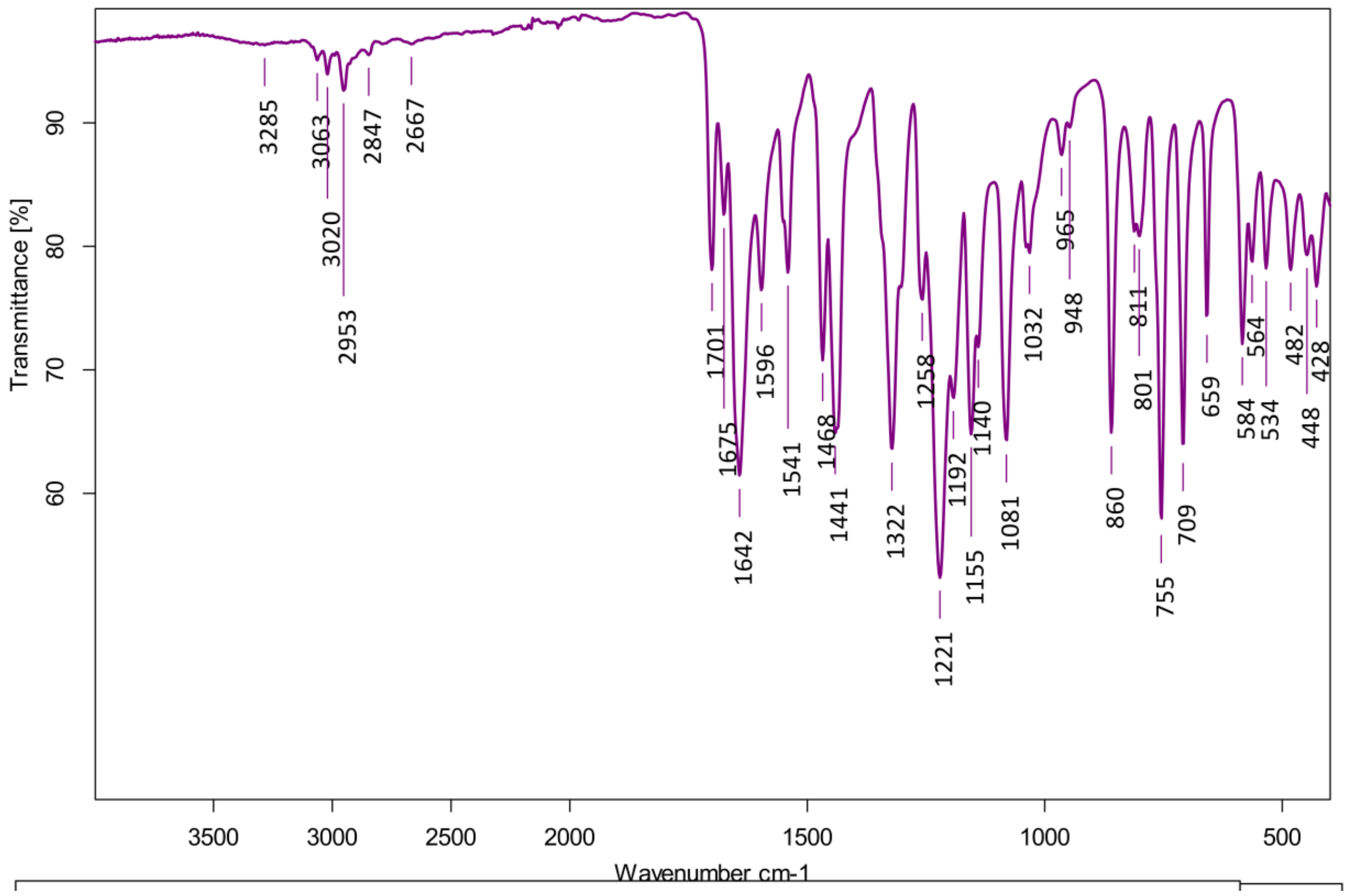


**Figure S39.** FTIR-ATR spectrum of **25**.

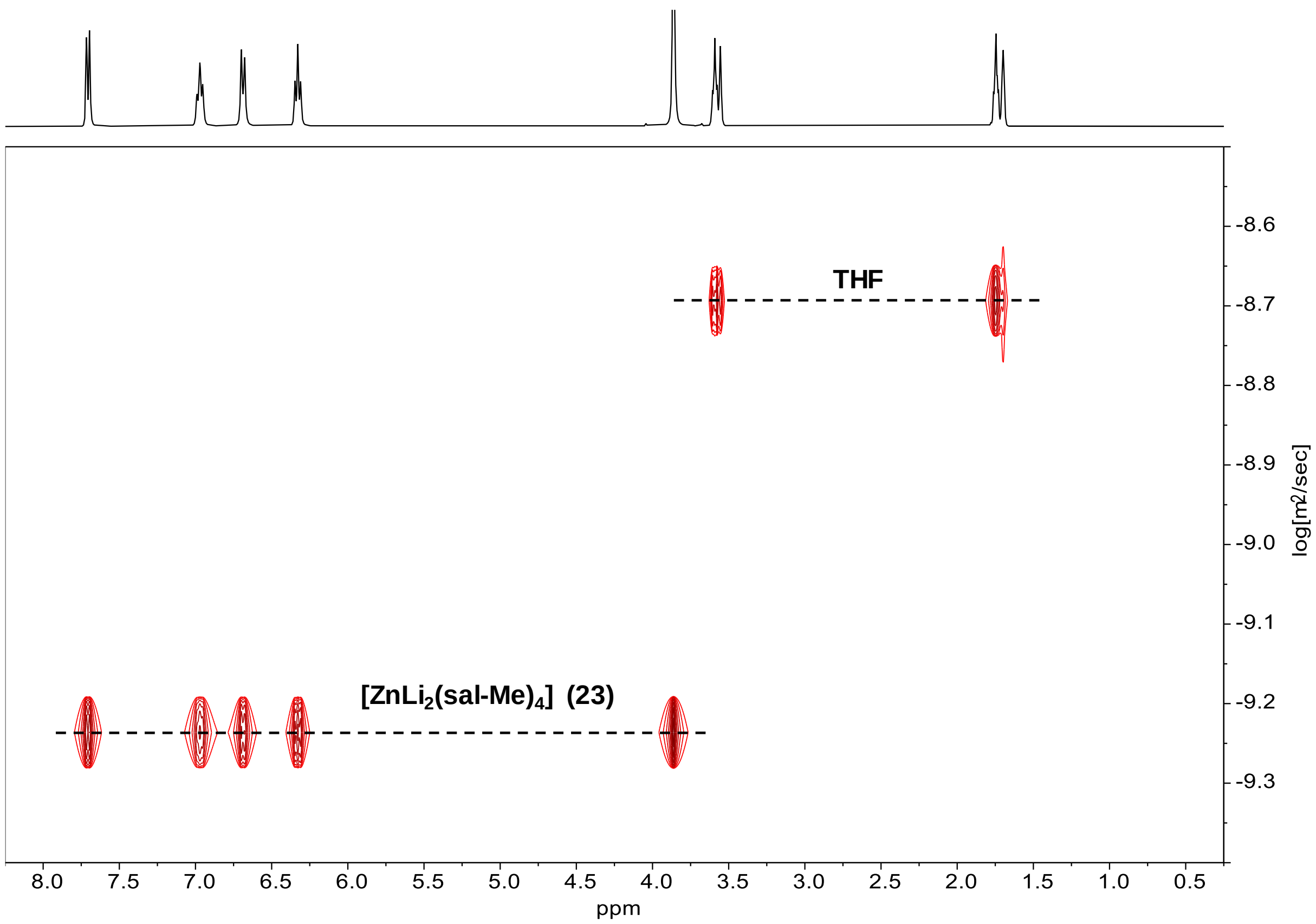


**Figure S40.** $^{1}$H-DOSY NMR spectrum of **23** in THF-d$_8$.

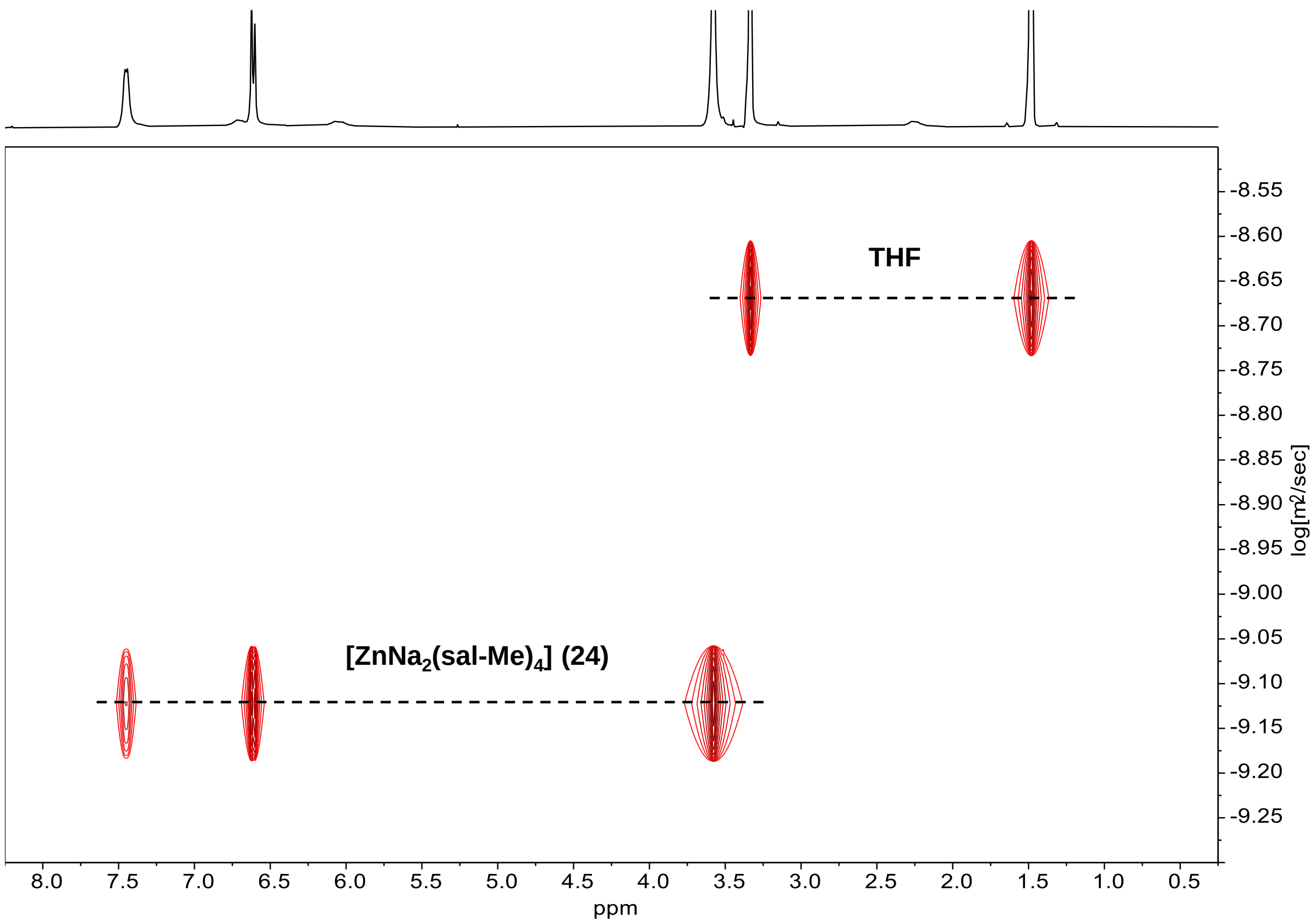


**Figure S41.** $^{1}$H-DOSY NMR spectrum of **24** in THF-$d_8$.

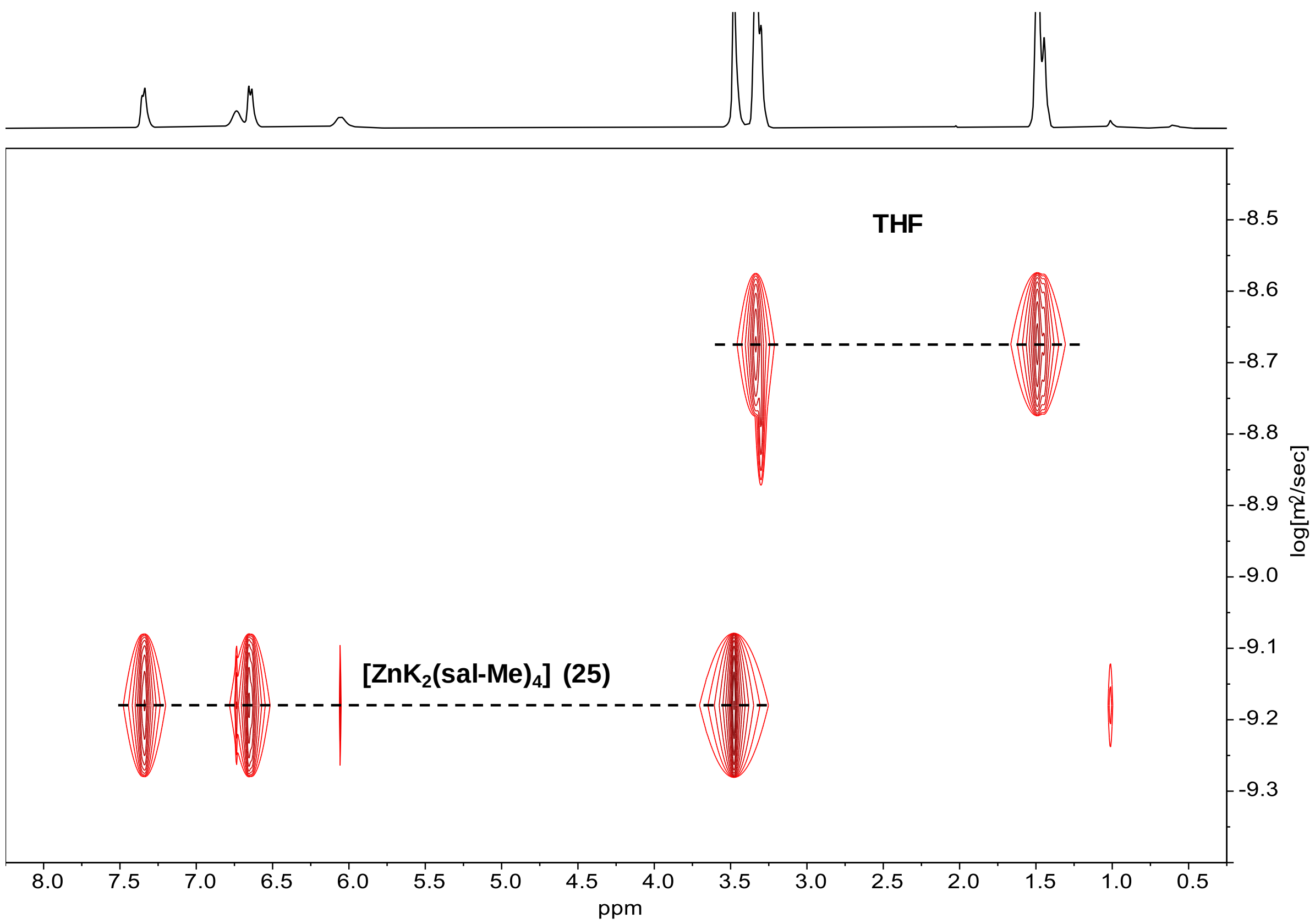


**Figure S42.** $^{1}$H-DOSY NMR spectrum of **25** in THF-$d_8$.

**Section 1**. FTIR-ATR analysis of PET/PEICT and their butanolysis products.

The FTIR spectrum of partially crystalline PET (Figure S43a) reveals a sharp band at 1714 $cm^{-1}$ corresponding to C=O stretching of the terephthalate unit and an asymmetric C–O–C stretching band at 1240 $cm^{-1}$. C=C stretching vibrations appear at 1614, 1578, and 1505 $cm^{-1}$, while the band at 1408 $cm^{-1}$ is attributed to aromatic skeletal stretching. Aromatic in-plane and out-of-plane C–H vibrations are observed at 1017 and 872 $cm^{-1}$, and the 1,4-para-substituted benzene ring out-of-plane bending mode appears at 723 $cm^{-1}$. C–H stretching bands at 2964, 2923, and 2851 $cm^{-1}$, $CH_2$ bending and wagging modes at 1455, 1372, and 1338 $cm^{-1}$, and $CH_2$ rocking at 845 $cm^{-1}$ are characteristic of the ethylene glycol segment. Bands at 1118, 1092, and 969 $cm^{-1}$ correspond to C–O (alkyl/aryl) stretching vibrations, while ring deformations, C–C torsions, and skeletal modes appear in the 632-435 $cm^{-1}$ region.

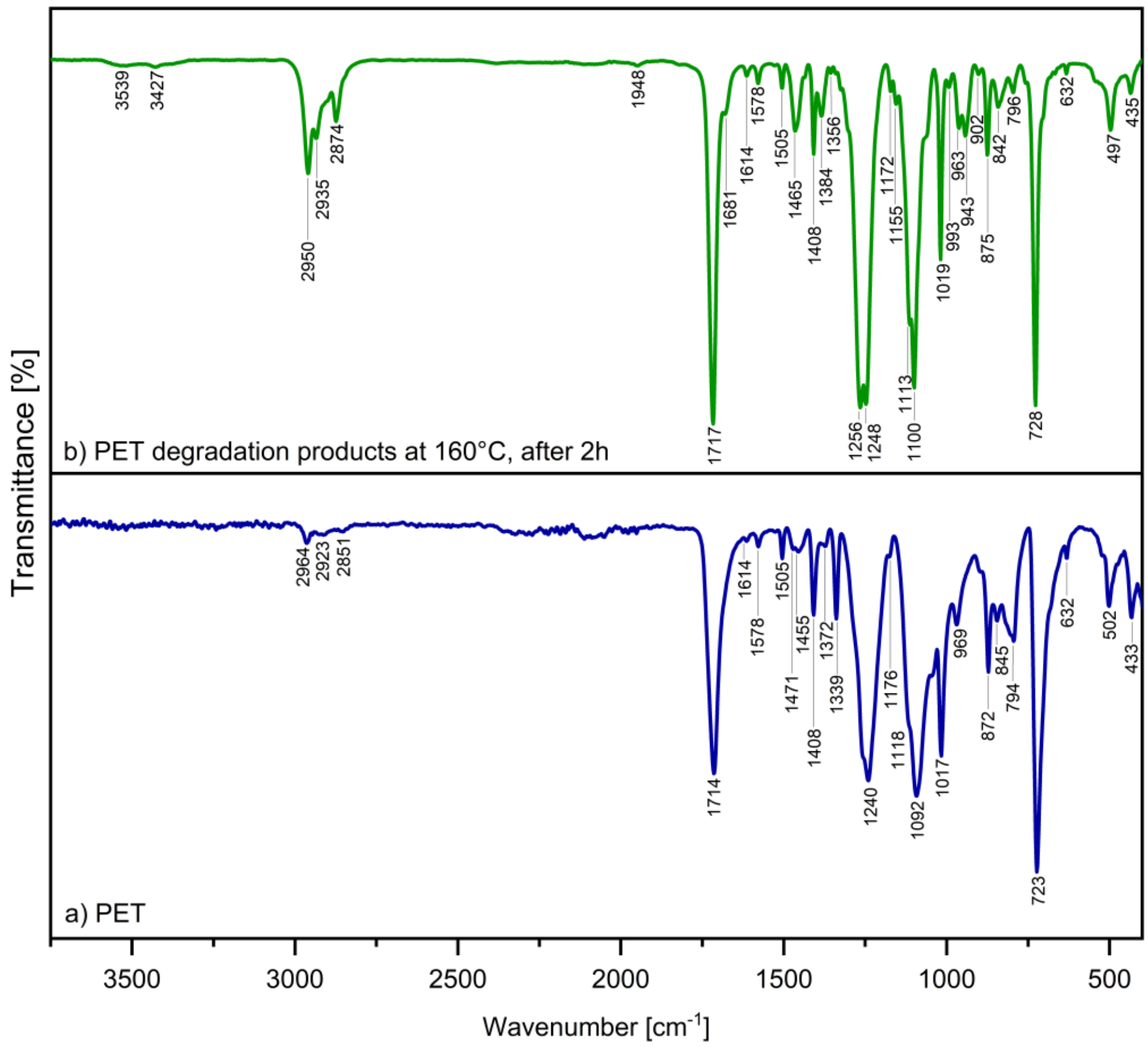


**Figure S43**. Comparison of FTIR-ATR spectra of virgin PET (a) and the liquid organic product obtained from PET alcoholysis using catalyst **1** after 2 h at 160 °C (b).

The FTIR spectrum of the reaction mixture obtained from PET butanolysis (Figure 43b) shows a broad O–H stretching band at 3600–3200 $cm^{-1}$, indicating release of ethylene

glycol. Enhanced alkyl C–H stretching bands at 2950, 2935, and 2874 cm$^{-1}$ relative to PET confirm formation of butyl ester groups. The formation of DBT is evidenced by a strong carbonyl stretching band at 1717 cm$^{-1}$, aromatic C=C stretching bands in the 1600–1500 cm$^{-1}$ region, and a para-substituted benzene out-of-plane vibration at 728 cm$^{-1}$ (Figure S44). Symmetric $\delta_s(CH_3)$ bending of the butyl chain is observed at 1384 cm$^{-1}$, while $CH_2$ rocking modes of –O–$CH_2$– and terminal –$CH_2$–$CH_3$ groups appear at 963 and 943 cm$^{-1}$. $CH_2$ scissoring vibrations of ester and diol groups occur at 1465 cm$^{-1}$. Strong absorptions in the 1256–1100 cm$^{-1}$ range correspond to asymmetric C–O–C and C–O stretching modes of esters, overlapping with alcohol C–O vibrations.

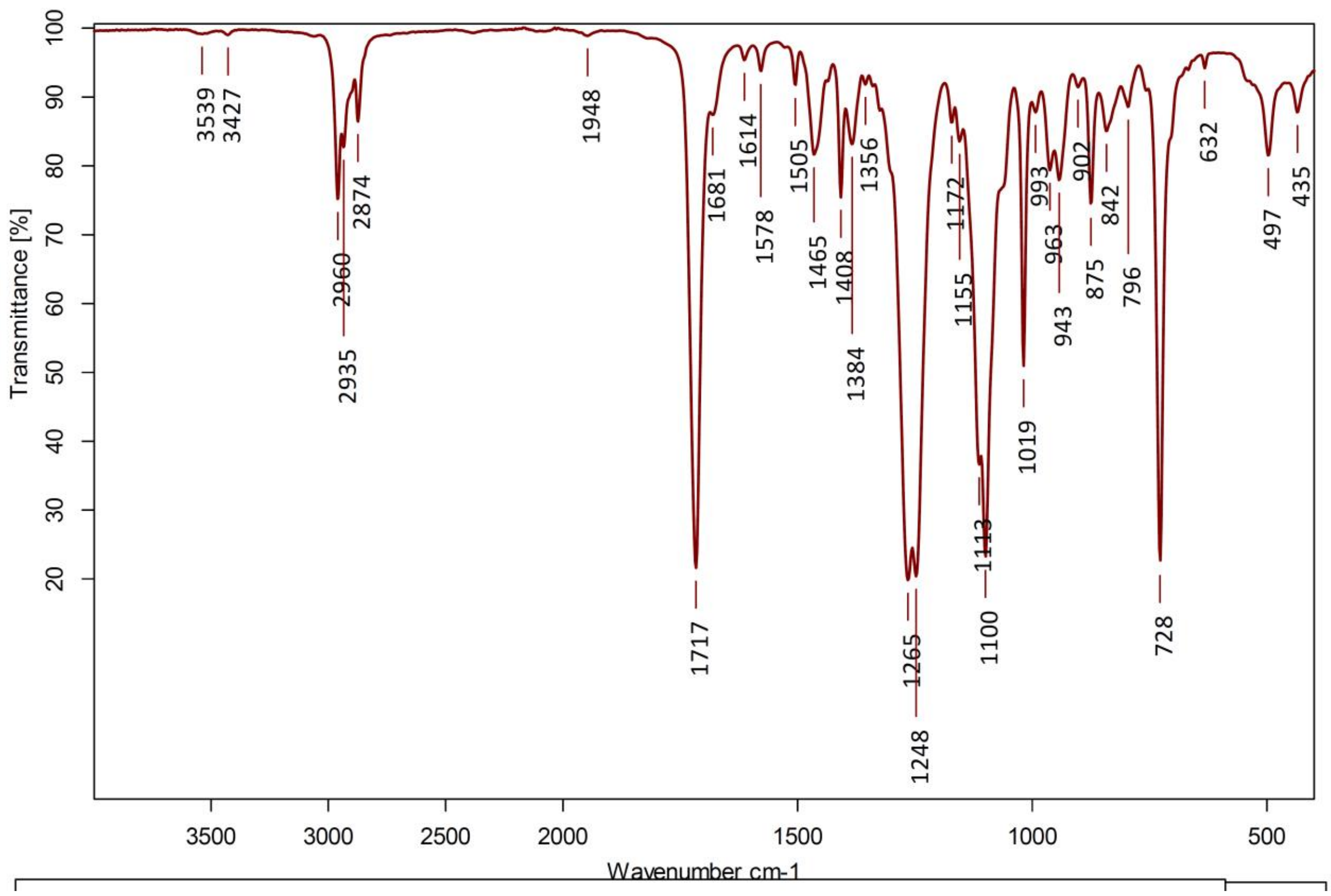


**Figure S44.** FTIR-ATR spectrum of **DBT**.

The FTIR spectrum of PEICT (Figure 45a) exhibits characteristic terephthalate absorptions similar to those described above (C=O stretching at 1714 cm$^{-1}$; C–O–C stretching at 1243 cm$^{-1}$; C=C stretching at 1613, 1578, and 1504 cm$^{-1}$; aromatic skeletal stretching at 1408 cm$^{-1}$; aromatic C–H vibrations at 1017, 872 cm$^{-1}$, 725 cm$^{-1}$).

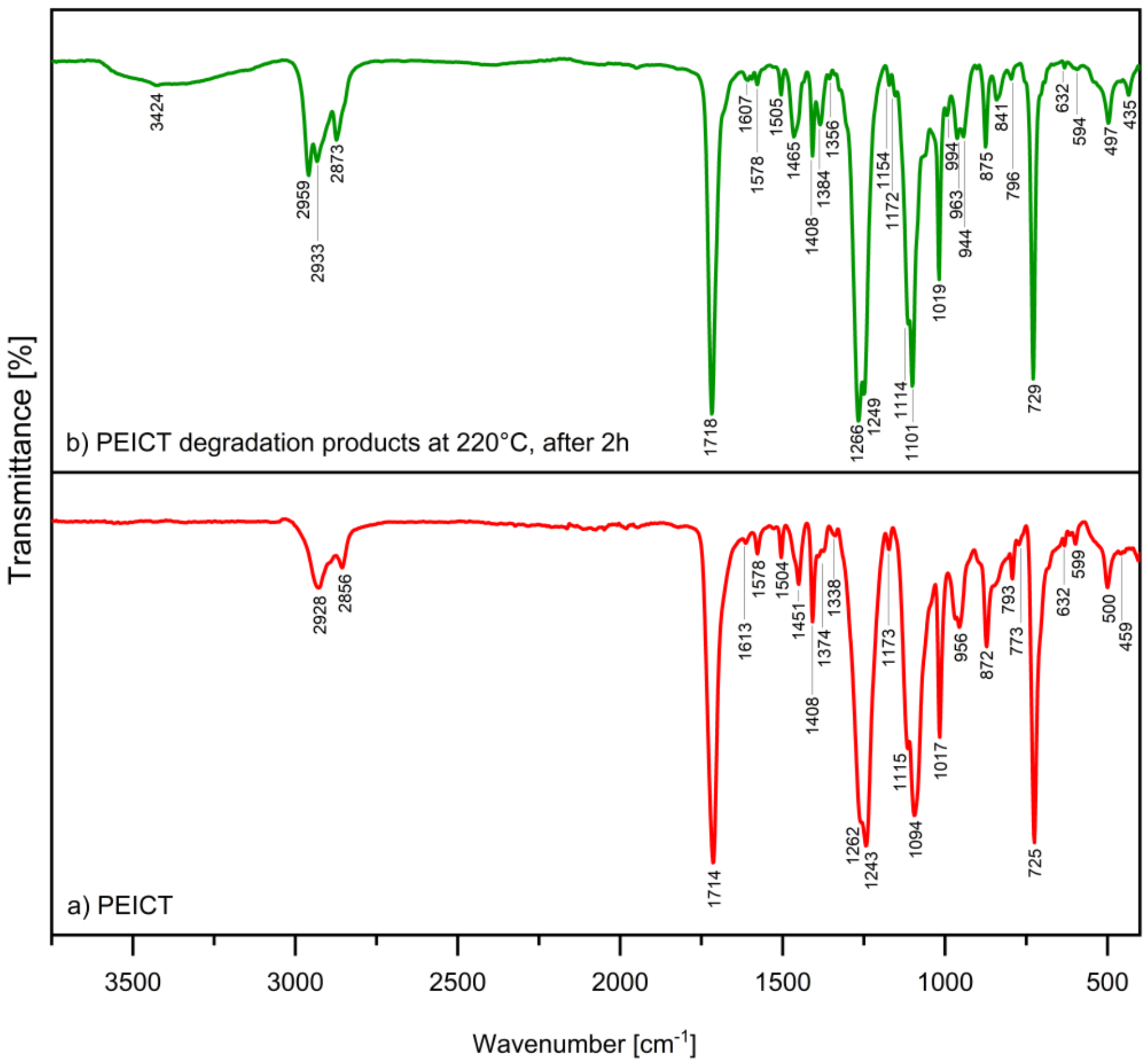


**Figure S45**. Comparison of FTIR-ATR spectra of virgin PEICT (a) and the liquid organic product obtained from PEICT alcoholysis using catalyst **1** after 2 h at 220 °C (b).

Broad aliphatic C–H stretching bands are observed at 2928 and 2856 cm$^{-1}$, and the band at 1451 cm$^{-1}$ is characteristic of aliphatic backbone vibrations ($CH_2$ scissoring of ethylene and cyclohexane units). Bands at 1115, 1094, and 956 cm$^{-1}$ correspond to C–O stretching, while ring deformations, C–C torsions, and skeletal modes of cyclic isosorbide and cyclohexane units appear in the 600-450 cm$^{-1}$ region.[1] The FTIR spectrum of the PEICT alcoholysis reaction mixture (Figure 45b) confirms formation of DBT along with ethylene glycol, 1,4-cyclohexanedimethanol, and isosorbide. DBT is identified by a strong carbonyl stretching band at 1718 cm$^{-1}$, aromatic C=C stretching bands in the 1600–1500 cm$^{-1}$ region, and a para-substituted benzene vibration at 729 cm$^{-1}$. Broad O–H stretching absorption at 3600–3200 cm$^{-1}$ indicates the presence of diols. Aliphatic C–H stretching bands appear at 2959, 2933, and 2873 cm$^{-1}$, while $CH_2$ scissoring and $CH_3$ deformation modes are observed at 1465 and 1384 cm$^{-1}$, reflecting the alkyl groups of ester and diols. Strong absorptions in the 1263–1101 cm$^{-1}$ range are assigned to asymmetric ester C–O–C and C–O stretching and overlapping alcohol C–O modes.

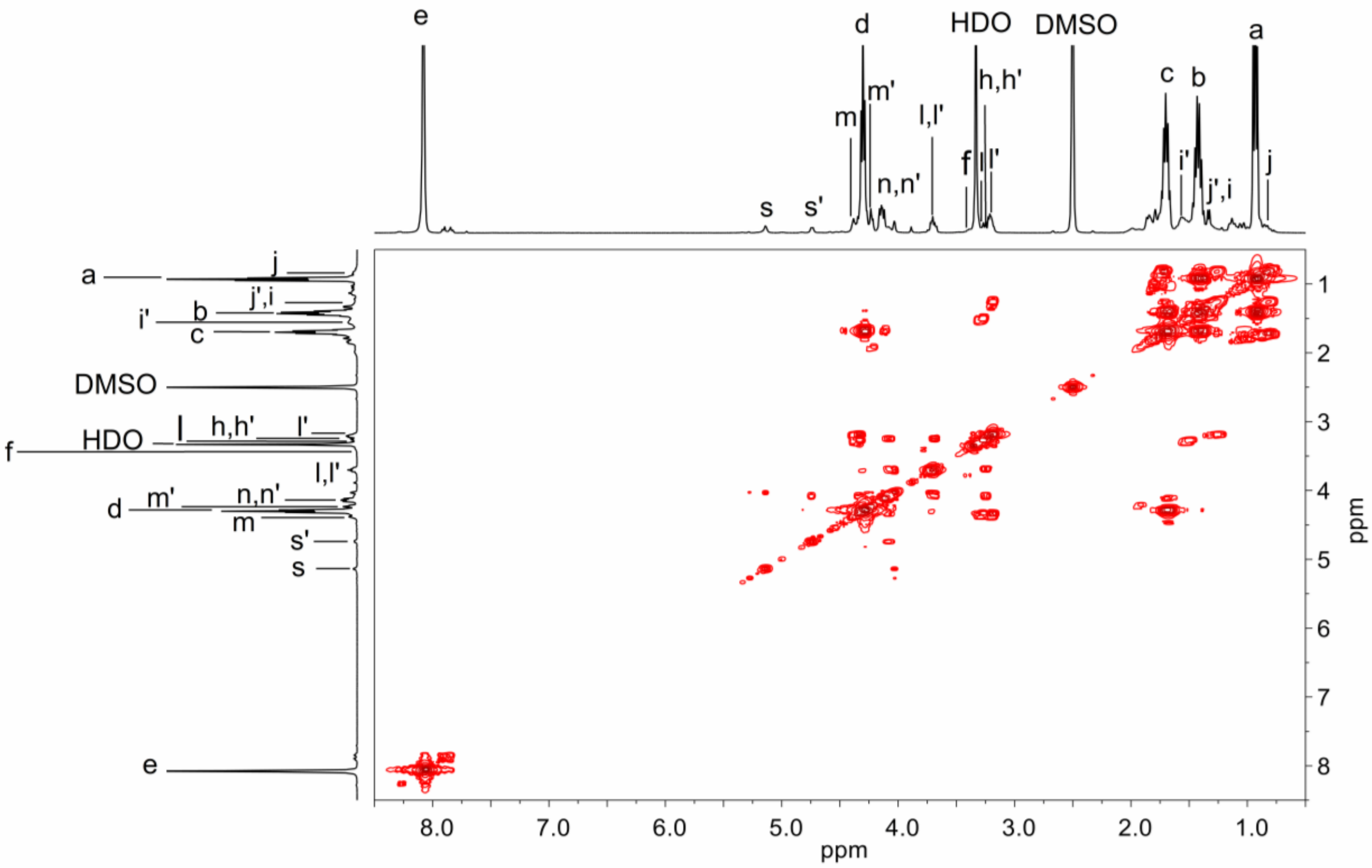


**Figure S46.** $^1H$-$^1H$ COSY NMR spectrum of PEICT butanolysis reaction mixture using **1** as a catalyst after 10 h at 240°C in DMSO-$d_6$.

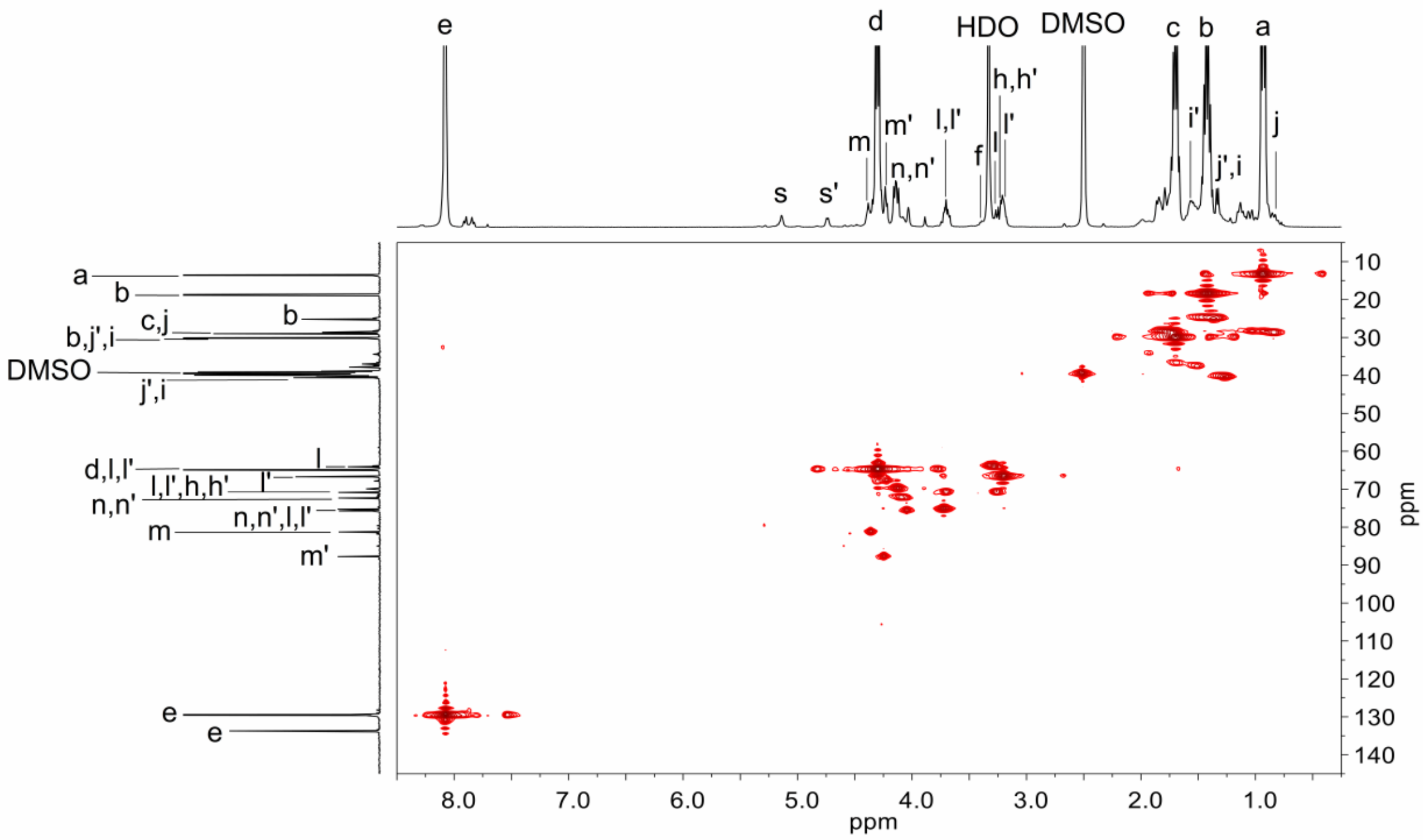


**Figure S47.** $^1H$-$^{13}C$ HSQC NMR spectrum of PEICT butanolysis reaction mixture using **1** as a catalyst after 10h at 240°C in DMSO-$d_6$.

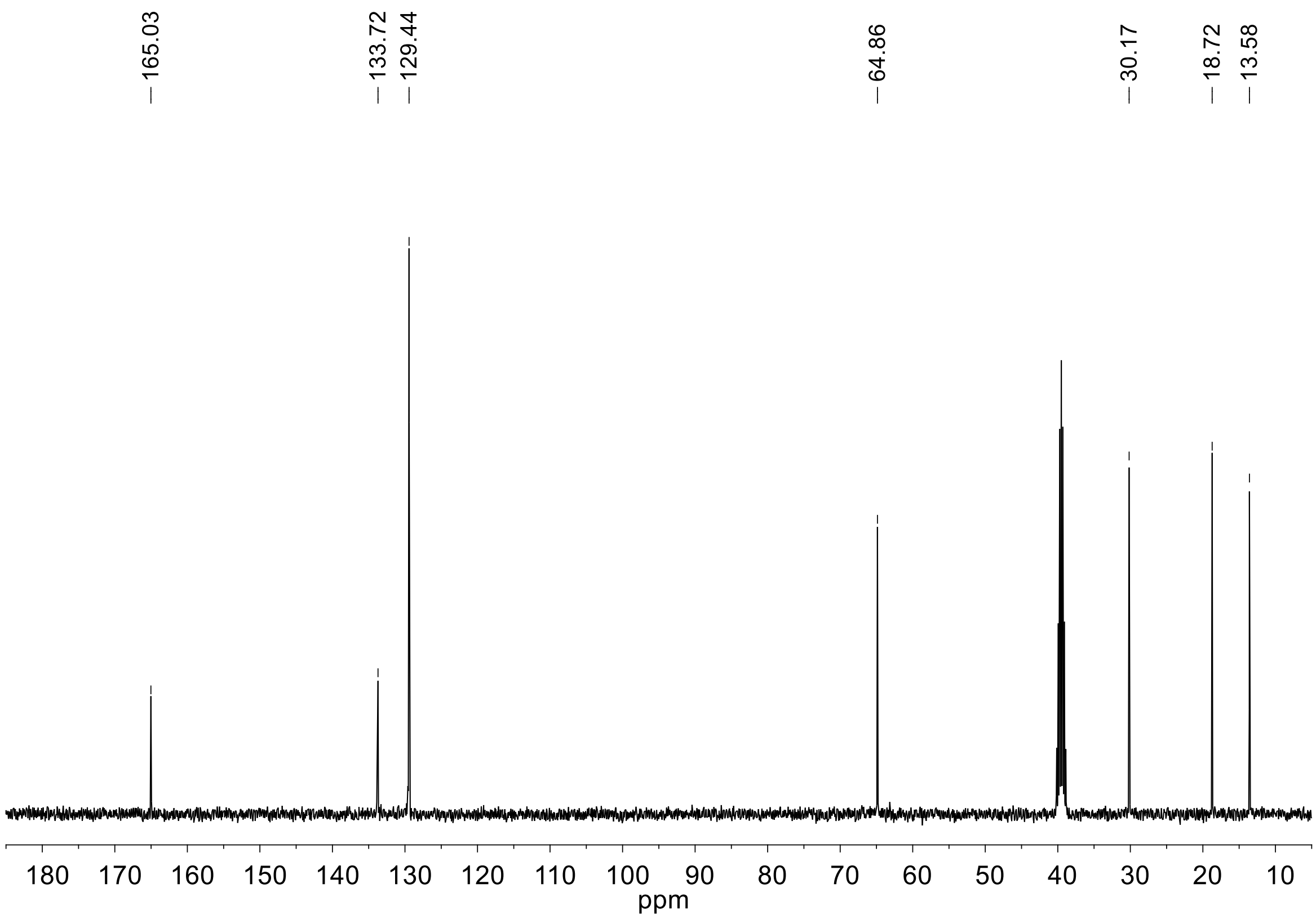


**Figure S48.** $^{13}$C NMR of spectrum of **DBT** in DMSO-$d_6$.

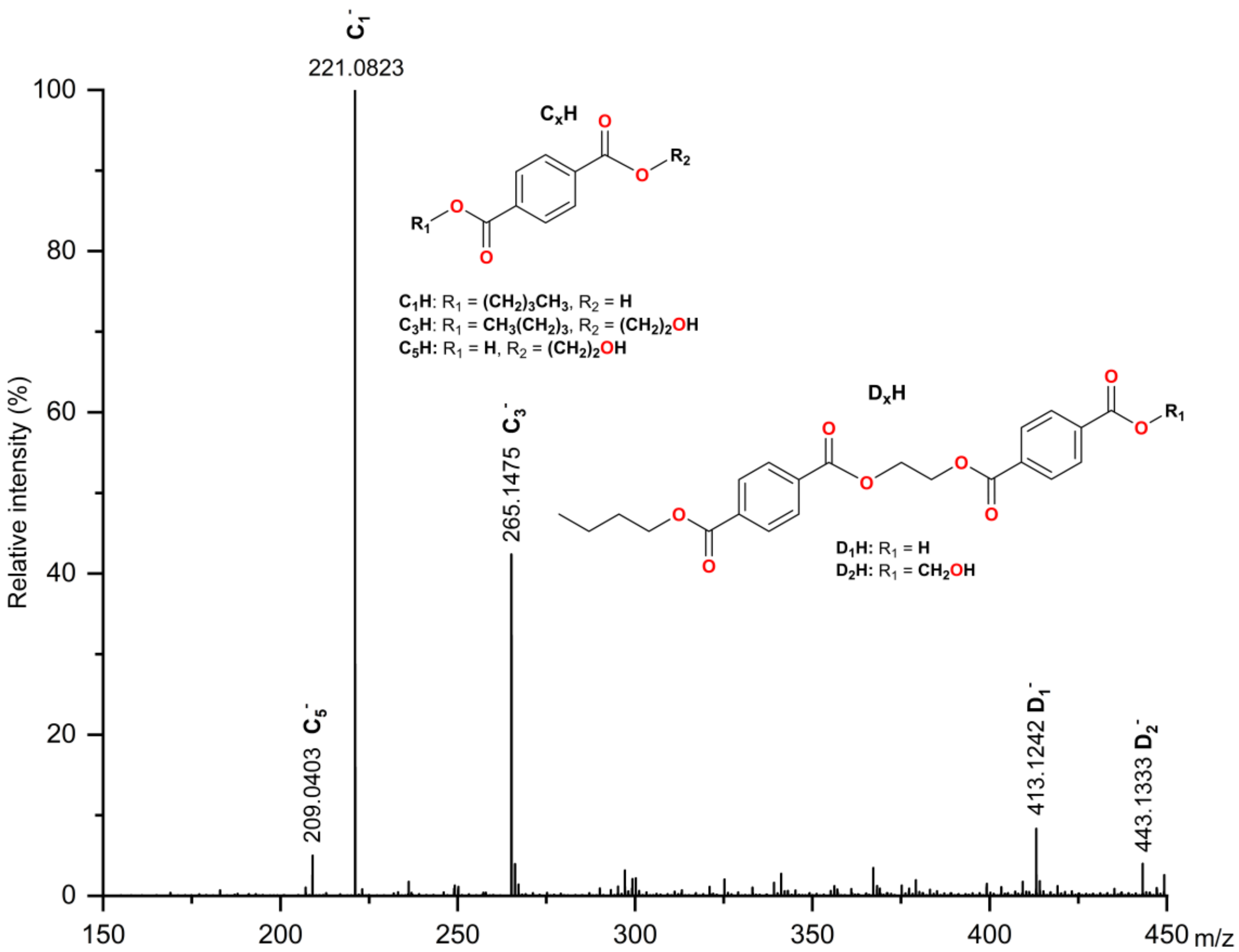


**Figure S49.** ESI-MS spectrum of organic products obtained from PET alcoholysis using **10** as a catalyst after 2h. An ESI-MS spectrum was recorded for fractions from 3.01 to 5.74 min.

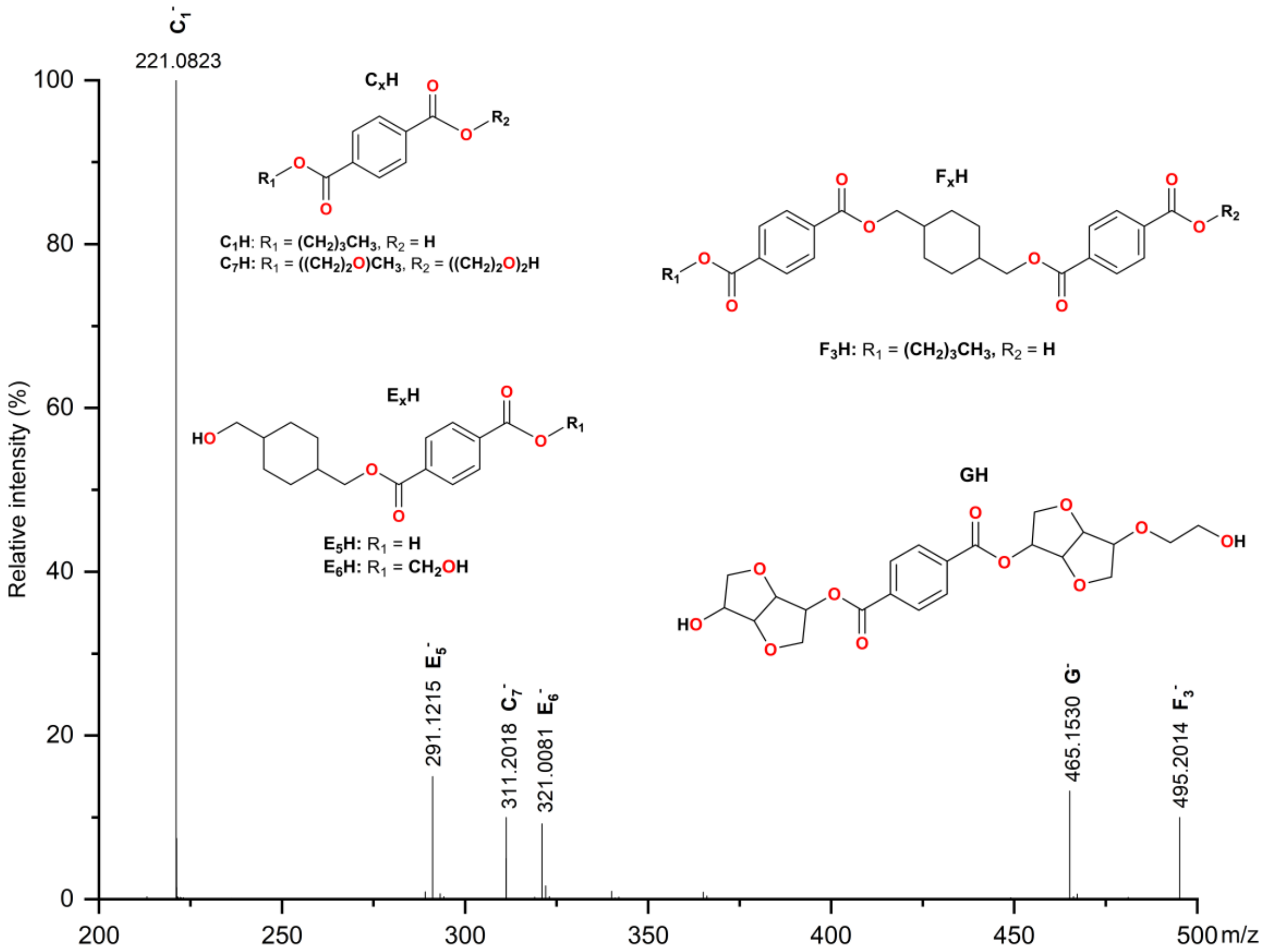


**Figure S50.** ESI-MS spectrum of organic products obtained from PEICT alcoholysis using **19** as a catalyst after 2h. An ESI-MS spectrum was recorded for fractions collected from 3.44 to 5.62 min.

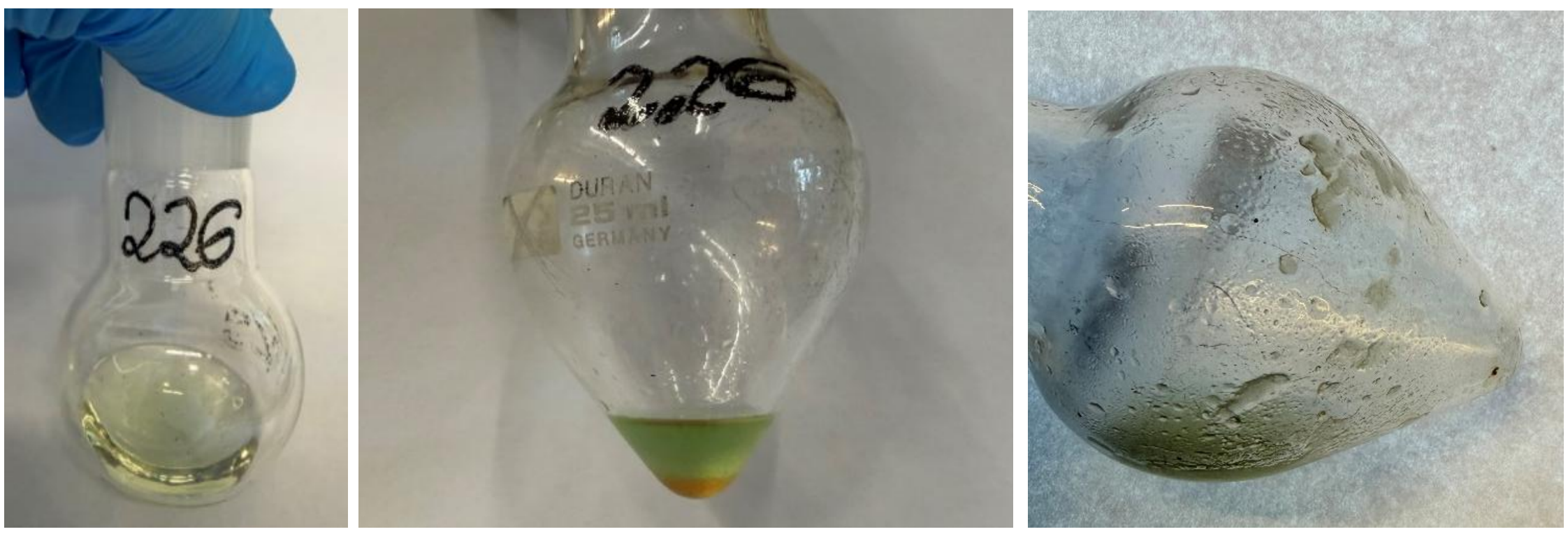


**Figure S51**. The liquid mixture after butanolysis of PET (left); the mixture of ethylene glycol and DBT after distillation of *n*-BuOH (middle); the obtained crystals of $[Li(BTA)]_n$ (right).

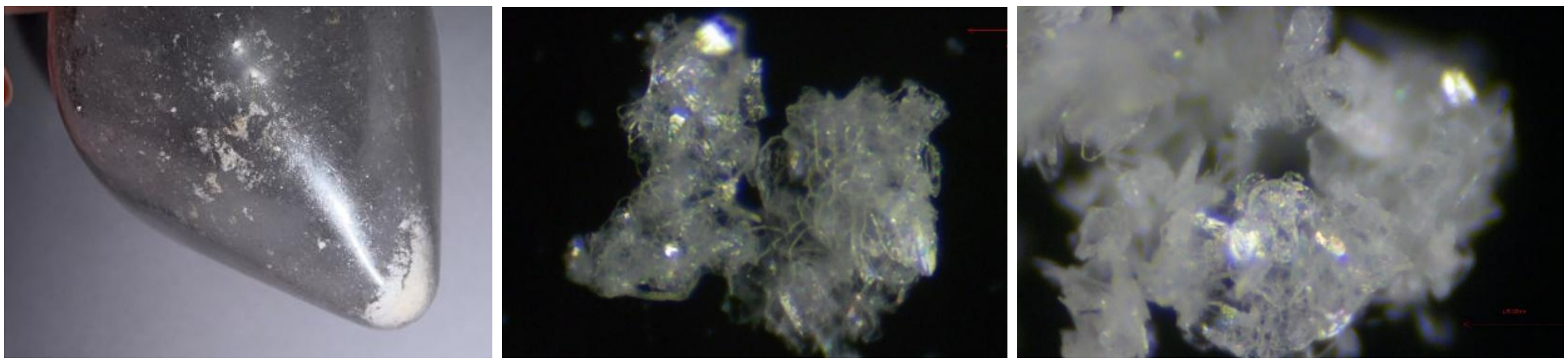

**Figure S52.** The crystalline material separated from the liquid mixture by hexane precipitation and filtration (left); crystals of $[Li(BTA)]_n$ under microscopic magnification (middle and right).

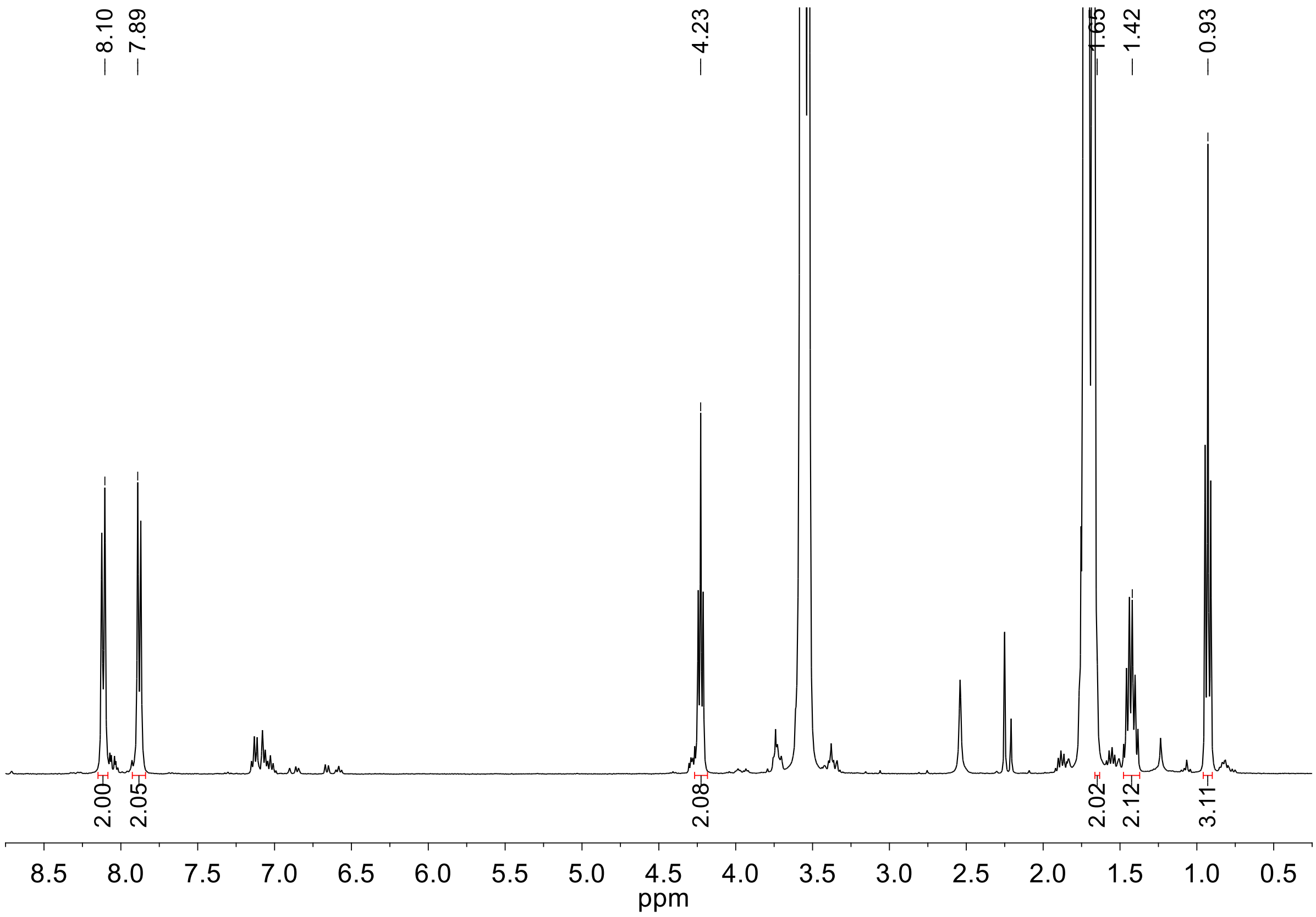


**Figure S53.** $^1$H NMR spectrum of **26** in THF-$d_8$.

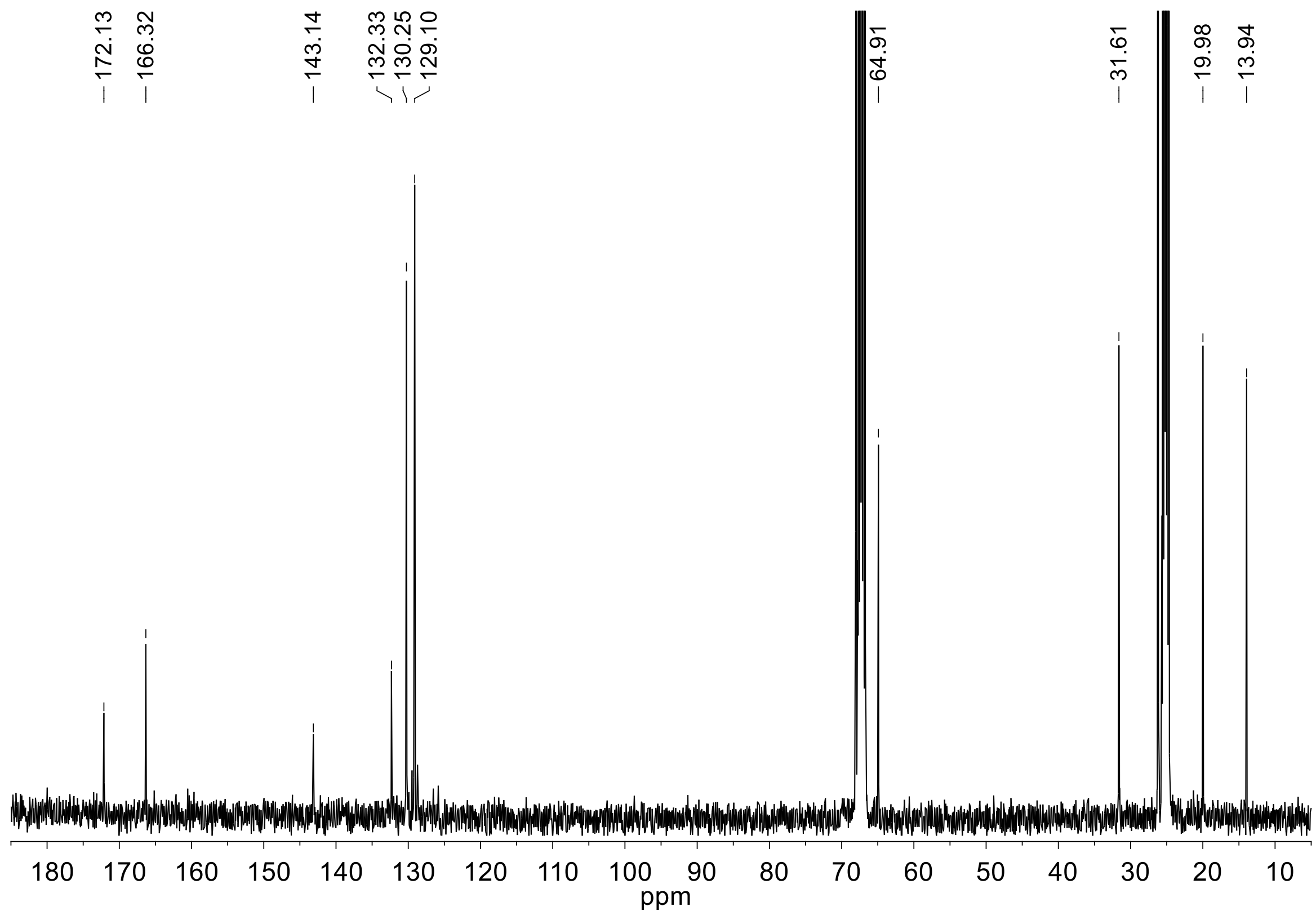


**Figure S54.** $^{13}C$ NMR spectrum of **26** in THF-$d_8$.

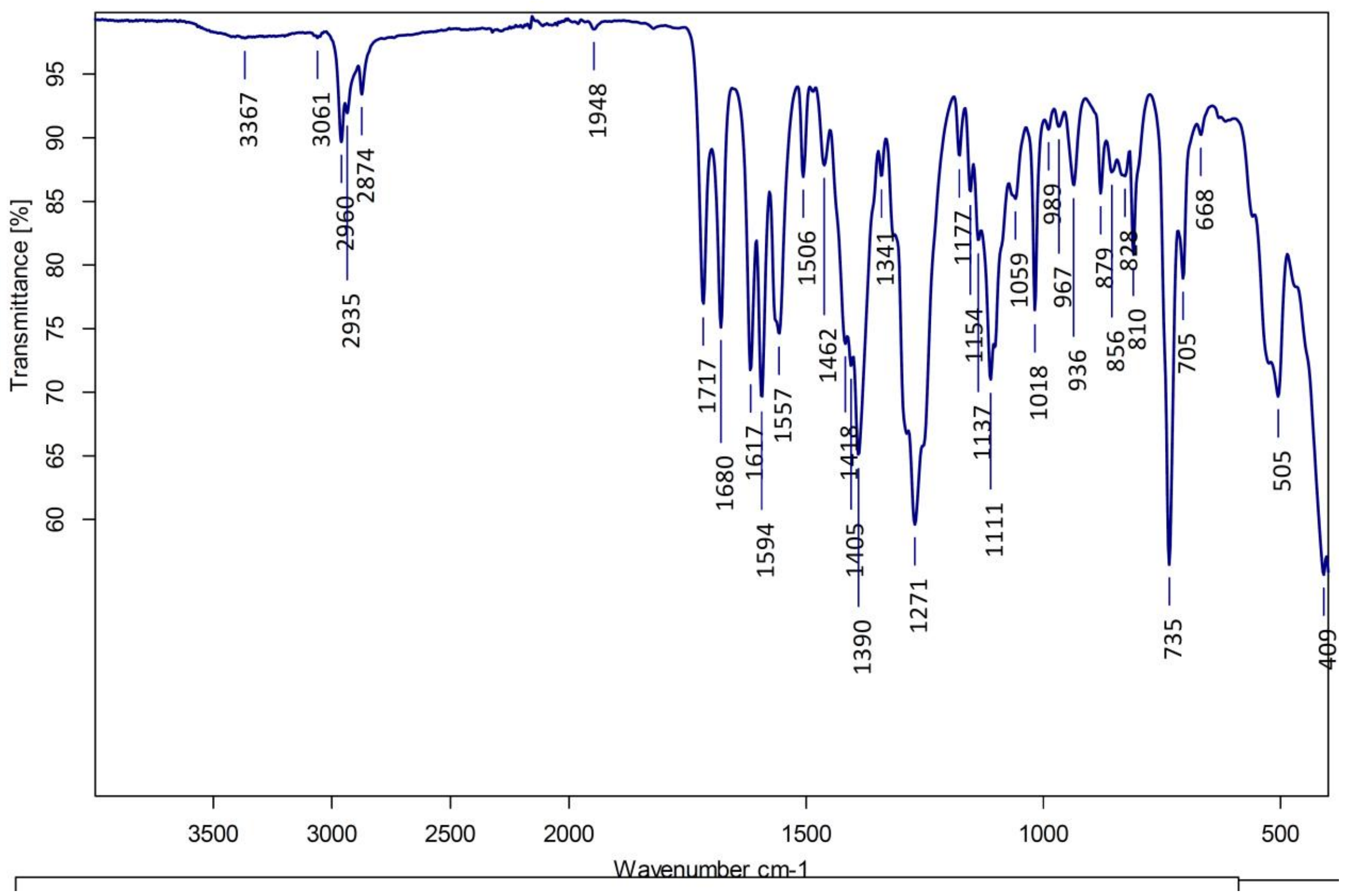


**Figure S55**. FTIR-ATR spectrum of **26**.

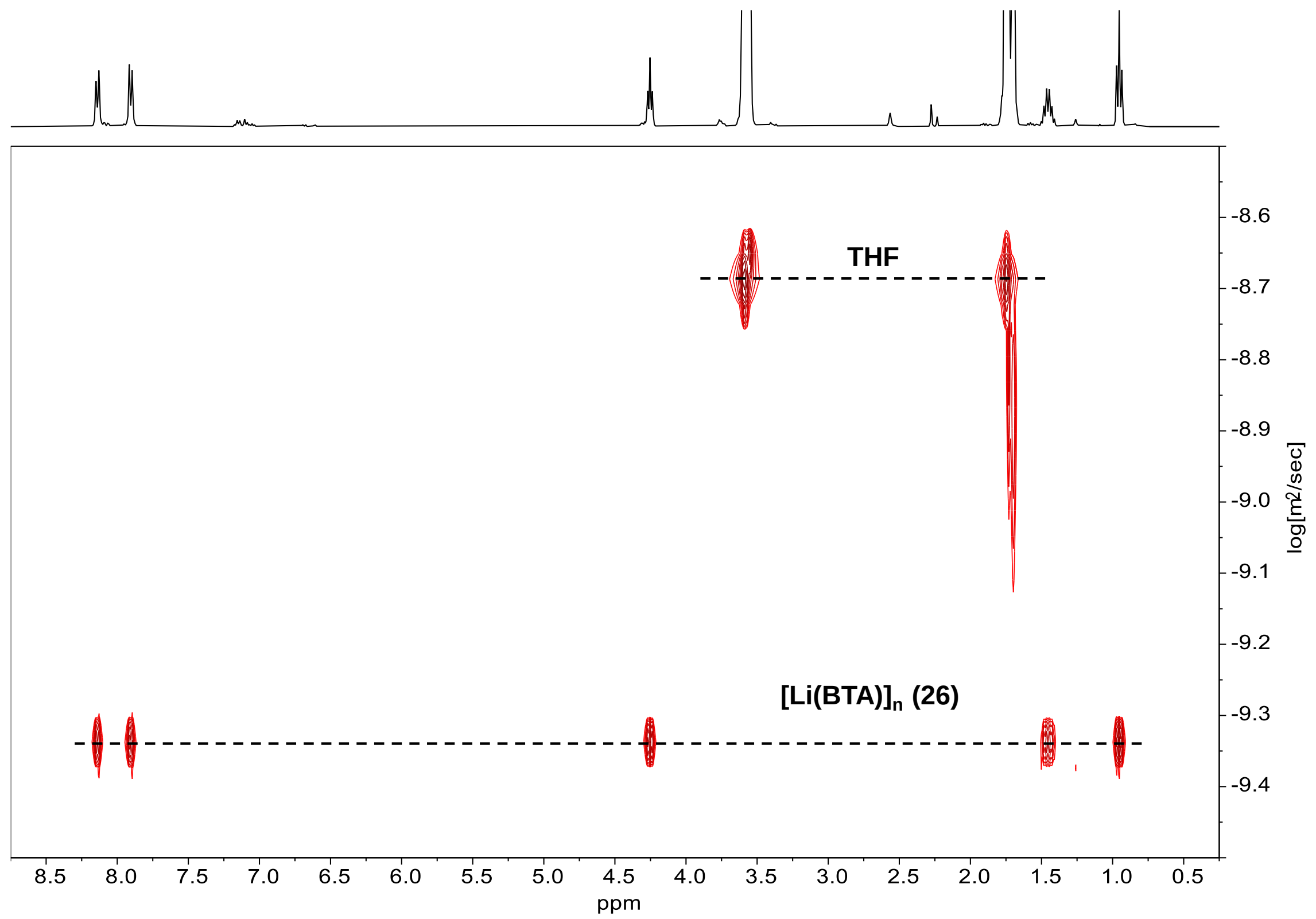


**Figure S56.** $^{1}$H-DOSY NMR spectrum of **26** in THF-$d_8$.

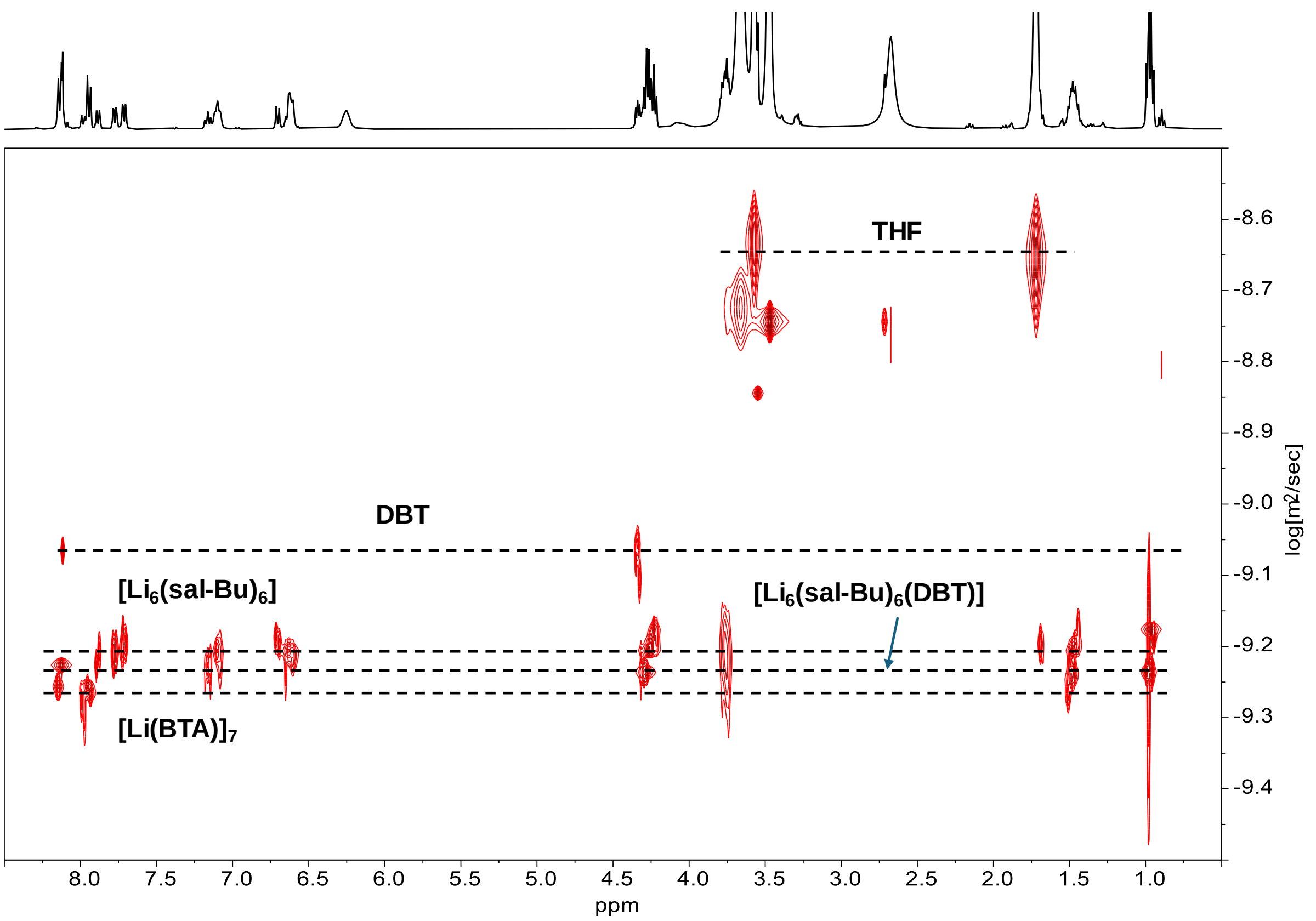


**Figure S57.** $^{1}$H DOSY NMR spectrum of the catalyst residue obtained from reactions using compound **1**.

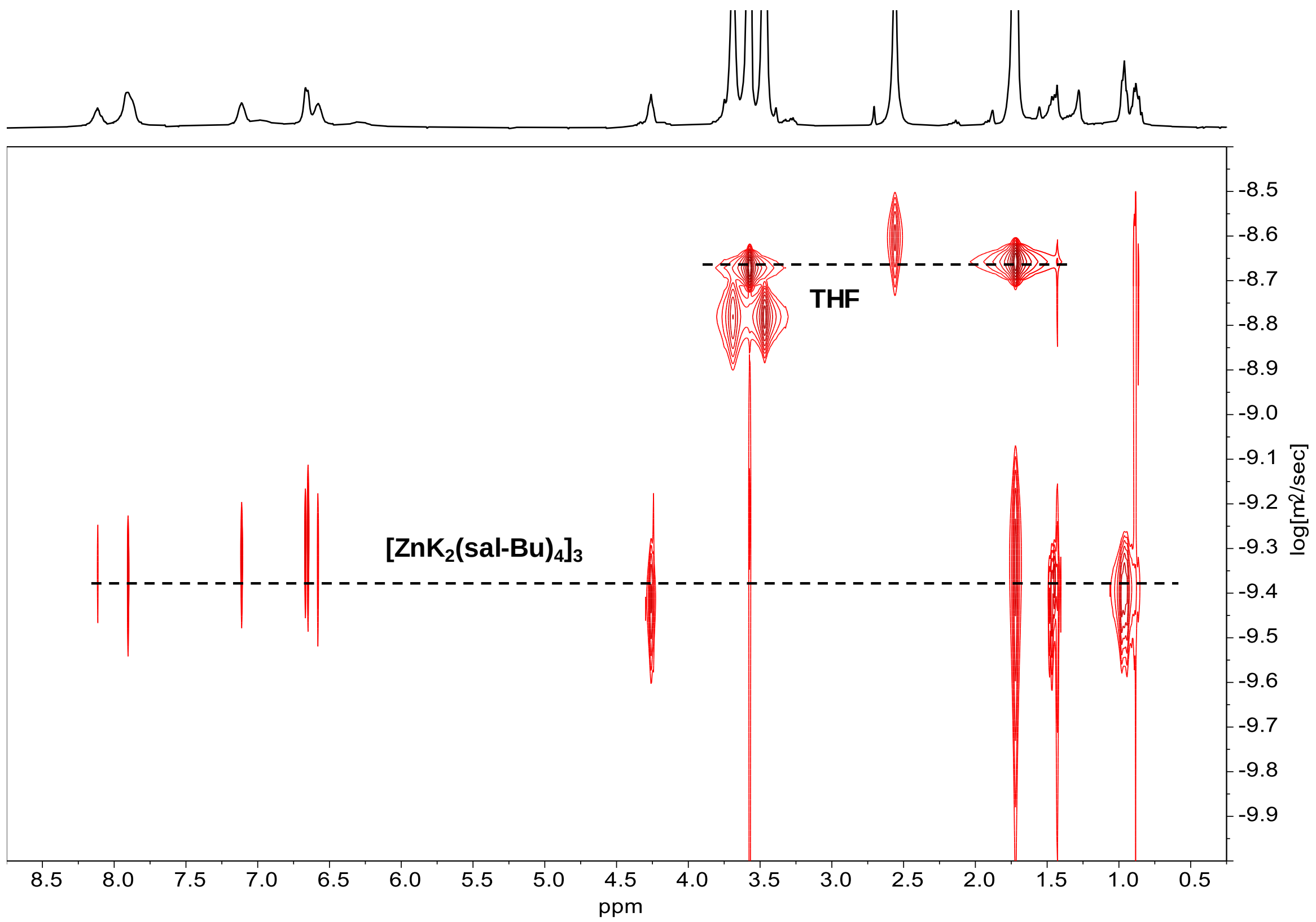


**Figure S58.** $^{1}$H DOSY NMR spectrum of the catalyst residue obtained from reactions using compound **25**.

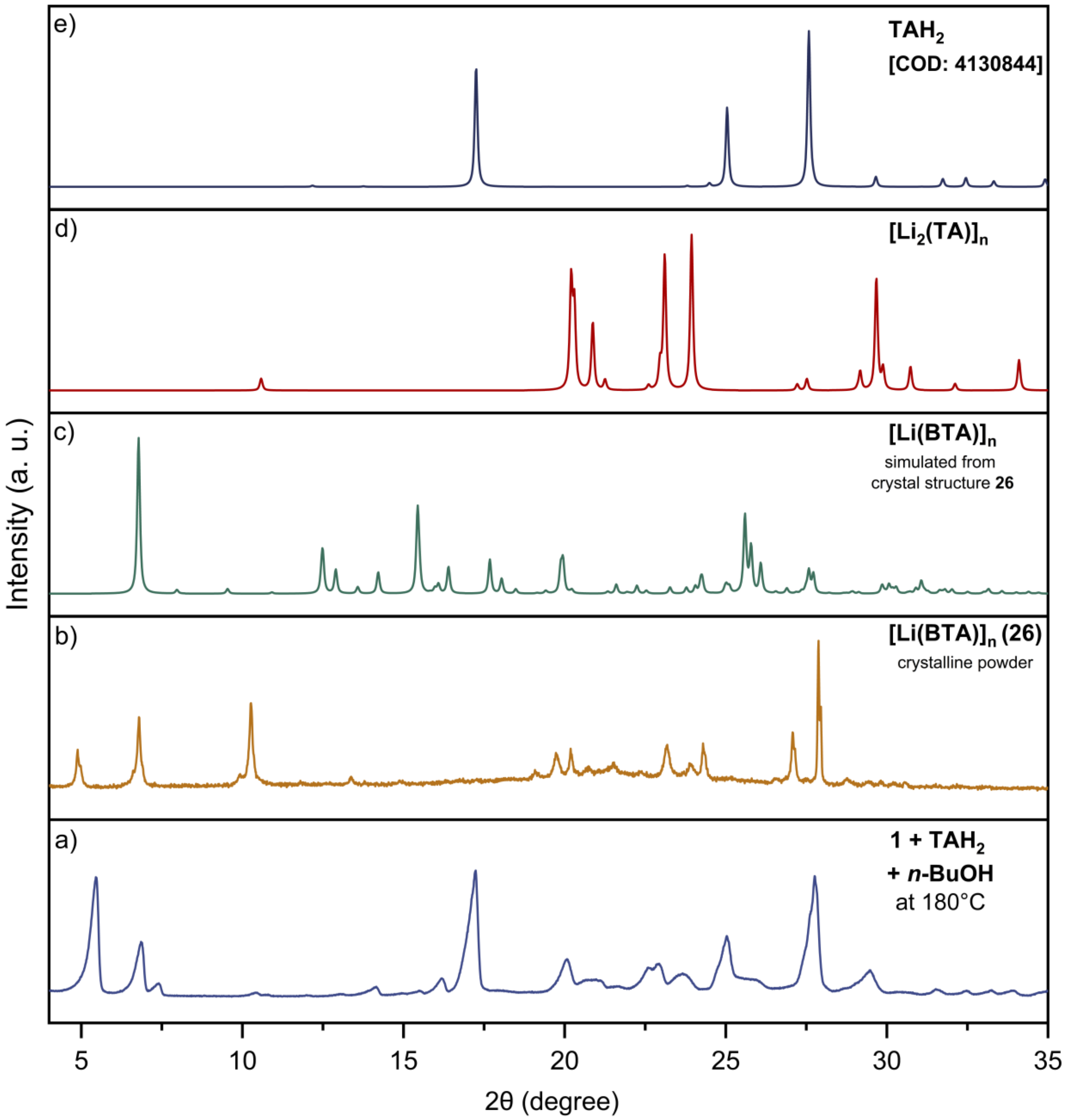


**Figure S59.** Comparison of PXRD patterns of a) **1+TAH$_2$+*n*-BuOH**, b) **[Li(BTA)]$_n$** (**26**), c) **[Li(BTA)]$_n$** (simulated), d) **[Li$_2$(TA)]$_n$**, and e) **TAH$_2$**.

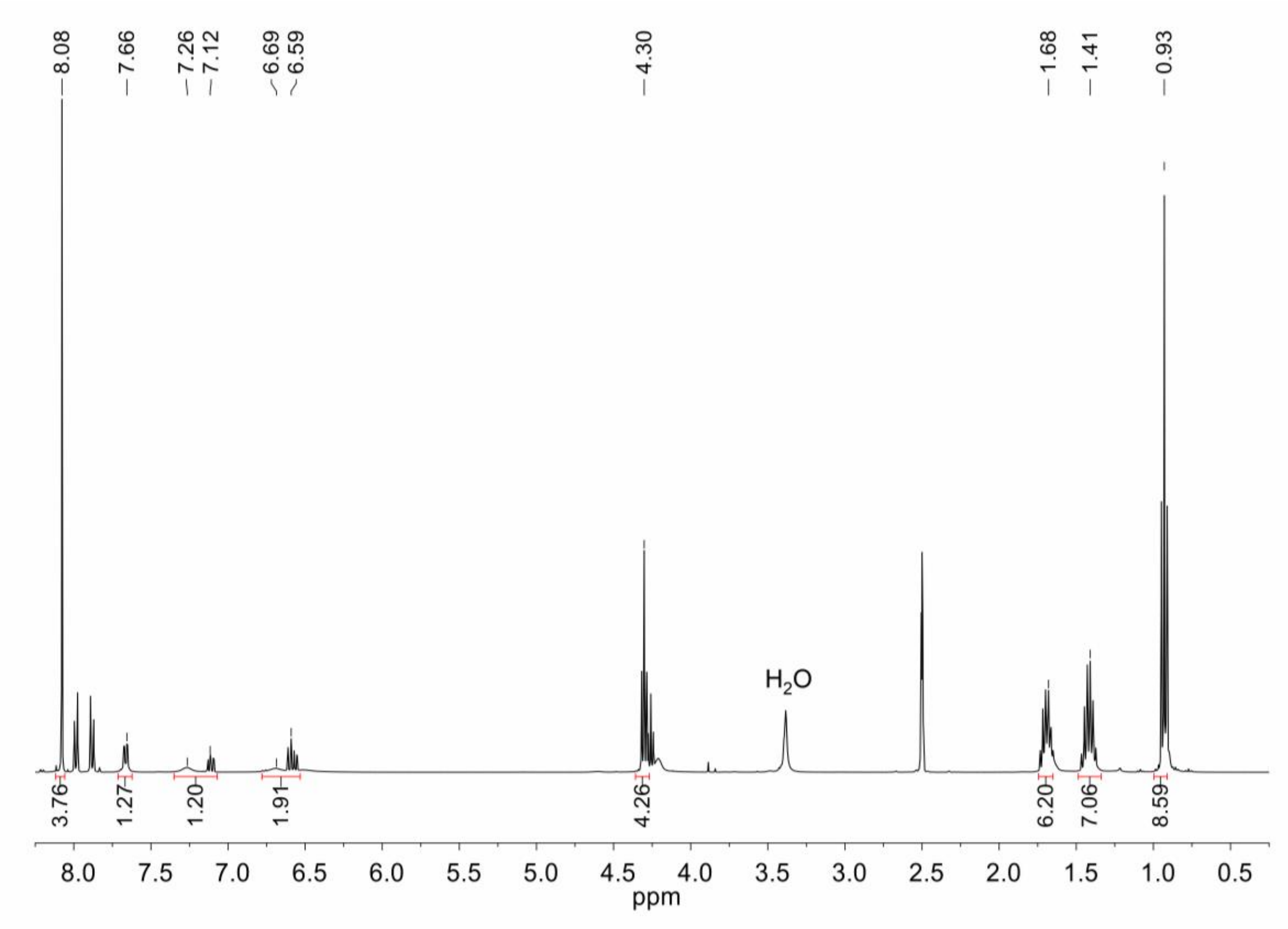


**Figure S60.** $^{1}$H NMR spectrum of **1 + DBT + *n*-BuOH** in DMSO-d$_6$.

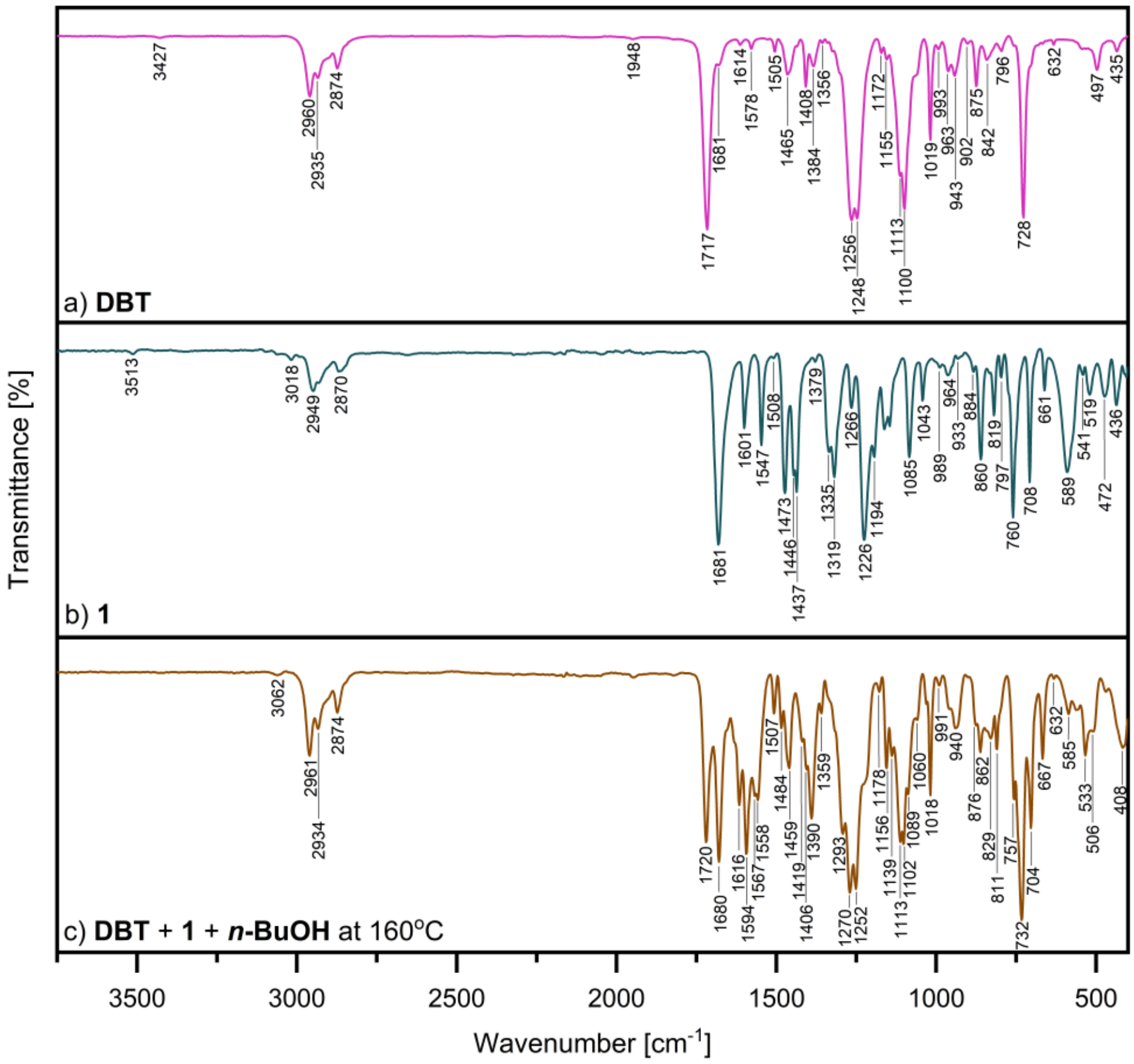


**Figure S61.** Comparison of FTIR-ATR spectrum products of reaction **1**, **DBT,** and *n*-BuOH with references of **1** and **DBT**.

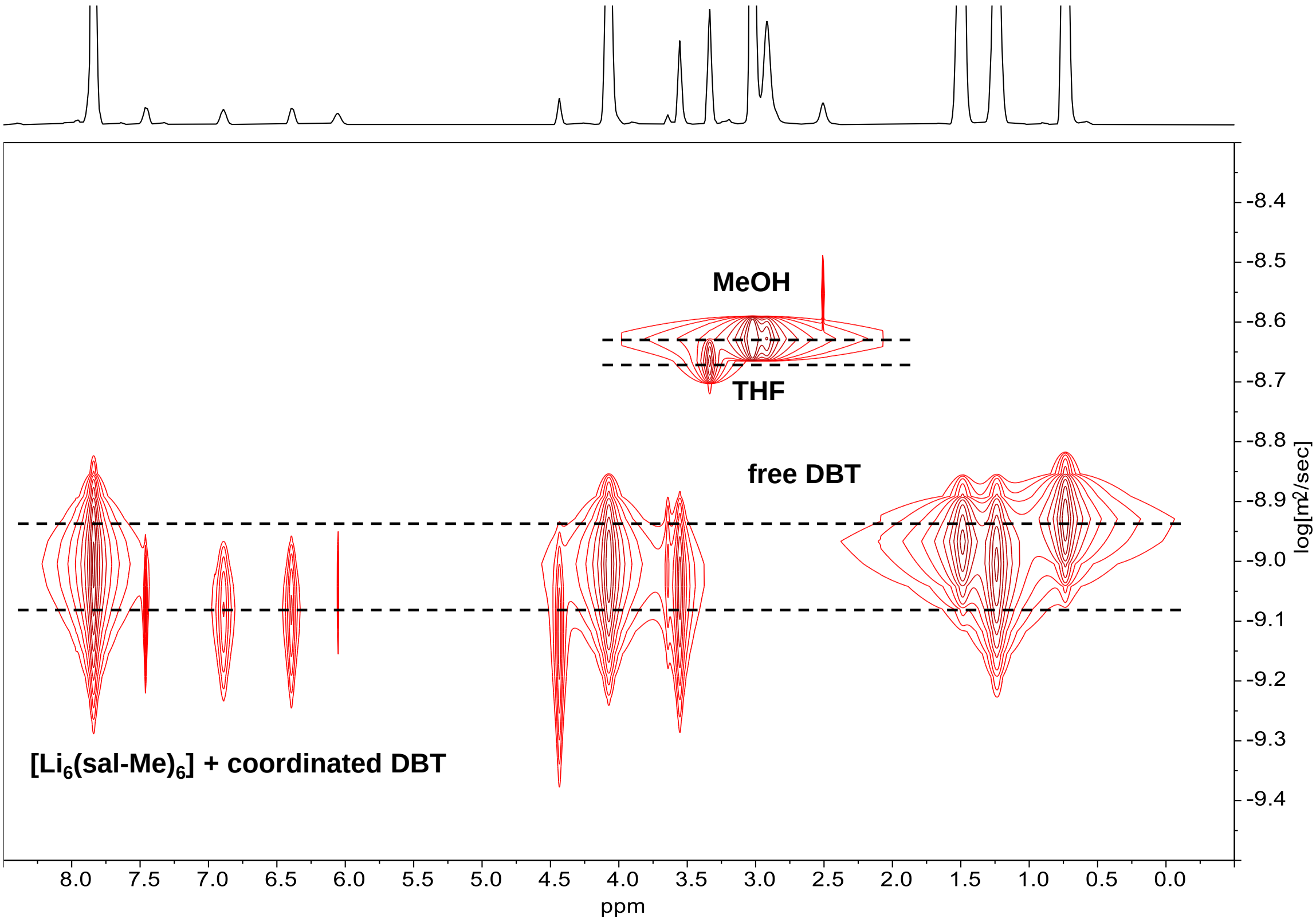


**Figure S62.** $^{1}$H-DOSY NMR spectrum of **1 + DBT + MeOH** in THF-$d_8$.

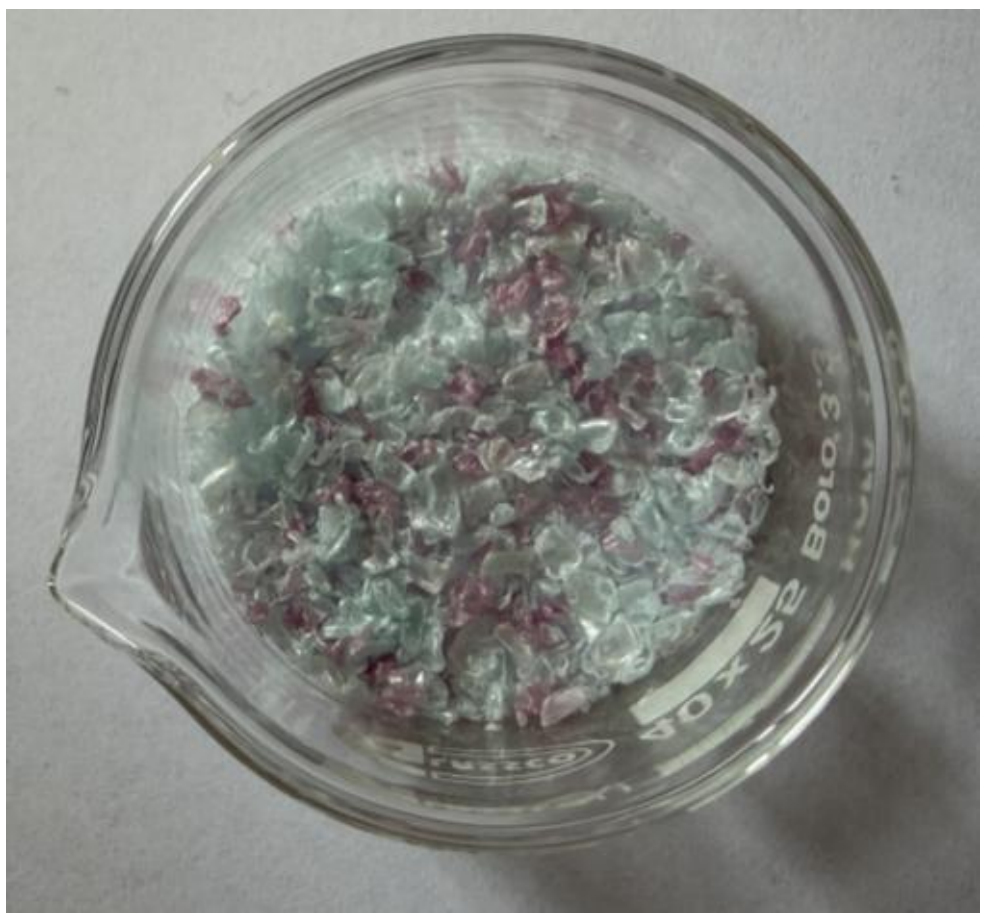

**Figure S63.** Mixture of PET bottle waste from four different manufacturers.

**Table S4.** Summary of alcoholysis of PET in the presence of **1-8**.[a]

| Entry | Catalyst | Temperature [°C] | Conversion of PET [%] |
|---|---|---|---|
| 1 | catalyst-free | 120 | 2 |
| 2 | | 140 | 4 |
| 3 | | 150 | 4 |
| 4 | | 160 | 6 |
| 5 | | 170 | 6 |
| 6 | | 180 | 11 |
| 7 | | 200 | 11 |
| 8 | $[Li_6(sal\text{-}Me)_6]$ (**1**) | 120 | 22 |
| 9 | | 140 | 71 |
| 10 | | 150 | 77 |
| 11 | | 160 | 99 |
| 12 | $[Na_6(sal\text{-}Me)_6]$ (**2**) | 140 | 20 |
| 13 | | 150 | 43 |
| 14 | | 160 | 47 |
| 15 | | 180 | 58 |
| 16 | | 200 | 100 |
| 17 | $[K_6(sal\text{-}Me)_6]$ (**3**) | 140 | 10 |
| 18 | | 150 | 15 |
| 19 | | 160 | 40 |
| 20 | | 180 | 57 |
| 21 | | 200 | 97 |
| 22 | $[Rb(sal\text{-}Me)]_n$ (**4**) | 140 | 15 |
| 23 | | 160 | 16 |
| 24 | | 170 | 22 |
| 25 | | 180 | 25 |
| 26 | | 200 | 68 |
| 27 | | 220 | 100 |
| 28 | $[Cs(sal\text{-}Me)]_n$ (**5**) | 140 | 14 |
| 29 | | 160 | 19 |
| 30 | | 170 | 37 |
| 31 | | 180 | 41 |
| 32 | | 200 | 80 |
| 33 | | 220 | 100 |
| 34 | $[Mg_2(sal\text{-}Et)_4(EtOH)_2]$ (**6**) | 140 | 2 |
| 35 | | 150 | 17 |
| 36 | | 160 | 33 |
| 37 | | 170 | 74 |
| 38 | | 180 | 99 |
| 39 | $[Zn_4(sal\text{-}Me)_8]$ (**7**) | 140 | 1 |
| 40 | | 160 | 11 |
| 41 | | 170 | 38 |
| 42 | | 180 | 64 |
| 43 | | 200 | 100 |
| 44 | $[Ca_3(sal\text{-}Me)_6(EtOH)_2]$ (**8**) | 140 | 2 |
| 45 | | 160 | 4 |
| 46 | | 170 | 51 |
| 47 | | 180 | 75 |
| 48 | | 200 | 100 |

[a]Alcoholysis conditions: [PET] = 0.438 mol/dm$^3$ (0.4 g, 2.08 mmol); [M/M′] = 0.022 mol/dm$^3$; V = 4.75 mL of n-butanol. Reactions were performed using pressure reactors with a capacity of 25 mL, with reactant stoichiometry [$TA_{(PET)}$/*n*-BuOH/M/M′] = 1/25/0.05, under autogenous pressure, at 120 to 220 ºC for 2 hours.

**Table S5.** Summary of alcoholysis of PET in the presence of **9**-1**8**.[a]

| Entry | Catalyst | Temperature [°C] | Conversion of PET [%] |
|---|---|---|---|
| 1 | $[Mg_2Li_2(sal\text{-}Me)_6]$ (**9**) | 120 | 9 |
| 2 | | 140 | 34 |
| 3 | | 150 | 81 |
| 4 | | 160 | 99 |
| 5 | | 180 | 100 |
| 6 | $[Mg_2Na_2(sal\text{-}Me)_6(THF)_4]$ (**10**) | 120 | 7 |
| 7 | | 140 | 41 |
| 8 | | 150 | 43 |
| 9 | | 160 | 64 |
| 10 | | 170 | 75 |
| 11 | | 180 | 94 |
| 12 | $[Mg_2K_2(sal\text{-}Me)_6](THF)_4]$ (**11**) | 120 | 15 |
| 13 | | 140 | 17 |
| 14 | | 150 | 46 |
| 15 | | 160 | 92 |
| 16 | | 180 | 100 |
| 17 | $[MgRb(\mu\text{-}H_2O)(sal\text{-}Me)_3(H_2O)_{0.5}]_n$ (**12**) | 140 | 1 |
| 18 | | 150 | 19 |
| 19 | | 160 | 28 |
| 20 | | 170 | 72 |
| 21 | | 180 | 90 |
| 22 | | 200 | 100 |
| 23 | $[Zn_2Li_2(sal\text{-}Me)_6]$ (**13**) | 140 | 26 |
| 24 | | 150 | 49 |
| 25 | | 160 | 64 |
| 26 | | 170 | 100 |
| 27 | $[Zn_2Na_2(sal\text{-}Me)_6(THF)_2]$ (**14**) | 120 | 13 |
| 28 | | 140 | 37 |
| 29 | | 150 | 66 |
| 30 | | 160 | 94 |
| 31 | | 180 | 100 |
| 32 | $[Zn_2K_2(sal\text{-}Me)_6(THF)_2]$ (**15**) | 120 | 22 |
| 33 | | 140 | 76 |
| 34 | | 150 | 77 |
| 35 | | 160 | 96 |
| 36 | | 180 | 100 |
| 37 | $[Ca_2Li_2(sal\text{-}Me)_6(THF)_2]$ (**16**) | 120 | 3 |
| 38 | | 140 | 55 |
| 39 | | 150 | 93 |
| 40 | | 160 | 97 |
| 41 | $[Ca_2Na_2(sal\text{-}Me)_6(MeOH)_4]$ (**17**) | 120 | 19 |
| 42 | | 140 | 33 |
| 43 | | 150 | 54 |
| 44 | | 160 | 58 |
| 45 | | 180 | 79 |
| 46 | $[Ca_2K_2(sal\text{-}Me)_6(MeOH)_4]$ (**18**) | 120 | 13 |
| 47 | | 140 | 39 |
| 48 | | 150 | 87 |
| 49 | | 160 | 94 |
| 50 | | 180 | 100 |

[a]Alcoholysis conditions: [PET] = 0.438 mol/dm$^3$ (0.4 g, 2.08 mmol); [M] = 0.022 mol/dm$^3$; V = 4.75 mL of *n*-butanol. Reactions were performed using pressure reactors with a capacity of 25 mL, with reactant stoichiometry [$TA_{(PET)}$/*n*-BuOH/M] = 1/25/0.05, under autogenous pressure, at 120 to 220 ºC for 2 hours.

**Table S6.** Summary of alcoholysis of PET in the presence of **19**-**25**.[a]

| Entry | Catalyst | Temperature [°C] | Conversion of PET [%] |
|---|---|---|---|
| 1 | $[Mg_4Na_2(sal\text{-}Me)_6(sal)_2(THF)_4]$ (**19**) | 140 | 5 |
| 2 | | 150 | 38 |
| 3 | | 160 | 57 |
| 4 | | 170 | 98 |
| 5 | | 180 | 100 |
| 6 | $[CaLi_6(sal\text{-}Me)_8]$ (**20**) | 120 | 10 |
| 7 | | 140 | 67 |
| 8 | | 150 | 100 |
| 9 | $[Ca_4Na(\mu_5\text{-}OH)(sal\text{-}Et)_8(EtOH)]$ (**21**) | 120 | 5 |
| 10 | | 140 | 27 |
| 11 | | 150 | 54 |
| 12 | | 160 | 91 |
| 13 | $[Ca_4K(\mu_5\text{-}OH)(sal\text{-}Et)_8(EtOH)]$ (**22**) | 120 | 12 |
| 14 | | 140 | 27 |
| 15 | | 150 | 41 |
| 16 | | 160 | 98 |
| 17 | $[ZnLi_2(sal\text{-}Me)_4(THF)_2]$ (**23**) | 120 | 6 |
| 18 | | 140 | 64 |
| 19 | | 150 | 90 |
| 20 | | 160 | 92 |
| 21 | | 180 | 99 |
| 22 | $[ZnNa_2(sal\text{-}Et)_4]_n$ (**24**) | 120 | 3 |
| 23 | | 140 | 48 |
| 24 | | 150 | 83 |
| 25 | | 160 | 100 |
| 26 | $[ZnK_2(sal\text{-}Me)_4]_n$ (**25**) | 120 | 22 |
| 27 | | 140 | 79 |
| 28 | | 150 | 92 |
| 29 | | 160 | 94 |

[a]Alcoholysis conditions: [PET] = 0.438 mol/$dm^3$ (0.4 g, 2.08 mmol); [M] = 0.022 mol/$dm^3$; V = 4.75 mL of *n*-butanol. Reactions were performed using pressure reactors with a capacity of 25 mL, with reactant stoichiometry [$TA_{(PET)}$/*n*-BuOH/M] = 1/25/0.05, under autogenous pressure, at 120 to 220 ºC for 2 hours.

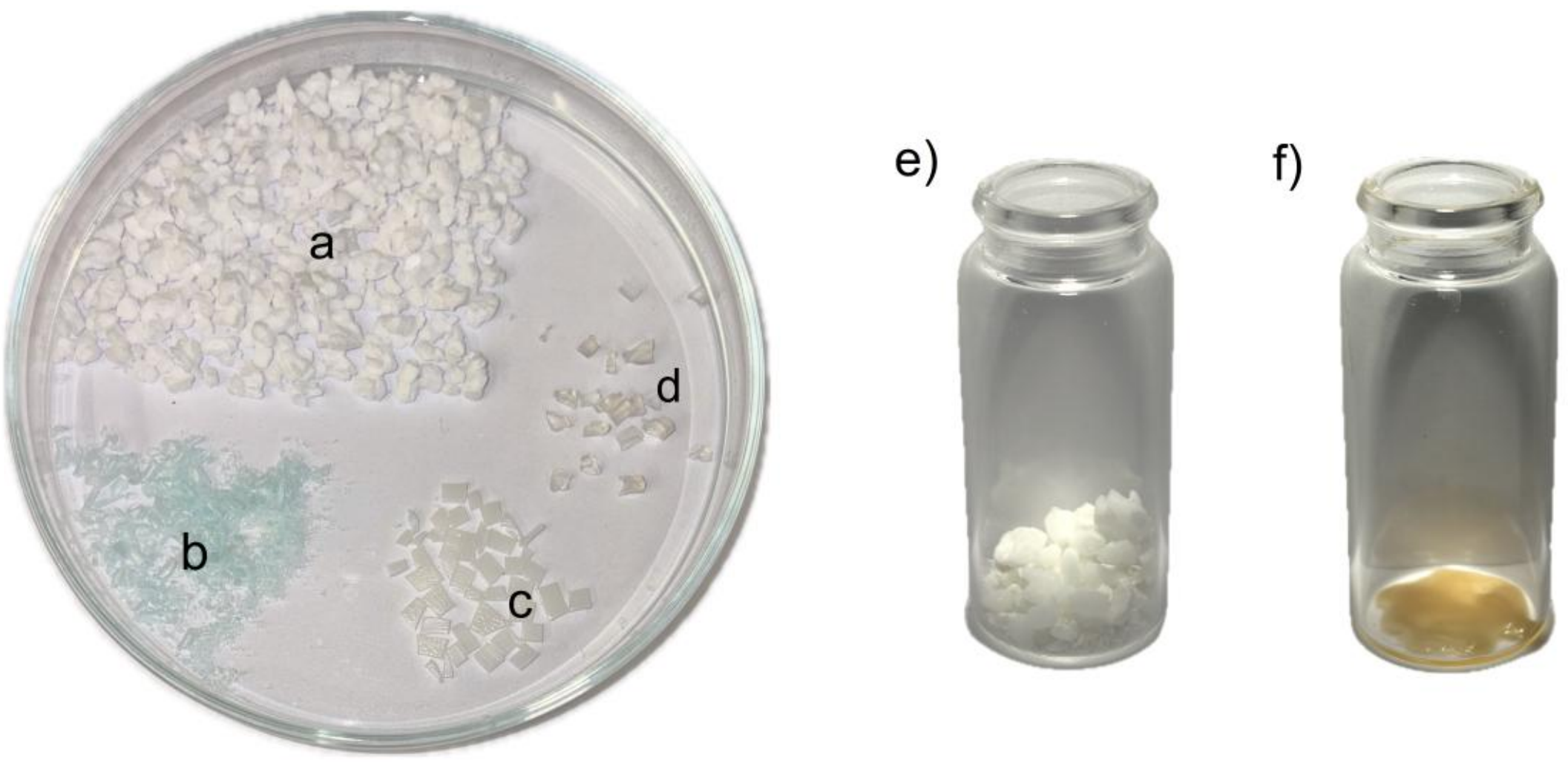


**Figure S64.** Polystyrene (PS) (a), PET (b), high-molecular-weight polyethylene (HDPE) (c), and high-consistency silicone rubber (HCR) (d) waste used in an alcoholysis reaction; post-reaction polymers' residues (e) and liquid mixture (f).

**Table S7.** Summary of alcoholysis of PEICT in the presence of **1-8**.[a,b]

| Entry | Catalyst | Temperature [°C] | Conversion of PEICT [%] |
|---|---|---|---|
| 1 | catalyst-free | 140 | 2 |
| 2 | | 160 | 4 |
| 3 | | 180 | 3 |
| 4 | | 200 | 7 |
| 5 | | 220 | 9 |
| 6 | | 240 | 18 |
| 7 | $[Li_6(sal\text{-}Me)_6]$ (**1**) | 120 | 29**[b]** |
| 8 | | 140 | 24/56**[b]** |
| 9 | | 150 | 73**[b]** |
| 10 | | 160 | 33/73**[b]** |
| 11 | | 180 | 35/85**[b]** |
| 12 | | 200 | 38 |
| 13 | | 220 | 35 |
| 14 | | 240 | 74 |
| 15 | $[Na_6(sal\text{-}Me)_6]$ (**2**) | 180 | 27 |
| 16 | | 200 | 33 |
| 17 | | 220 | 64 |
| 18 | | 240 | 64 |
| 19 | $[K_6(sal\text{-}Me)_6]$ (**3**) | 180 | 7 |
| 20 | | 200 | 30 |
| 21 | | 220 | 41 |
| 22 | | 240 | 65 |
| 23 | $[Rb(sal\text{-}Me)]_n$ (**4**) | 180 | 17 |
| 24 | | 200 | 33 |
| 25 | | 220 | 78 |
| 26 | | 240 | 87 |
| 27 | $[Cs(sal\text{-}Me)]_n$ (**5**) | 180 | 29 |
| 28 | | 200 | 40 |
| 29 | | 220 | 46 |
| 30 | | 240 | 74 |
| 31 | $[Mg_2(sal\text{-}Et)_4(EtOH)_2]$ (**6**) | 140 | 14 |
| 32 | | 160 | 40 |
| 33 | | 180 | 40 |
| 34 | | 200 | 100 |
| 35 | $[Zn_4(sal\text{-}Me)_8]$ (**7**) | 140 | 10 |
| 36 | | 160 | 27 |
| 37 | | 180 | 57 |
| 38 | | 200 | 91 |
| 39 | | 220 | 92 |
| 40 | | 240 | 100 |
| 41 | $[Ca_3(sal\text{-}Me)_6(EtOH)_2]$ (**8**) | 140 | 19 |
| 42 | | 160 | 33 |
| 43 | | 180 | 36 |
| 44 | | 200 | 90 |
| 45 | | 220 | 90 |
| 46 | | 240 | 100 |

[a]Alcoholysis conditions: $[TA_{(PEICT)}]$ = 0.427 mol/dm$^3$ (0.3 g, 1.21 mmol); [M/M′] = 0.011 mol/dm$^3$; V = 2.84 mL of *n*-butanol. Reactions were performed using pressure reactors with a capacity of 25 mL, with reactant stoichiometry $[TA_{(PEICT)}$/*n*-BuOH/M/M′] = 1/25/0.025, under autogenous pressure, at 140 to 240 ºC for 2 hours. [b]Reactant stoichiometry $[TA_{(PEICT)}$/*n*-BuOH/M′] = 1/25/0.05.

**Table S8.** Summary of alcoholysis of PEICT in the presence of **9-18**.[a]

| Entry | Catalyst | Temperature [°C] | Conversion of PEICT |
|---|---|---|---|
| 1 | $[Mg_2Li_2(sal\text{-}Me)_6]$ (**9**) | 140 | 10 |
| 2 | | 160 | 55 |
| 3 | | 180 | 77 |
| 4 | | 200 | 100 |
| 5 | $[Mg_2Na_2(sal\text{-}Me)_6(THF)_4]$ (**10**) | 140 | 36 |
| 6 | | 160 | 54 |
| 7 | | 180 | 55 |
| 8 | | 200 | 100 |
| 9 | $[Mg_2K_2(sal\text{-}Me)_6](THF)_4]$ (**11**) | 140 | 22 |
| 10 | | 160 | 46 |
| 11 | | 180 | 51 |
| 12 | | 200 | 88 |
| 13 | | 220 | 100 |
| 14 | $[MgRb(\mu\text{-}H_2O)(sal\text{-}Me)_3(H_2O)_{0.5}]_n$ (**12**) | 140 | 13 |
| 15 | | 160 | 22 |
| 16 | | 180 | 61 |
| 17 | | 200 | 97 |
| 18 | | 220 | 100 |
| 19 | $[Zn_2Li_2(sal\text{-}Me)_6]$ (**13**) | 140 | 16 |
| 20 | | 160 | 76 |
| 21 | | 180 | 100 |
| 22 | $[Zn_2Na_2(sal\text{-}Me)_6(THF)_2]$ (**14**) | 140 | 20 |
| 23 | | 160 | 57 |
| 24 | | 180 | 79 |
| 25 | | 200 | 99 |
| 26 | | 220 | 100 |
| 27 | $[Zn_2K_2(sal\text{-}Me)_6(THF)_2]$ (**15**) | 140 | 17 |
| 28 | | 160 | 98 |
| 29 | | 180 | 98 |
| 30 | | 200 | 100 |
| 31 | $[Ca_2Li_2(sal\text{-}Me)_6(THF)_2]$ (**16**) | 140 | 51 |
| 32 | | 160 | 59 |
| 33 | | 180 | 65 |
| 34 | | 200 | 100 |
| 35 | $[Ca_2Na_2(sal\text{-}Me)_6(MeOH)_4]$ (**17**) | 140 | 24 |
| 36 | | 160 | 49 |
| 37 | | 180 | 58 |
| 38 | | 200 | 97 |
| 39 | | 220 | 98 |
| 40 | | 240 | 100 |
| 41 | $[Ca_2K_2(sal\text{-}Me)_6(MeOH)_4]$ (**18**) | 140 | 37 |
| 42 | | 160 | 45 |
| 43 | | 180 | 65 |
| 44 | | 200 | 75 |
| 45 | | 220 | 100 |

[a]Alcoholysis conditions: $[TA_{(PEICT)}]$ = 0.427 mol/dm$^3$ (0.3g, 1.21 mmol); [M] = 0.011 mol/dm$^3$; V = 2.84 mL of *n*-butanol. Reactions were performed using pressure reactors with a capacity of 25 mL, with reactant stoichiometry $[TA_{(PEICT)}/n\text{-}BuOH/M]$ = 1/25/0.025, under autogenous pressure, at 140 to 220 ºC for 2 hours.

**Table S9.** Summary of alcoholysis of PEICT in the presence of **19-25**.[a]

| Entry | Catalyst | Temperature | Conversion of PEICT [%] |
|---|---|---|---|
| 1 | $[Mg_4Na_2(sal\text{-}Me)_6(sal)_2(THF)_4]$ (**19**) | 140 | 29 |
| 2 | | 160 | 55 |
| 3 | | 180 | 87 |
| 4 | | 200 | 100 |
| 5 | $[CaLi_6(sal\text{-}Me)_8]$ (**20**) | 140 | 66 |
| 6 | | 160 | 77 |
| 7 | | 180 | 87 |
| 8 | | 200 | 100 |
| 9 | $[Ca_4Na(\mu_5\text{-}OH)(sal\text{-}Et)_8(EtOH)]$ (**21**) | 140 | 46 |
| 10 | | 160 | 63 |
| 11 | | 180 | 74 |
| 12 | | 200 | 100 |
| 13 | $[Ca_4K(\mu_5\text{-}OH)(sal\text{-}Et)_8(EtOH)]$ (**22**) | 140 | 48 |
| 14 | | 160 | 64 |
| 15 | | 180 | 82 |
| 16 | | 200 | 100 |
| 17 | $[ZnLi_2(sal\text{-}Me)_4(THF)_2]$ (**23**) | 140 | 58 |
| 18 | | 160 | 89 |
| 19 | | 180 | 97 |
| 20 | | 200 | 100 |
| 21 | $[ZnNa_2(sal\text{-}Et)_4]_n$ (**24**) | 140 | 44 |
| 22 | | 160 | 80 |
| 23 | | 180 | 100 |
| 24 | $[ZnK_2(sal\text{-}Me)_4]_n$ (**25**) | 140 | 33 |
| 25 | | 160 | 72 |
| 26 | | 180 | 96 |
| 27 | | 200 | 100 |

[a]Alcoholysis conditions: $[TA_{(PEICT)}]$ = 0.427 mol/dm$^3$ (0.3g, 1.21 mmol); [M] = 0.011 mol/dm$^3$; V = 2.84 mL of *n*-butanol. Reactions were performed using pressure reactors with a capacity of 25 mL, with reactant stoichiometry $[TA_{(PEICT)}/n\text{-}BuOH/M]$ = 1/25/0.025, under autogenous pressure, at 140 to 200 ºC for 2 hours.

**Table S10.** Summary of alcoholysis of PET in the presence of simple inorganic compounds.[a]

| Entry | Catalyst | Conversion of PET [%] |
|---|---|---|
| 1 | $CH_3COOLi$ | 23 |
| 2 | $CH_3COONa·3H_2O$ | 3 |
| 3 | $CH_3COOK$ | 5 |
| 4 | $CH_3COORb$ | 2 |
| 5 | $CH_3COOCs$ | 4 |
| 6 | $Mg(CH_3COO)_2·4H_2O$ | 3 |
| 7 | $Ca(CH_3COO)_2$ | 2 |
| 8 | $Zn(CH_3COO)_2·2H_2O$ | 5 |
| 9 | $Li_2CO_3$ | 4 |
| 10 | $Na_2CO_3$ | 1 |
| 11 | $K_2CO_3$ | 8 |
| 12 | $CaCO_3$ | 3 |
| 13 | Li(OMe) | 19 |
| 14 | Na(OMe) | 13 |
| 15 | K(OMe) | 14 |
| 16 | $Mg(OMe)_2$ | 2 |
| 17 | $Ca(OMe)_2$ | 5 |
| 18 | $Zn(OMe)_2$ | 8 |
| 19 | LiOH | 8 |
| 20 | NaOH | 10 |
| 21 | KOH | 13 |
| 22 | $ZnCl_2$ | 3 |

[a]Alcoholysis conditions: [PET] = 0.438 mol/dm$^3$ (0.4 g, 2.08 mmol); [M] = 0.022 mol/dm$^3$; V = 4.75 mL of *n*-butanol. Reactions were performed using pressure reactors with a capacity of 25 mL, with reactant stoichiometry [$TA_{(PET)}$/*n*-BuOH/M] = 1/25/0.05, under autogenous pressure, at 150ºC for 2 hours.

**Table S11**. Summary of alcoholysis of a mixture of PET bottle waste from four different manufacturers using **1**, **16**, **20** and **25** as catalysts.[a]

| Entry | Catalyst | Temperature [°C] | Conversion of PET [%] |
|---|---|---|---|
| 1 | $[Li_6(sal\text{-}Me)_6]$ (**1**) | 150 | 70 |
| 2 | | 160 | 88 |
| 3 | $[Ca_2Li_2(sal\text{-}Me)_6(THF)_2]$ (**16**) | 150 | 83 |
| 4 | | 160 | 96 |
| 5 | $[CaLi_6(sal\text{-}Me)_8]$ (**20**) | 150 | 100 |
| 6 | $[ZnK_2(sal\text{-}Me)_4]_n$ (**25**) | 150 | 88 |
| 7 | | 160 | 99 |

[a]Alcoholysis conditions: [PET] = 0.438 mol/dm$^3$ (0.4 g, 2.08 mmol); [M] = 0.022 mol/dm$^3$; V = 4.75 mL of *n*-butanol. Reactions were performed using pressure reactors with a capacity of 25 mL, with reactant stoichiometry $[TA_{(PET)}$/*n*-BuOH/M] = 1/25/0.05, under autogenous pressure, at 150ºC and 160ºC for 2 hours.

---

[1] Andersen, E.; Mikkelsen, R.; Kristiansen, S.; Hinge, M. Accelerated physical ageing of poly(1,4-cyclohexylenedimethylene-co-2,2,4,4-tetramethyl-1,3-cyclobutanediol terephthalate). *RSC Adv.* **2019**, *9* (25), 14209–14219. https://doi.org/10.1039/c9ra00925f.
